# Metaphyseal trauma of the lower extremities in major orthopedic surgery as an independent risk factor for deep vein thrombosis

**DOI:** 10.1007/s00590-024-03960-4

**Published:** 2024-05-23

**Authors:** Franky Hartono, Tessi Ananditya, Yohanes Augustinus, Hendra, Nicholas Gabriel

**Affiliations:** 1 Orthopaedic and Traumatology Department, Siloam Hospitals Kebon Jeruk, Jl. Perjuangan No.8, RT.14/RW.10, Kb. Jeruk, Kec. Kb. Jeruk, West Jakarta, Daerah Khusus, Ibukota, Jakarta 11530 Indonesia; 2 Orthopaedic and Traumatology Department, Pantai Indah Kapuk Hospital, Jakarta, Indonesia

**Keywords:** Major orthopedic surgery, Deep vein thrombosis, Metaphyseal trauma

## Abstract

**Purpose:**

Major orthopedic surgeries of the lower extremities, which heavily injure the metaphyseal region, are strongly associated with the risk of developing deep vein thrombosis (DVT). This study aims to investigate the role of metaphyseal trauma as an independent risk factor for DVT.

**Methods:**

Patients undergoing major orthopedic surgery of the hip and knee had their existing DVT risk factors recorded. Metaphyseal trauma was defined by the extent of bone injury during these surgeries. The samples were categorized into three surgery groups: total arthroplasty group (TA), hemiarthroplasty group (HA), and the open reduction internal fixation group (ORIF). Logistic regression test between significant existing risk factors and surgery groups determines the independent association between risk factors and DVT.

**Result:**

The study found a 24.8% incidence of asymptomatic DVT in patients undergoing major orthopedic surgeries, with the highest prevalence (37.2%) in TA, which had the largest extent of metaphyseal trauma and the least existing DVT risk factors. TA showed 6.2 OR and 95% CI (*p* = 0.036) compared to the other existing risk factor in relation to DVT incidence.

**Conclusion:**

Metaphyseal bone trauma in the hip and knee major orthopedic surgery is an independent risk factor for deep vein thrombosis.

**Supplementary Information:**

The online version contains supplementary material available at 10.1007/s00590-024-03960-4.

## Introduction

Deep vein thrombosis (DVT) is a blood clot that forms within the deep veins, most commonly in the lower limbs with clot formation originating in a deep calf vein propagating proximally [1]. As a part of venous thromboembolism (VTE) disorder, DVT represents the third most common mortality cause of cardiovascular disease due to pulmonary embolism. DVT occurs frequently as a postoperative complication, particularly after major lower limb orthopedic surgeries, a risk recognized previously by the *American College of Chest Physicians* (ACCP) and the *American Academy of Orthopaedic Surgeon* (AAOS) [2, 3].

Hip arthroplasty, knee arthroplasty, as well as hip or knee fracture surgeries are strongly associated with a risk of developing DVT [2, 4–6]. These surgeries primarily injured the metaphyseal region of the lower extremities bone [7]. The amount of endothelial damage in a heavily vascularized structure injury as in metaphyseal trauma increases the risk of DVT. Endothelial damage is one of the factors in Virchow’s triad (1856). In contrast, in cases of acute trauma in the diaphysis involving long bone such as a fracture of midshaft femur, fat emboli are more likely to occur due to the intravasation of bone marrow fat [8, 9].

Despite numerous studies on potential risk factors for DVT, there has been no published research on the impact of metaphyseal trauma as a risk factor for DVT. Therefore, the primary objective of this study was to analyze metaphyseal trauma as a potential risk factor for DVT. We hypothesize that trauma to the metaphyseal cancellous bone in the lower extremities is an independent risk factor for DVT after major lower orthopedic surgery.

## Methods

An observational prospective cohort was conducted during a 28-month period from 2008 to 2011. Inclusion criteria were patients aged above 50 years old and who were admitted to the hospital for major hip and knee surgery without chemical or mechanical thromboprophylaxis. The major lower orthopedic surgeries included were total knee arthroplasty (TKA), total hip arthroplasty (THA), hip hemiarthroplasty (HHA), as well as proximal and distal femur fixation (FF). All surgical procedures were performed by a single senior orthopedic surgeon in two private hospitals. Exclusion criteria were total paralysis patients, patients with central vein catheter, patients under estrogen treatment, post major operation patients within the last 3 months, patients with previous DVT or other rare conditions such as nephrotic syndrome, and inflammatory bowel disease.

All patients were hospitalized on an average of 8 to 12 days. Ambulatory observation was conducted for up to 30 postoperative days. Any anticoagulant treatments were stopped one week before the surgical procedures. DVT was confirmed as positive by venography or alternative USG color Doppler on the 7th to 8th postoperative day defined by fresh thrombus in the deep vein of the lower extremity. Patients with confirmed positive DVT were treated with an oral anticoagulant.

**Fig. 1:**
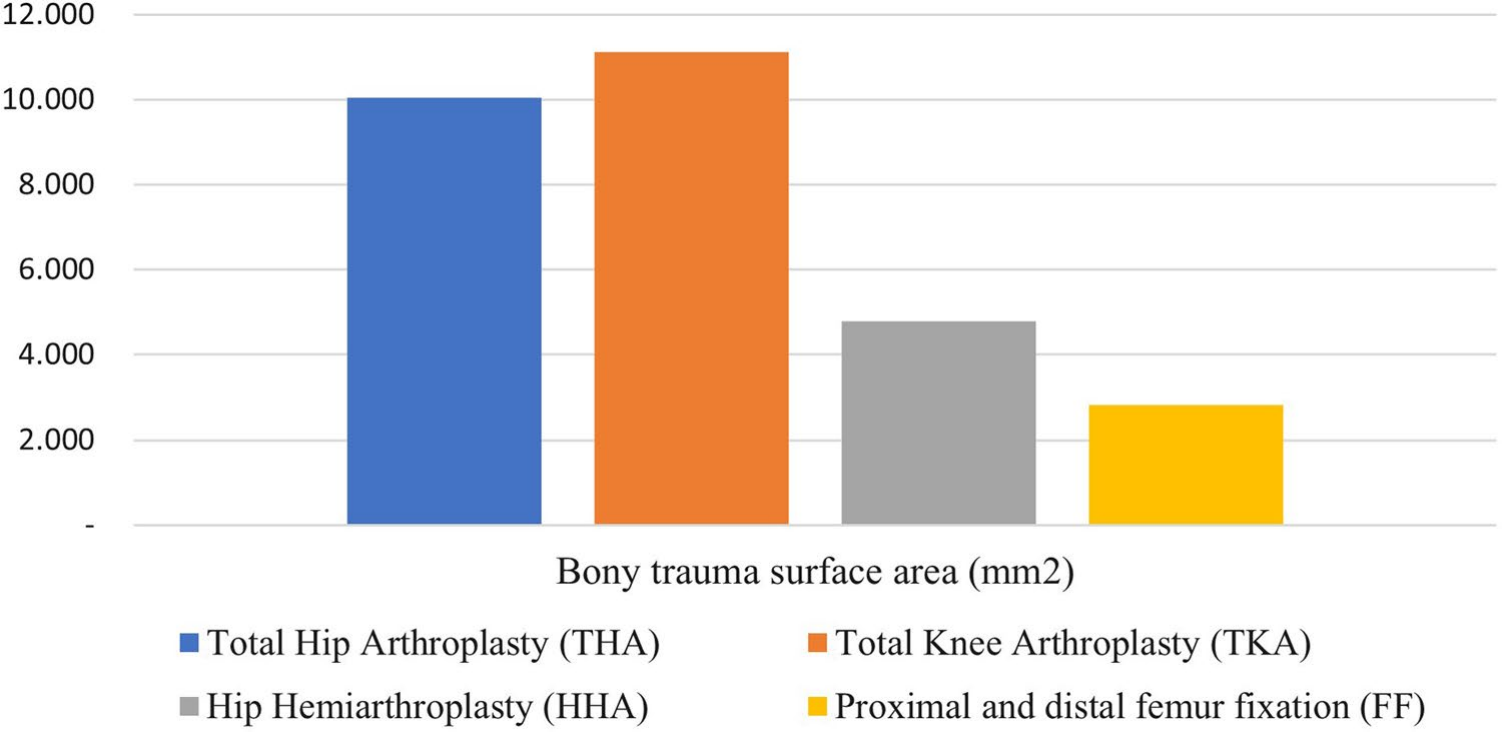
The surface area of metaphyseal trauma according to the type of orthopedic surgeries

Metaphyseal trauma was defined by the extent of bone injury during major hip and knee surgery. Although this area can certainly vary, we estimate the area of bony trauma by calculating the implant surface area of THA, TKA, and HHA. In cases of proximal and distal femur fractures, we quantify the implant surface area and the estimated surface area of the average existing fracture line. Bone traumatization surface area was calculated with AutoCAD 2018 (Fig. 1).

When each trauma surface was compared, we found that the extent of metaphyseal trauma measured in TKA and THA is nearly identical and they had the largest traumatic surface area, while the plate and screw implant had the least traumatic surface area.

Based on this finding, we divided the samples into three surgery groups according to the extent of metaphyseal trauma, namely the total arthroplasty group (TA), comprised of TKA and THA; the hip hemiarthroplasty group (HA) comprised of HHA; and the open reduction and internal fixation group (ORIF) comprised of FF.

The existing DVT risk factors of each patient were recorded and listed according to the surgery group (Table 1). Various risk factors were analyzed to the DVT incidence and compared between each surgery group (Table 2). Data were evaluated by Chi-square test or Fisher’s exact test. A *p* value < 0.05 was considered statistically significant.

**Table 1:** The existing risk factors in the three surgery groups

Risk factors	TA *n* = 43	HA *n* = 32	ORIF *n* = 26	Total *n* = 101
Age, > 70/50–70 years, *n* (%)	19 (44.2)/24 (55.8)	25 (78.1)/7 (21.9)	18 (69.2)/8 (30.8)	62 (61.4)/39 (38.6)
BMI, ≥ 25/ < 25 kg/m^2^, *n* (%)	23 (53.5)/20 (46.5)	4 (12.5)/28 (87.5)	5 (19.2)/21 (80.8)	32 (31.7)/69 (68.3)
Female/Male, *n* (%)	35 (81.4)/8 (18.6)	26 (81.3)/6 (18.8)	15 (57.7)/11 (42.3)	76 (75.2)/25 (24.8)
Malignancy, *n* (%)	1 (2.3)	2 (6.30)	1 (3.8)	4 (4.0)
Hypertension, *n* (%)	18 (41.9)	17 (53.1)	16 (61.5)	51 (50.5)
Diabetes mellitus, *n* (%)	4 (9.3)	7 (21.9)	7 (26.9)	18 (17.8)
Cardiac dysfunction, *n* (%)	7 (16.3)	5 (15.6)	5 (19.2)	17 (16.8)
CKD, *n* (%)	1 (2.3)	2 (6.3)	2 (7.7)	5 (5.0)
Stroke history, *n* (%)	1 (2.3)	6 (18.8)	3 (11.5)	10 (9.9)
Smoking, *n* (%)	0 (0)	3 (9.4)	1 (3.8)	4 (4.0)
Thalassemia, *n* (%)	0 (0)	1 (3.1)	1 (3.8)	2 (2.0)
HDL, ≤ 40 / > 40 mg/dl, *n* (%)	7 (16.3)/36 (83.7)	9 (28.1)/23 (71.9)	8 (30.8)/18 (69.2)	24 (23.8)/77 (76.2)
LDL, ≥ 130 / < 130 mg/dl, *n* (%)	20 (46.5)/23 (53.5)	9 (28.1)/23 (71.9)	6 (23.1)/20 (76.9)	35 (34.7)/66 (65.3)
Triglyceride, > 150 / ≤ 150 mg/dl, *n* (%)	10 (23.3)/33 (76.7)	5 (15.6)/27 (84.4)	4 (15.4)/22 (84.6)	19 (18.8)/82 (81.2)
Cholesterol total, > 200 / ≤ 200 mg/dl, *n* (%)	22 (51.2)/21 (48.8)	10 (31.3)/22 (68.8)	6 (23.1)/20 (76.9)	38 (37.6)/63 (62.4)
Fibrinogen D-1, high/N, *n* (%)	10 (23.3)/33 (76.7)	14 (43.8)/18 (56.3)	6 (23.1)/20 (76.9)	30 (29.7)/71 (70.3)
Fibrinogen D + 7, high/N, *n* (%)	37 (86.0)/6 (14.0)	19 (59.4)/13 (40.6)	17 (65.4)/9 (34.6)	73 (72.3)/28 (27.7)
D-dimer D-1, high/N, *n* (%)	9 (20.9)/34 (79.1)	18 (56.3)/14 (43.8)	20 (76.9)/6 (23.1)	47 (46.5)/54 (53.5)
D-dimer D + 7, high/N, *n* (%)	36 (83.7)/7 (16.3)	17 (53.1)/15 (46.9)	17 (65.4)/9 (34.6)	71 (70.3)/30 (29.7)
Surgery duration, minute, med/min–max	152.14/70–340	76.25/55–210	106.67/45–180	119.38/45–340
Surgery duration, > 120 / ≤ 120 min, *n* (%)	38 (88.4)/5 (11.6)	2 (6.3)/30 (93.7)	7 (26.9)/19 (73.1)	47 (46.5)/54 (53.5)
Total blood loss 3 days, ml, med/min–max	605.0/250–2259	500.0/220–1565	265.0/30–815	505.0/30–2259
Total blood loss 3 days ≥ 300 / < 300 ml, *n* (%)	41 (95.3)/2 (4.7)	29 (90.6)/3 (9.4)	12 (46.2)/14 (53.8)	82 (81.2)/19 (18.8)

*TA* Total arthroplasty, *HA* Hemiarthroplasty, *ORIF* Open reduction and internal fixation, *BMI* body mass index, *CKD* chronic kidney disease, *HDL* high-density lipoprotein, *LDL* l-density lipoprotein, *N* normal, med median, *D-1* one day before surgery, *D+7* 7 days after surgery, min minimum, max maximum

**Table 2:** The relationship of the existing risk factors to DVT incidence in the three surgery groups

Risk factors	TA *n* = 43		HA *n* = 32		ORIF *n* = 26		Total *n* = 101	
	OR	CI 95%	OR	CI 95%	OR	CI 95%	OR	CI 95%
Age ≥ 71/50–70 years, *n* (%)	2.1	0.6–7.7	1.1	0.1–12.2	1.4	0.1–15.9	1.1	0.4–2.9
BMI ≥ 25 / < 25 kg/m^2^, *n* (%)	1.1	0.3–4.1	1.1**	1.0–1.3	1.5	0.1–18.4	1.6	0.6–4.1
Female/Male, *n* (%)	0.9	0.2–4.8	1.2**	1.0–1.5	2.5	0.2–27.9	2.0	0.6–6.5
Malignancy, yes/no, *n* (%)	0.9	0.8–1.0	6.5	0.3–126.0	1.0**	0.9–1.1	3.2	0.4–24.1
Hypertension, yes/no, *n* (%)	3.9*	1.0–14.6	4.3	0.4–43.7	2.0	0.1–23.2	2.6*	1.0–6.8
Diabetes mellitus, yes/no, *n* (%)	0.5	0.1–5.6	0.8	0.1–9.3	3.4	0.3–30.6	0.8	0.2–2.8
Cardiac dysfunction, yes/no, *n* (%)	2.6	0.5–13.8	5.3	0.6–45.9	1.5	0.1–18.4	2.5	0.8–7.6
CKD, yes/no, *n* (%)	0.9	0.8–1.0	1.0**	0.9–1.2	7.0	0.3–144.0	2.1	0.3–13.4
Stroke, yes/no, *n* (%)	0.9	0.8–1.0	1.1	0.1–12.0	1.1**	0.9–1.3	0.7	0.1–3.7
Smoking, yes/no, *n* (%)	–	–	1.1**	0.9–1.2	0.7**	0.4–1.3	1.0	0.1–10.2
Thalassemia, yes/no, *n* (%)	–	–	0.8**	0.5–1.2	0.7**	0.4–1.3	4.3^**^	3.0–6.1
HDL, ≤ 40 / > 40 mg/dl (%)	1.3	0.2–6.8	5.2	0.7–39.0	10.2	0.8–120.9	2.2	0.8–6.1
LDL, ≥ 130 / < 130 mg/dl (%)	2.8	0.7–10.1	0.5	0.1–6.1	1.3**	1.0–1.7	1.7	0.6–4.2
Triglyceride, > 150 / ≤ 150 mg/dl (%)	1.1	0.2–4.9	1.4	0.1–16.4	2.0	0.1–27.5	1.5	0.5–4.5
Cholesterol total, > 200 / ≤ 200 mg/dl (%)	1.3	0.3–4.8	0.5	0.1–5.1	1.3**	1.0–1.7	1.1	0.4–2.8
Fibrinogen D-1 high/N, *n* (%)	1.1	0.2–4.9	0.2	0.1–2.7	1.1	0.1–13.4	0.6	0.2–1.9
Fibrinogen D + 7 high/N, *n* (%)	3.4	0.3–32.1	1.0	0.1–7.2	0.4	0.1–4.0	1.7	0.5–5.1
D-dimer D-1 high/N, *n* (%)	0.4	0.1–2.2	1.2	0.1–8.3	0.8	0.1–10.4	0.4	0.1–1.1
D-dimer D + 7 high/N, *n* (%)	1.5	0.2–9.3	4.3	0.4–43.7	1.6**	1.1–2.3	4.0*	1.1–14.0
Surgery duration, > 120 / ≤ 120 min, *n* (%)	2.6	0.2–25.6	1.0**	0.9–1.2	0.8	0.1–10.3	2.5*	1.0–6.5
Total blood loss 3 days ≥ 300 / < 300 ml, *n* (%)	0.5	0.1–9.9	1.1**	0.9–1.2	1.2	0.1–10.1	1.9	0.5–7.6

*p < 0.05

**Relative risk

*TA* Total arthroplasty, *HA* Hemiarthroplasty, *ORIF* open reduction and internal fixation, *DVT* deep vein thrombosis, *BMI* body mass index, *CKD* chronic kidney disease, *HDL* high-density lipoprotein, *LDL* low-density lipoprotein, *N* normal, *D-1* one day before surgery, *D + 7* 7 days after surgery, min minimum, max maximum

To identify the role of metaphyseal trauma as an independent risk factor for DVT, we conducted a multivariate logistic regression test between the existing risk factors and the surgery groups (TA and HA or ORIF) in association with DVT incidence (Table 3), where the extent of metaphyseal trauma was represented by the surgery groups.

**Table 3:** The relationship between the three surgery groups and the incidence of DVT compared with the adjusted existing risk factors

Risk factors	Sig	OR	CI 95%
Total arthroplasty	0.036	6.205	1.127–34.162
Hemiarthroplasty/ORIF	0.883	1.123	0.242–5.218
CKD	0.507	0.507	0.054–4.788
Stroke	0.540	1.901	0.244–14.821
Cardiac dysfunction	0.569	0.653	0.150–2.835
Diabetes mellitus	0.782	1.242	0.268–5.759
Hypertension	0.054	0.301	0.089–1.023
Malignancy	0.154	0.171	0.15–1.936
D-dimer D + 7 high	1.000	0.708	1.000–1.000
Surgery duration, > 120	0.998	0.767	0.984–1.012
3 days total blood loss > 300 ml	0.998	0.239	0.996–1.001

*Sig* significances, *OR* Odds Ratio, *ORIF* open reduction and internal fixation, *CKD* chronic kidney disease, *D + 7* 7 days after surgery

All tests in this study were analyzed by using IBM SPSS Statistics 23. This study had been approved by the boards of the ethical committee of the two hospitals where the study was conducted with approval number 0584/RSPIK-DIR/VIII/08 and 0378/H04.8.4.5.31/PP36-KOMETIK/2010.

## Result

From a total of 167 patients who underwent the procedures, 101 patients met the inclusion criteria. Twenty-five patients (24.8%) were found asymptomatic DVT positive. Where 16 patients out of 43 (37.2%) from TA, 5 patients out of 32 (15.6%) from HA and 4 patients out of 26 (15.4%) from ORIF were DVT positive. TA consisted of 43 non-emergency osteoarthrosis patients (42.6%) who underwent TKA and THA. HA consisted of 32 emergency sub-capital femur fracture patients (31.7%) treated with Austin Moore Hip Prosthesis (AMP) or Bipolar Hip Prosthesis (BHP) implants. Moreover, ORIF consisted of 26 emergency trochanteric and condylar femur fracture patients (25.7%), treated with either dynamic hip screws (DHS), condylar blade plate (CBP), proximal femur locking plate (PFLP) or cannulated screws (CS) implants.

The recorded existing risk factor from total patients was found to be dominated by older age > 70 years (61.4%), slender patients with BMI < 25 kg/m^2^ (68.3%), female (75.2%), hypertension (50.5%), 3 days total blood loss > 300 ml (81.2%), and high 7th day post-op D-dimer (70.3%) (Table 2). These risk factors were found mostly in emergency/acute trauma patients within the HA and ORIF.

From the bivariate analysis of the existing risk factors to DVT incidence in the three surgery groups, we found that the significant risk factors in each group were: hypertension in the TA group, high BMI and female gender in the HA group, and high LDL, HDL, and high D-dimer levels on the 7th day post-operation in the ORIF group. Meanwhile, when considering the total number of patients from all groups, significant risk factors included hypertension (*p* = 0.04, OR = 2.6), high D-Dimer on the 7th day post-op (*p* = 0.02, OR = 4.0), and long duration of surgery (*p* = 0.04, OR = 2.5) (Table 3).

The adjusted OR of the multivariate logistic regression test between the existing risk factors and the surgery groups (TA and HA or ORIF) showed for TA is 6.2 with a 95% CI (p = 0.036) in relation to the incidence of DVT. The other risk factors were not found to be significant except for hypertension (*p* = 0.05), although the OR was only 0.301 (Table 3).

## Discussion

In this cohort study, we found that TA group have a sixfold increased risk of developing DVT (OR 6.2, *p* = 0.036). TA, comprised of TKR and THR, represents the largest extent of metaphyseal trauma (Fig. 1) that occurs in the hip and knee major orthopedic surgery. Apart from hypertension (OR 0.31, *p* = 0.054), the other risk factors were not significantly associated with an increased risk of DVT. This strongly implies that the metaphyseal trauma during hip and knee major orthopedic surgery itself has a role in DVT occurrence.

The distinguishing factor between major orthopedic surgery in hip and knee, as opposed to the other region, is the extent of trauma in the metaphysis. Hip fracture surgery, hip arthroplasty, and knee arthroplasty are strongly associated with the risk of developing DVT, with an incidence rate of 30–80% for asymptomatic DVT and 0.5–4% for symptomatic DVT [10, 11]. These surgeries primarily injured the heavily vascularized metaphyseal region of the lower extremities through bone cutting and reaming procedures [7]. Initial studies from the author have also demonstrated that metaphyseal bone trauma is linked with marked changes in thrombogenic biomarkers post-operatively and influence the incidence of DVT [12, 13]. Recent studies from Asian demographic also showed that simultaneous bilateral TKA increased the risk of DVT [14, 15]. Moreover, a large nationwide study by Seung et al. concluded TKA as one of a risk factor for developing postoperative DVT [10].

Concluding based on the previous statements, it can be implied that surgeries in the diaphyseal region cause less endothelial damage. Diaphyseal trauma is more commonly associated with the development of fat emboli [16]. For this reason, we mainly highlight the impact of metaphyseal trauma, rather than comparing between metaphyseal and diaphyseal trauma, as a risk factor for DVT.

Surgery is associated with several prothrombotic processes, which can be one or more components of Virchow's triad (venous stasis, endothelial damage, and hypercoagulability). Despite major orthopedic surgery having long been known as a risk factor for DVT [1], to our knowledge, the role of metaphyseal trauma as a risk factor in lower extremities, major orthopedic surgery on DVT incidence has not been proven before.

We measured the extent of metaphyseal trauma in our sample. Based on the measurement of metaphyseal trauma surface area, we observed that the largest area of metaphyseal trauma occurs in the TA (Fig. 1). This can be attributed to the extensive bone cutting and reaming involved in implant preparation during total arthroplasty of the hip and knee, which was performed to a lesser extent in the HA and ORIF.

From the patient demographic, we observed consistent findings with previous studies, with 24.8% of patients who underwent major orthopedic surgeries developing asymptomatic DVT, and consequently, the majority was TA patients (Table 1). It is worth noting that in our sample, the recorded existing DVT risk factors in the TA group were fewer compared to the other groups; however, they still had the highest incidence of DVT.

The existing risk factors in our sample were dominated by older age, female gender, hypertension, significant blood loss, and elevated D-dimer levels on the 7th day post-operation (Table 1). However, when we compare the existing risk factor with the incidence of DVT, the significant risk factors were indeed hypertension, high D-dimer levels, and longer surgical duration (*p* < 0.05) (Table 2). This aligns with previous research where patients with hypertension showed a twofold increased likelihood of developing DVT [17], while longer surgical durations were associated with a 1.2–threefold increase in the development of VTE [18, 19].

Even though many known DVT risk factors were present in our sample, they proved to be statistically insignificant. However, the incidence rate of DVT remained relatively high (24.8%). Consequently, we conducted logistic regression analysis to determine the presence of other risk factors, particularly metaphyseal trauma, which may increase the likelihood of DVT incidence. Within this analysis, we included the extent of metaphyseal trauma as one of the risk factors and compared it to the other existing risk factors. Both TA and HA/ORIF represent the extent of metaphyseal trauma that occurs in the hip and knee major orthopedic surgery. Here we found that the largest metaphyseal trauma in TA has a sixfold increase of developing DVT compared to HA and ORIF. Interestingly, a more recent study from Fu et al. with a larger sample found a similar finding in a femoral neck fracture population, where patients who underwent THR and HA have a higher incidence of DVT [20]. In the relationship between metaphyseal trauma area and DVT incidence, the isolated upper extremity fracture also is not associated with significant increase in DVT [21]. This finding adds to the literature by specifically identifying metaphyseal trauma as a risk factor in major orthopedic surgery and understanding the risk associated with different types of orthopedic trauma.

Excessive bone cutting in major orthopedic surgery has been widely known to cause complications, i.e., alignment and instability issues that could lead to implant loosening in the future [22]. The increasing risk of DVT during hip and knee surgery most likely occurs due to the injury to bone and soft tissues, which causes extensive endothelial damage. This stimulates the release of tissue factor, initiates local activation of the clotting cascade, and increases the activity of plasminogen activator inhibitor. Activation of coagulation results in the generation of excessive amounts of thrombin, which then leads to thrombus formation and platelet activation [23]. Minimizing metaphyseal bone trauma may play a crucial role in reducing DVT incidence by optimizing venous flow, preserving endothelial integrity, and reducing hypercoagulability.

The most significant finding of this study was that metaphyseal trauma is an independent risk factor for DVT, particularly in TKA and THA. Therefore, the necessity of fine surgical technique, gentle handling, adjustment in broaching and reaming technique, and correct sizing in major orthopedic surgery is now a critical part to mitigate the risk of DVT.

Clinical practice guidelines have been proposed to assist healthcare providers in offering prophylactic measures against the risk of DVT in major orthopedic surgery. At the same time, there is a lack of published evidence to give specific guidance for selection of the optimum thromboprophylaxis after surgery for fractures around the knee [24]. From our findings, the extent of metaphyseal trauma arises as a critical factor that warrants updating these guidelines to align with current practices.

## Limitations

The sample size is limited, and the extent of metaphyseal damage was estimated indirectly based on implant surface measurements, as direct bone traumatization measurements were impractical intraoperatively. Another limitation to consider is that our study only focuses on the extent of metaphyseal trauma without a direct comparison to diaphyseal trauma. We also assume that the amount of soft tissue injury in all three groups is comparable. Due to the consecutive nature of the study, the number of patients in each group is unequal with also an unequal distribution of the risk factors.

## Future directions

Future studies with a larger sample and more accurate measurement of bone traumatization will provide valuable insight into the role of metaphyseal bone trauma in DVT. Studies focusing on selective use of thromboprophylaxis based on the extent metaphyseal trauma, classified according to the type of orthopedic surgeries, could contribute to reach a thromboprophylaxis management consensus in clinical practice.

## Conclusions

Metaphyseal bone trauma is an independent risk factor for deep vein thrombosis, especially in total knee and total hip arthroplasty.

### Supplementary Information

Below is the link to the electronic supplementary material.

Supplementary file1 (SAV 38 kb)

Supplementary file2 (DOCX 51 kb)

Supplementary file3 (DOCX 51 kb)

Supplementary file4 (DOCX 48 kb)

Supplementary file5 (DOCX 49 kb)

Supplementary file6 (DOCX 52 kb)

## Abbreviations

AAOS: American Academy of Orthopaedic Surgeon
ACCP: American College of Chest Physicians
AMP: Austin Moore Hip Prosthesis
BHP: Bipolar hip prosthesis
BMI: Body mass index
CBP: Condylar blade plate
CKD: Chronic kidney disease
CS: Cannulated screws
D-1: One day before surgery
D + 7: 7 Days after surgery
DHS: Dynamic hip screws
DVT: Deep vein thrombosis
FF: Proximal and distal femur fixation
HA: Hemiarthroplasty group
HDL: High-density lipoprotein
HHA: Hip hemiarthroplasty
LDL: Low-density lipoprotein
Med: Median
Min: Minimum
Max: Maximum
N: Normal
ORIF: Open reduction and internal fixation
OR: Odds ratio
PFLP: Proximal femur locking plate
Sig: Significances
TA: Total arthroplasty group
TKA: Total knee arthroplasty
VTE: Venous thromboembolism
