# Supplementary material for: Metaphyseal trauma of the lower extremities in major orthopedic surgery as an independent risk factor for deep vein thrombosis
Source: Eur J Orthop Surg Traumatol. 2024 May 23;34(5):2797–803. doi: 10.1007/s00590-024-03960-4 (PMC11291529; doi:10.1007/s00590-024-03960-4)
Supplement: Supplementary file 2 — Supplementary file2 (DOCX 51 kb) [file 590_2024_3960_MOESM2_ESM.docx]

CROSSTABS
 /TABLES=DVTcross BY kriteriausia kriteriaBMI kriteriaFibrinogenH1 hipertensi DM RfibrinogenH1
 stroke talasemia Rlamaoperasi Rtotalperdarahan masalah_jantung Merokok jeniskelamin malgnancy
 kriteriH7fibrinogen RkriteriaDdimerH1 kriteriaH7d_dimer RkriteriaDdimerH7 kriteriHDL kriteriaLDL
 kriteriatrigliserida kriteriatotcolestr
 /FORMAT=AVALUE TABLES
 /STATISTICS=CHISQ BTAU CTAU RISK
 /CELLS=COUNT EXPECTED
 /COUNT ROUND CELL.

**Crosstabs**

| **Notes** |  |  |
| --- | --- | --- |
| Output Created |  | 19-JAN-2024 10:17:25 |
| Comments |  |  |
| Input | Data | C:\Users\nicho\Documents\ortho\101RR_artikel 2 R-2 revisi dr Iwan[1].sav |
|  | Active Dataset | DataSet1 |
|  | Filter | <none> |
|  | Weight | <none> |
|  | Split File | <none> |
|  | N of Rows in Working Data File | 32 |
| Missing Value Handling | Definition of Missing | User-defined missing values are treated as missing. |
|  | Cases Used | Statistics for each table are based on all the cases with valid data in the specified range(s) for all variables in each table. |
| Syntax |  | CROSSTABS /TABLES=DVTcross BY kriteriausia kriteriaBMI kriteriaFibrinogenH1 hipertensi DM RfibrinogenH1 stroke talasemia Rlamaoperasi Rtotalperdarahan masalah_jantung Merokok jeniskelamin malgnancy kriteriH7fibrinogen RkriteriaDdimerH1 kriteriaH7d_dimer RkriteriaDdimerH7 kriteriHDL kriteriaLDL kriteriatrigliserida kriteriatotcolestr /FORMAT=AVALUE TABLES /STATISTICS=CHISQ BTAU CTAU RISK /CELLS=COUNT EXPECTED /COUNT ROUND CELL. |
| Resources | Processor Time | 00:00:00,00 |
|  | Elapsed Time | 00:00:00,05 |
|  | Dimensions Requested | 2 |
|  | Cells Available | 349496 |

| **Case Processing Summary** |  |  |  |  |  |  |
| --- | --- | --- | --- | --- | --- | --- |
|  | Cases |  |  |  |  |  |
|  | Valid |  | Missing |  | Total |  |
|  | N | Percent | N | Percent | N | Percent |
| DVT responden * kriteria usia | 32 | 100.0% | 0 | 0.0% | 32 | 100.0% |
| DVT responden * Kriteria BMi | 32 | 100.0% | 0 | 0.0% | 32 | 100.0% |
| DVT responden * kriteria Fibrinogen H1 | 32 | 100.0% | 0 | 0.0% | 32 | 100.0% |
| DVT responden * Riwayat hipertensi | 32 | 100.0% | 0 | 0.0% | 32 | 100.0% |
| DVT responden * Riwayat DM | 32 | 100.0% | 0 | 0.0% | 32 | 100.0% |
| DVT responden * Rfibrinogen H1 | 32 | 100.0% | 0 | 0.0% | 32 | 100.0% |
| DVT responden * Riwatat stroke | 32 | 100.0% | 0 | 0.0% | 32 | 100.0% |
| DVT responden * Riwayat talasemia pasien | 32 | 100.0% | 0 | 0.0% | 32 | 100.0% |
| DVT responden * R lama operasi | 32 | 100.0% | 0 | 0.0% | 32 | 100.0% |
| DVT responden * R totalperdarahn | 32 | 100.0% | 0 | 0.0% | 32 | 100.0% |
| DVT responden * Riwayat jantung | 32 | 100.0% | 0 | 0.0% | 32 | 100.0% |
| DVT responden * Riwayat merokok | 32 | 100.0% | 0 | 0.0% | 32 | 100.0% |
| DVT responden * jenis kelamin responden | 32 | 100.0% | 0 | 0.0% | 32 | 100.0% |
| DVT responden * Malignancy | 32 | 100.0% | 0 | 0.0% | 32 | 100.0% |
| DVT responden * kriteriaH7 fibrinogen | 32 | 100.0% | 0 | 0.0% | 32 | 100.0% |
| DVT responden * RkriteriaDdimer H1 | 32 | 100.0% | 0 | 0.0% | 32 | 100.0% |
| DVT responden * kriteria H7 D-dimer | 32 | 100.0% | 0 | 0.0% | 32 | 100.0% |
| DVT responden * RKriteria DdimerH7 | 32 | 100.0% | 0 | 0.0% | 32 | 100.0% |
| DVT responden * kriteria HDL | 32 | 100.0% | 0 | 0.0% | 32 | 100.0% |
| DVT responden * kriteria LDL | 32 | 100.0% | 0 | 0.0% | 32 | 100.0% |
| DVT responden * kriteria trigliserida | 32 | 100.0% | 0 | 0.0% | 32 | 100.0% |
| DVT responden * kriteria tot.col | 32 | 100.0% | 0 | 0.0% | 32 | 100.0% |

**DVT responden * kriteria usia**

| **Crosstab** |  |  |  |  |  |
| --- | --- | --- | --- | --- | --- |
|  |  |  | kriteria usia |  | Total |
|  |  |  | >=71 | 50-70 |  |
| DVT responden | positif | Count | 4 | 1 | 5 |
|  |  | Expected Count | 3.9 | 1.1 | 5.0 |
|  | negatif | Count | 21 | 6 | 27 |
|  |  | Expected Count | 21.1 | 5.9 | 27.0 |
| Total |  | Count | 25 | 7 | 32 |
|  |  | Expected Count | 25.0 | 7.0 | 32.0 |

| **Chi-Square Tests** |  |  |  |  |  |
| --- | --- | --- | --- | --- | --- |
|  | Value | df | Asymptotic Significance (2-sided) | Exact Sig. (2-sided) | Exact Sig. (1-sided) |
| Pearson Chi-Square | .012^a^ | 1 | .912 |  |  |
| Continuity Correction^b^ | .000 | 1 | 1.000 |  |  |
| Likelihood Ratio | .012 | 1 | .911 |  |  |
| Fisher's Exact Test |  |  |  | 1.000 | .704 |
| Linear-by-Linear Association | .012 | 1 | .913 |  |  |
| N of Valid Cases | 32 |  |  |  |  |

| a. 2 cells (50,0%) have expected count less than 5. The minimum expected count is 1,09. |  |  |  |  |  |
| --- | --- | --- | --- | --- | --- |
| b. Computed only for a 2x2 table |  |  |  |  |  |

| **Symmetric Measures** |  |  |  |  |  |
| --- | --- | --- | --- | --- | --- |
|  |  | Value | Asymptotic Standard Error^a^ | Approximate T^b^ | Approximate Significance |
| Ordinal by Ordinal | Kendall's tau-b | .020 | .172 | .113 | .910 |
|  | Kendall's tau-c | .012 | .103 | .113 | .910 |
| N of Valid Cases |  | 32 |  |  |  |

| a. Not assuming the null hypothesis. |  |  |  |  |  |
| --- | --- | --- | --- | --- | --- |
| b. Using the asymptotic standard error assuming the null hypothesis. |  |  |  |  |  |

| **Risk Estimate** |  |  |  |
| --- | --- | --- | --- |
|  | Value | 95% Confidence Interval |  |
|  |  | Lower | Upper |
| Odds Ratio for DVT responden (positif / negatif) | 1.143 | .107 | 12.246 |
| For cohort kriteria usia = >=71 | 1.029 | .635 | 1.666 |
| For cohort kriteria usia = 50-70 | .900 | .136 | 5.956 |
| N of Valid Cases | 32 |  |  |

**DVT responden * Kriteria BMi**

| **Crosstab** |  |  |  |  |  |
| --- | --- | --- | --- | --- | --- |
|  |  |  | Kriteria BMi |  | Total |
|  |  |  | overweight >=25.0 | normal <25 |  |
| DVT responden | positif | Count | 0 | 5 | 5 |
|  |  | Expected Count | .6 | 4.4 | 5.0 |
|  | negatif | Count | 4 | 23 | 27 |
|  |  | Expected Count | 3.4 | 23.6 | 27.0 |
| Total |  | Count | 4 | 28 | 32 |
|  |  | Expected Count | 4.0 | 28.0 | 32.0 |

| **Chi-Square Tests** |  |  |  |  |  |
| --- | --- | --- | --- | --- | --- |
|  | Value | df | Asymptotic Significance (2-sided) | Exact Sig. (2-sided) | Exact Sig. (1-sided) |
| Pearson Chi-Square | .847^a^ | 1 | .358 |  |  |
| Continuity Correction^b^ | .034 | 1 | .854 |  |  |
| Likelihood Ratio | 1.461 | 1 | .227 |  |  |
| Fisher's Exact Test |  |  |  | 1.000 | .488 |
| Linear-by-Linear Association | .820 | 1 | .365 |  |  |
| N of Valid Cases | 32 |  |  |  |  |

| a. 3 cells (75,0%) have expected count less than 5. The minimum expected count is ,63. |  |  |  |  |  |
| --- | --- | --- | --- | --- | --- |
| b. Computed only for a 2x2 table |  |  |  |  |  |

| **Symmetric Measures** |  |  |  |  |  |
| --- | --- | --- | --- | --- | --- |
|  |  | Value | Asymptotic Standard Error^a^ | Approximate T^b^ | Approximate Significance |
| Ordinal by Ordinal | Kendall's tau-b | -.163 | .054 | -1.754 | .079 |
|  | Kendall's tau-c | -.078 | .045 | -1.754 | .079 |
| N of Valid Cases |  | 32 |  |  |  |

| a. Not assuming the null hypothesis. |  |  |  |  |  |
| --- | --- | --- | --- | --- | --- |
| b. Using the asymptotic standard error assuming the null hypothesis. |  |  |  |  |  |

| **Risk Estimate** |  |  |  |
| --- | --- | --- | --- |
|  | Value | 95% Confidence Interval |  |
|  |  | Lower | Upper |
| For cohort Kriteria BMi = normal <25 | 1.174 | 1.003 | 1.374 |
| N of Valid Cases | 32 |  |  |

**DVT responden * kriteria Fibrinogen H1**

| **Crosstab** |  |  |  |  |  |
| --- | --- | --- | --- | --- | --- |
|  |  |  | kriteria Fibrinogen H1 |  | Total |
|  |  |  | tinggi >400 | normal <=400 |  |
| DVT responden | positif | Count | 1 | 4 | 5 |
|  |  | Expected Count | 2.2 | 2.8 | 5.0 |
|  | negatif | Count | 13 | 14 | 27 |
|  |  | Expected Count | 11.8 | 15.2 | 27.0 |
| Total |  | Count | 14 | 18 | 32 |
|  |  | Expected Count | 14.0 | 18.0 | 32.0 |

| **Chi-Square Tests** |  |  |  |  |  |
| --- | --- | --- | --- | --- | --- |
|  | Value | df | Asymptotic Significance (2-sided) | Exact Sig. (2-sided) | Exact Sig. (1-sided) |
| Pearson Chi-Square | 1.358^a^ | 1 | .244 |  |  |
| Continuity Correction^b^ | .455 | 1 | .500 |  |  |
| Likelihood Ratio | 1.463 | 1 | .226 |  |  |
| Fisher's Exact Test |  |  |  | .355 | .255 |
| Linear-by-Linear Association | 1.316 | 1 | .251 |  |  |
| N of Valid Cases | 32 |  |  |  |  |

| a. 2 cells (50,0%) have expected count less than 5. The minimum expected count is 2,19. |  |  |  |  |  |
| --- | --- | --- | --- | --- | --- |
| b. Computed only for a 2x2 table |  |  |  |  |  |

| **Symmetric Measures** |  |  |  |  |  |
| --- | --- | --- | --- | --- | --- |
|  |  | Value | Asymptotic Standard Error^a^ | Approximate T^b^ | Approximate Significance |
| Ordinal by Ordinal | Kendall's tau-b | -.206 | .152 | -1.257 | .209 |
|  | Kendall's tau-c | -.148 | .118 | -1.257 | .209 |
| N of Valid Cases |  | 32 |  |  |  |

| a. Not assuming the null hypothesis. |  |  |  |  |  |
| --- | --- | --- | --- | --- | --- |
| b. Using the asymptotic standard error assuming the null hypothesis. |  |  |  |  |  |

| **Risk Estimate** |  |  |  |
| --- | --- | --- | --- |
|  | Value | 95% Confidence Interval |  |
|  |  | Lower | Upper |
| Odds Ratio for DVT responden (positif / negatif) | .269 | .027 | 2.733 |
| For cohort kriteria Fibrinogen H1 = tinggi >400 | .415 | .069 | 2.503 |
| For cohort kriteria Fibrinogen H1 = normal <=400 | 1.543 | .873 | 2.726 |
| N of Valid Cases | 32 |  |  |

**DVT responden * Riwayat hipertensi**

| **Crosstab** |  |  |  |  |  |
| --- | --- | --- | --- | --- | --- |
|  |  |  | Riwayat hipertensi |  | Total |
|  |  |  | ya | tdk |  |
| DVT responden | positif | Count | 4 | 1 | 5 |
|  |  | Expected Count | 2.7 | 2.3 | 5.0 |
|  | negatif | Count | 13 | 14 | 27 |
|  |  | Expected Count | 14.3 | 12.7 | 27.0 |
| Total |  | Count | 17 | 15 | 32 |
|  |  | Expected Count | 17.0 | 15.0 | 32.0 |

| **Chi-Square Tests** |  |  |  |  |  |
| --- | --- | --- | --- | --- | --- |
|  | Value | df | Asymptotic Significance (2-sided) | Exact Sig. (2-sided) | Exact Sig. (1-sided) |
| Pearson Chi-Square | 1.719^a^ | 1 | .190 |  |  |
| Continuity Correction^b^ | .678 | 1 | .410 |  |  |
| Likelihood Ratio | 1.839 | 1 | .175 |  |  |
| Fisher's Exact Test |  |  |  | .338 | .208 |
| Linear-by-Linear Association | 1.665 | 1 | .197 |  |  |
| N of Valid Cases | 32 |  |  |  |  |

| a. 2 cells (50,0%) have expected count less than 5. The minimum expected count is 2,34. |  |  |  |  |  |
| --- | --- | --- | --- | --- | --- |
| b. Computed only for a 2x2 table |  |  |  |  |  |

| **Symmetric Measures** |  |  |  |  |  |
| --- | --- | --- | --- | --- | --- |
|  |  | Value | Asymptotic Standard Error^a^ | Approximate T^b^ | Approximate Significance |
| Ordinal by Ordinal | Kendall's tau-b | .232 | .153 | 1.389 | .165 |
|  | Kendall's tau-c | .168 | .121 | 1.389 | .165 |
| N of Valid Cases |  | 32 |  |  |  |

| a. Not assuming the null hypothesis. |  |  |  |  |  |
| --- | --- | --- | --- | --- | --- |
| b. Using the asymptotic standard error assuming the null hypothesis. |  |  |  |  |  |

| **Risk Estimate** |  |  |  |
| --- | --- | --- | --- |
|  | Value | 95% Confidence Interval |  |
|  |  | Lower | Upper |
| Odds Ratio for DVT responden (positif / negatif) | 4.308 | .424 | 43.733 |
| For cohort Riwayat hipertensi = ya | 1.662 | .923 | 2.990 |
| For cohort Riwayat hipertensi = tdk | .386 | .064 | 2.311 |
| N of Valid Cases | 32 |  |  |

**DVT responden * Riwayat DM**

| **Crosstab** |  |  |  |  |  |
| --- | --- | --- | --- | --- | --- |
|  |  |  | Riwayat DM |  | Total |
|  |  |  | ya | tdk |  |
| DVT responden | positif | Count | 1 | 4 | 5 |
|  |  | Expected Count | 1.1 | 3.9 | 5.0 |
|  | negatif | Count | 6 | 21 | 27 |
|  |  | Expected Count | 5.9 | 21.1 | 27.0 |
| Total |  | Count | 7 | 25 | 32 |
|  |  | Expected Count | 7.0 | 25.0 | 32.0 |

| **Chi-Square Tests** |  |  |  |  |  |
| --- | --- | --- | --- | --- | --- |
|  | Value | df | Asymptotic Significance (2-sided) | Exact Sig. (2-sided) | Exact Sig. (1-sided) |
| Pearson Chi-Square | .012^a^ | 1 | .912 |  |  |
| Continuity Correction^b^ | .000 | 1 | 1.000 |  |  |
| Likelihood Ratio | .012 | 1 | .911 |  |  |
| Fisher's Exact Test |  |  |  | 1.000 | .704 |
| Linear-by-Linear Association | .012 | 1 | .913 |  |  |
| N of Valid Cases | 32 |  |  |  |  |

| a. 2 cells (50,0%) have expected count less than 5. The minimum expected count is 1,09. |  |  |  |  |  |
| --- | --- | --- | --- | --- | --- |
| b. Computed only for a 2x2 table |  |  |  |  |  |

| **Symmetric Measures** |  |  |  |  |  |
| --- | --- | --- | --- | --- | --- |
|  |  | Value | Asymptotic Standard Error^a^ | Approximate T^b^ | Approximate Significance |
| Ordinal by Ordinal | Kendall's tau-b | -.020 | .172 | -.113 | .910 |
|  | Kendall's tau-c | -.012 | .103 | -.113 | .910 |
| N of Valid Cases |  | 32 |  |  |  |

| a. Not assuming the null hypothesis. |  |  |  |  |  |
| --- | --- | --- | --- | --- | --- |
| b. Using the asymptotic standard error assuming the null hypothesis. |  |  |  |  |  |

| **Risk Estimate** |  |  |  |
| --- | --- | --- | --- |
|  | Value | 95% Confidence Interval |  |
|  |  | Lower | Upper |
| Odds Ratio for DVT responden (positif / negatif) | .875 | .082 | 9.376 |
| For cohort Riwayat DM = ya | .900 | .136 | 5.956 |
| For cohort Riwayat DM = tdk | 1.029 | .635 | 1.666 |
| N of Valid Cases | 32 |  |  |

**DVT responden * Rfibrinogen H1**

| **Crosstab** |  |  |  |  |  |
| --- | --- | --- | --- | --- | --- |
|  |  |  | Rfibrinogen H1 |  | Total |
|  |  |  | tinggi | normal |  |
| DVT responden | positif | Count | 1 | 4 | 5 |
|  |  | Expected Count | 2.2 | 2.8 | 5.0 |
|  | negatif | Count | 13 | 14 | 27 |
|  |  | Expected Count | 11.8 | 15.2 | 27.0 |
| Total |  | Count | 14 | 18 | 32 |
|  |  | Expected Count | 14.0 | 18.0 | 32.0 |

| **Chi-Square Tests** |  |  |  |  |  |
| --- | --- | --- | --- | --- | --- |
|  | Value | df | Asymptotic Significance (2-sided) | Exact Sig. (2-sided) | Exact Sig. (1-sided) |
| Pearson Chi-Square | 1.358^a^ | 1 | .244 |  |  |
| Continuity Correction^b^ | .455 | 1 | .500 |  |  |
| Likelihood Ratio | 1.463 | 1 | .226 |  |  |
| Fisher's Exact Test |  |  |  | .355 | .255 |
| Linear-by-Linear Association | 1.316 | 1 | .251 |  |  |
| N of Valid Cases | 32 |  |  |  |  |

| a. 2 cells (50,0%) have expected count less than 5. The minimum expected count is 2,19. |  |  |  |  |  |
| --- | --- | --- | --- | --- | --- |
| b. Computed only for a 2x2 table |  |  |  |  |  |

| **Symmetric Measures** |  |  |  |  |  |
| --- | --- | --- | --- | --- | --- |
|  |  | Value | Asymptotic Standard Error^a^ | Approximate T^b^ | Approximate Significance |
| Ordinal by Ordinal | Kendall's tau-b | -.206 | .152 | -1.257 | .209 |
|  | Kendall's tau-c | -.148 | .118 | -1.257 | .209 |
| N of Valid Cases |  | 32 |  |  |  |

| a. Not assuming the null hypothesis. |  |  |  |  |  |
| --- | --- | --- | --- | --- | --- |
| b. Using the asymptotic standard error assuming the null hypothesis. |  |  |  |  |  |

| **Risk Estimate** |  |  |  |
| --- | --- | --- | --- |
|  | Value | 95% Confidence Interval |  |
|  |  | Lower | Upper |
| Odds Ratio for DVT responden (positif / negatif) | .269 | .027 | 2.733 |
| For cohort Rfibrinogen H1 = tinggi | .415 | .069 | 2.503 |
| For cohort Rfibrinogen H1 = normal | 1.543 | .873 | 2.726 |
| N of Valid Cases | 32 |  |  |

**DVT responden * Riwatat stroke**

| **Crosstab** |  |  |  |  |  |
| --- | --- | --- | --- | --- | --- |
|  |  |  | Riwatat stroke |  | Total |
|  |  |  | ya | tdk |  |
| DVT responden | positif | Count | 1 | 4 | 5 |
|  |  | Expected Count | .9 | 4.1 | 5.0 |
|  | negatif | Count | 5 | 22 | 27 |
|  |  | Expected Count | 5.1 | 21.9 | 27.0 |
| Total |  | Count | 6 | 26 | 32 |
|  |  | Expected Count | 6.0 | 26.0 | 32.0 |

| **Chi-Square Tests** |  |  |  |  |  |
| --- | --- | --- | --- | --- | --- |
|  | Value | df | Asymptotic Significance (2-sided) | Exact Sig. (2-sided) | Exact Sig. (1-sided) |
| Pearson Chi-Square | .006^a^ | 1 | .938 |  |  |
| Continuity Correction^b^ | .000 | 1 | 1.000 |  |  |
| Likelihood Ratio | .006 | 1 | .938 |  |  |
| Fisher's Exact Test |  |  |  | 1.000 | .673 |
| Linear-by-Linear Association | .006 | 1 | .939 |  |  |
| N of Valid Cases | 32 |  |  |  |  |

| a. 2 cells (50,0%) have expected count less than 5. The minimum expected count is ,94. |  |  |  |  |  |
| --- | --- | --- | --- | --- | --- |
| b. Computed only for a 2x2 table |  |  |  |  |  |

| **Symmetric Measures** |  |  |  |  |  |
| --- | --- | --- | --- | --- | --- |
|  |  | Value | Asymptotic Standard Error^a^ | Approximate T^b^ | Approximate Significance |
| Ordinal by Ordinal | Kendall's tau-b | .014 | .180 | .076 | .939 |
|  | Kendall's tau-c | .008 | .102 | .076 | .939 |
| N of Valid Cases |  | 32 |  |  |  |

| a. Not assuming the null hypothesis. |  |  |  |  |  |
| --- | --- | --- | --- | --- | --- |
| b. Using the asymptotic standard error assuming the null hypothesis. |  |  |  |  |  |

| **Risk Estimate** |  |  |  |
| --- | --- | --- | --- |
|  | Value | 95% Confidence Interval |  |
|  |  | Lower | Upper |
| Odds Ratio for DVT responden (positif / negatif) | 1.100 | .100 | 12.087 |
| For cohort Riwatat stroke = ya | 1.080 | .158 | 7.391 |
| For cohort Riwatat stroke = tdk | .982 | .611 | 1.577 |
| N of Valid Cases | 32 |  |  |

**DVT responden * Riwayat talasemia pasien**

| **Crosstab** |  |  |  |  |  |
| --- | --- | --- | --- | --- | --- |
|  |  |  | Riwayat talasemia pasien |  | Total |
|  |  |  | ya | tdk |  |
| DVT responden | positif | Count | 1 | 4 | 5 |
|  |  | Expected Count | .2 | 4.8 | 5.0 |
|  | negatif | Count | 0 | 27 | 27 |
|  |  | Expected Count | .8 | 26.2 | 27.0 |
| Total |  | Count | 1 | 31 | 32 |
|  |  | Expected Count | 1.0 | 31.0 | 32.0 |

| **Chi-Square Tests** |  |  |  |  |  |
| --- | --- | --- | --- | --- | --- |
|  | Value | df | Asymptotic Significance (2-sided) | Exact Sig. (2-sided) | Exact Sig. (1-sided) |
| Pearson Chi-Square | 5.574^a^ | 1 | .018 |  |  |
| Continuity Correction^b^ | .925 | 1 | .336 |  |  |
| Likelihood Ratio | 3.896 | 1 | .048 |  |  |
| Fisher's Exact Test |  |  |  | .156 | .156 |
| Linear-by-Linear Association | 5.400 | 1 | .020 |  |  |
| N of Valid Cases | 32 |  |  |  |  |

| a. 3 cells (75,0%) have expected count less than 5. The minimum expected count is ,16. |  |  |  |  |  |
| --- | --- | --- | --- | --- | --- |
| b. Computed only for a 2x2 table |  |  |  |  |  |

| **Symmetric Measures** |  |  |  |  |  |
| --- | --- | --- | --- | --- | --- |
|  |  | Value | Asymptotic Standard Error^a^ | Approximate T^b^ | Approximate Significance |
| Ordinal by Ordinal | Kendall's tau-b | .417 | .193 | 1.047 | .295 |
|  | Kendall's tau-c | .105 | .101 | 1.047 | .295 |
| N of Valid Cases |  | 32 |  |  |  |

| a. Not assuming the null hypothesis. |  |  |  |  |  |
| --- | --- | --- | --- | --- | --- |
| b. Using the asymptotic standard error assuming the null hypothesis. |  |  |  |  |  |

| **Risk Estimate** |  |  |  |
| --- | --- | --- | --- |
|  | Value | 95% Confidence Interval |  |
|  |  | Lower | Upper |
| For cohort Riwayat talasemia pasien = tdk | .800 | .516 | 1.240 |
| N of Valid Cases | 32 |  |  |

**DVT responden * R lama operasi**

| **Crosstab** |  |  |  |  |  |
| --- | --- | --- | --- | --- | --- |
|  |  |  | R lama operasi |  | Total |
|  |  |  | >= 150 | <150 |  |
| DVT responden | positif | Count | 0 | 5 | 5 |
|  |  | Expected Count | .2 | 4.8 | 5.0 |
|  | negatif | Count | 1 | 26 | 27 |
|  |  | Expected Count | .8 | 26.2 | 27.0 |
| Total |  | Count | 1 | 31 | 32 |
|  |  | Expected Count | 1.0 | 31.0 | 32.0 |

| **Chi-Square Tests** |  |  |  |  |  |
| --- | --- | --- | --- | --- | --- |
|  | Value | df | Asymptotic Significance (2-sided) | Exact Sig. (2-sided) | Exact Sig. (1-sided) |
| Pearson Chi-Square | .191^a^ | 1 | .662 |  |  |
| Continuity Correction^b^ | .000 | 1 | 1.000 |  |  |
| Likelihood Ratio | .346 | 1 | .557 |  |  |
| Fisher's Exact Test |  |  |  | 1.000 | .844 |
| Linear-by-Linear Association | .185 | 1 | .667 |  |  |
| N of Valid Cases | 32 |  |  |  |  |

| a. 3 cells (75,0%) have expected count less than 5. The minimum expected count is ,16. |  |  |  |  |  |
| --- | --- | --- | --- | --- | --- |
| b. Computed only for a 2x2 table |  |  |  |  |  |

| **Symmetric Measures** |  |  |  |  |  |
| --- | --- | --- | --- | --- | --- |
|  |  | Value | Asymptotic Standard Error^a^ | Approximate T^b^ | Approximate Significance |
| Ordinal by Ordinal | Kendall's tau-b | -.077 | .042 | -.964 | .335 |
|  | Kendall's tau-c | -.020 | .020 | -.964 | .335 |
| N of Valid Cases |  | 32 |  |  |  |

| a. Not assuming the null hypothesis. |  |  |  |  |  |
| --- | --- | --- | --- | --- | --- |
| b. Using the asymptotic standard error assuming the null hypothesis. |  |  |  |  |  |

| **Risk Estimate** |  |  |  |
| --- | --- | --- | --- |
|  | Value | 95% Confidence Interval |  |
|  |  | Lower | Upper |
| For cohort R lama operasi = <150 | 1.038 | .964 | 1.118 |
| N of Valid Cases | 32 |  |  |

**DVT responden * R totalperdarahn**

| **Crosstab** |  |  |  |  |  |
| --- | --- | --- | --- | --- | --- |
|  |  |  | R totalperdarahn |  | Total |
|  |  |  | >=500 | <500 |  |
| DVT responden | positif | Count | 3 | 2 | 5 |
|  |  | Expected Count | 2.7 | 2.3 | 5.0 |
|  | negatif | Count | 14 | 13 | 27 |
|  |  | Expected Count | 14.3 | 12.7 | 27.0 |
| Total |  | Count | 17 | 15 | 32 |
|  |  | Expected Count | 17.0 | 15.0 | 32.0 |

| **Chi-Square Tests** |  |  |  |  |  |
| --- | --- | --- | --- | --- | --- |
|  | Value | df | Asymptotic Significance (2-sided) | Exact Sig. (2-sided) | Exact Sig. (1-sided) |
| Pearson Chi-Square | .112^a^ | 1 | .737 |  |  |
| Continuity Correction^b^ | .000 | 1 | 1.000 |  |  |
| Likelihood Ratio | .113 | 1 | .736 |  |  |
| Fisher's Exact Test |  |  |  | 1.000 | .563 |
| Linear-by-Linear Association | .109 | 1 | .741 |  |  |
| N of Valid Cases | 32 |  |  |  |  |

| a. 2 cells (50,0%) have expected count less than 5. The minimum expected count is 2,34. |  |  |  |  |  |
| --- | --- | --- | --- | --- | --- |
| b. Computed only for a 2x2 table |  |  |  |  |  |

| **Symmetric Measures** |  |  |  |  |  |
| --- | --- | --- | --- | --- | --- |
|  |  | Value | Asymptotic Standard Error^a^ | Approximate T^b^ | Approximate Significance |
| Ordinal by Ordinal | Kendall's tau-b | .059 | .174 | .338 | .735 |
|  | Kendall's tau-c | .043 | .127 | .338 | .735 |
| N of Valid Cases |  | 32 |  |  |  |

| a. Not assuming the null hypothesis. |  |  |  |  |  |
| --- | --- | --- | --- | --- | --- |
| b. Using the asymptotic standard error assuming the null hypothesis. |  |  |  |  |  |

| **Risk Estimate** |  |  |  |
| --- | --- | --- | --- |
|  | Value | 95% Confidence Interval |  |
|  |  | Lower | Upper |
| Odds Ratio for DVT responden (positif / negatif) | 1.393 | .200 | 9.711 |
| For cohort R totalperdarahn = >=500 | 1.157 | .519 | 2.582 |
| For cohort R totalperdarahn = <500 | .831 | .265 | 2.605 |
| N of Valid Cases | 32 |  |  |

**DVT responden * Riwayat jantung**

| **Crosstab** |  |  |  |  |  |
| --- | --- | --- | --- | --- | --- |
|  |  |  | Riwayat jantung |  | Total |
|  |  |  | ya | tdk |  |
| DVT responden | positif | Count | 2 | 3 | 5 |
|  |  | Expected Count | .8 | 4.2 | 5.0 |
|  | negatif | Count | 3 | 24 | 27 |
|  |  | Expected Count | 4.2 | 22.8 | 27.0 |
| Total |  | Count | 5 | 27 | 32 |
|  |  | Expected Count | 5.0 | 27.0 | 32.0 |

| **Chi-Square Tests** |  |  |  |  |  |
| --- | --- | --- | --- | --- | --- |
|  | Value | df | Asymptotic Significance (2-sided) | Exact Sig. (2-sided) | Exact Sig. (1-sided) |
| Pearson Chi-Square | 2.671^a^ | 1 | .102 |  |  |
| Continuity Correction^b^ | .929 | 1 | .335 |  |  |
| Likelihood Ratio | 2.170 | 1 | .141 |  |  |
| Fisher's Exact Test |  |  |  | .163 | .163 |
| Linear-by-Linear Association | 2.587 | 1 | .108 |  |  |
| N of Valid Cases | 32 |  |  |  |  |

| a. 3 cells (75,0%) have expected count less than 5. The minimum expected count is ,78. |  |  |  |  |  |
| --- | --- | --- | --- | --- | --- |
| b. Computed only for a 2x2 table |  |  |  |  |  |

| **Symmetric Measures** |  |  |  |  |  |
| --- | --- | --- | --- | --- | --- |
|  |  | Value | Asymptotic Standard Error^a^ | Approximate T^b^ | Approximate Significance |
| Ordinal by Ordinal | Kendall's tau-b | .289 | .220 | 1.170 | .242 |
|  | Kendall's tau-c | .152 | .130 | 1.170 | .242 |
| N of Valid Cases |  | 32 |  |  |  |

| a. Not assuming the null hypothesis. |  |  |  |  |  |
| --- | --- | --- | --- | --- | --- |
| b. Using the asymptotic standard error assuming the null hypothesis. |  |  |  |  |  |

| **Risk Estimate** |  |  |  |
| --- | --- | --- | --- |
|  | Value | 95% Confidence Interval |  |
|  |  | Lower | Upper |
| Odds Ratio for DVT responden (positif / negatif) | 5.333 | .618 | 45.991 |
| For cohort Riwayat jantung = ya | 3.600 | .793 | 16.353 |
| For cohort Riwayat jantung = tdk | .675 | .326 | 1.398 |
| N of Valid Cases | 32 |  |  |

**DVT responden * Riwayat merokok**

| **Crosstab** |  |  |  |  |  |
| --- | --- | --- | --- | --- | --- |
|  |  |  | Riwayat merokok |  | Total |
|  |  |  | ya | tdk |  |
| DVT responden | positif | Count | 0 | 5 | 5 |
|  |  | Expected Count | .5 | 4.5 | 5.0 |
|  | negatif | Count | 3 | 24 | 27 |
|  |  | Expected Count | 2.5 | 24.5 | 27.0 |
| Total |  | Count | 3 | 29 | 32 |
|  |  | Expected Count | 3.0 | 29.0 | 32.0 |

| **Chi-Square Tests** |  |  |  |  |  |
| --- | --- | --- | --- | --- | --- |
|  | Value | df | Asymptotic Significance (2-sided) | Exact Sig. (2-sided) | Exact Sig. (1-sided) |
| Pearson Chi-Square | .613^a^ | 1 | .434 |  |  |
| Continuity Correction^b^ | .000 | 1 | 1.000 |  |  |
| Likelihood Ratio | 1.075 | 1 | .300 |  |  |
| Fisher's Exact Test |  |  |  | 1.000 | .590 |
| Linear-by-Linear Association | .594 | 1 | .441 |  |  |
| N of Valid Cases | 32 |  |  |  |  |

| a. 3 cells (75,0%) have expected count less than 5. The minimum expected count is ,47. |  |  |  |  |  |
| --- | --- | --- | --- | --- | --- |
| b. Computed only for a 2x2 table |  |  |  |  |  |

| **Symmetric Measures** |  |  |  |  |  |
| --- | --- | --- | --- | --- | --- |
|  |  | Value | Asymptotic Standard Error^a^ | Approximate T^b^ | Approximate Significance |
| Ordinal by Ordinal | Kendall's tau-b | -.138 | .050 | -1.565 | .118 |
|  | Kendall's tau-c | -.059 | .037 | -1.565 | .118 |
| N of Valid Cases |  | 32 |  |  |  |

| a. Not assuming the null hypothesis. |  |  |  |  |  |
| --- | --- | --- | --- | --- | --- |
| b. Using the asymptotic standard error assuming the null hypothesis. |  |  |  |  |  |

| **Risk Estimate** |  |  |  |
| --- | --- | --- | --- |
|  | Value | 95% Confidence Interval |  |
|  |  | Lower | Upper |
| For cohort Riwayat merokok = tdk | 1.125 | .985 | 1.285 |
| N of Valid Cases | 32 |  |  |

**DVT responden * jenis kelamin responden**

| **Crosstab** |  |  |  |  |  |
| --- | --- | --- | --- | --- | --- |
|  |  |  | jenis kelamin responden |  | Total |
|  |  |  | P | L |  |
| DVT responden | positif | Count | 5 | 0 | 5 |
|  |  | Expected Count | 4.1 | .9 | 5.0 |
|  | negatif | Count | 21 | 6 | 27 |
|  |  | Expected Count | 21.9 | 5.1 | 27.0 |
| Total |  | Count | 26 | 6 | 32 |
|  |  | Expected Count | 26.0 | 6.0 | 32.0 |

| **Chi-Square Tests** |  |  |  |  |  |
| --- | --- | --- | --- | --- | --- |
|  | Value | df | Asymptotic Significance (2-sided) | Exact Sig. (2-sided) | Exact Sig. (1-sided) |
| Pearson Chi-Square | 1.368^a^ | 1 | .242 |  |  |
| Continuity Correction^b^ | .298 | 1 | .585 |  |  |
| Likelihood Ratio | 2.281 | 1 | .131 |  |  |
| Fisher's Exact Test |  |  |  | .555 | .327 |
| Linear-by-Linear Association | 1.325 | 1 | .250 |  |  |
| N of Valid Cases | 32 |  |  |  |  |

| a. 2 cells (50,0%) have expected count less than 5. The minimum expected count is ,94. |  |  |  |  |  |
| --- | --- | --- | --- | --- | --- |
| b. Computed only for a 2x2 table |  |  |  |  |  |

| **Symmetric Measures** |  |  |  |  |  |
| --- | --- | --- | --- | --- | --- |
|  |  | Value | Asymptotic Standard Error^a^ | Approximate T^b^ | Approximate Significance |
| Ordinal by Ordinal | Kendall's tau-b | .207 | .061 | 2.034 | .042 |
|  | Kendall's tau-c | .117 | .058 | 2.034 | .042 |
| N of Valid Cases |  | 32 |  |  |  |

| a. Not assuming the null hypothesis. |  |  |  |  |  |
| --- | --- | --- | --- | --- | --- |
| b. Using the asymptotic standard error assuming the null hypothesis. |  |  |  |  |  |

| **Risk Estimate** |  |  |  |
| --- | --- | --- | --- |
|  | Value | 95% Confidence Interval |  |
|  |  | Lower | Upper |
| For cohort jenis kelamin responden = P | 1.286 | 1.051 | 1.573 |
| N of Valid Cases | 32 |  |  |

**DVT responden * Malignancy**

| **Crosstab** |  |  |  |  |  |
| --- | --- | --- | --- | --- | --- |
|  |  |  | Malignancy |  | Total |
|  |  |  | ya | tdk |  |
| DVT responden | positif | Count | 1 | 4 | 5 |
|  |  | Expected Count | .3 | 4.7 | 5.0 |
|  | negatif | Count | 1 | 26 | 27 |
|  |  | Expected Count | 1.7 | 25.3 | 27.0 |
| Total |  | Count | 2 | 30 | 32 |
|  |  | Expected Count | 2.0 | 30.0 | 32.0 |

| **Chi-Square Tests** |  |  |  |  |  |
| --- | --- | --- | --- | --- | --- |
|  | Value | df | Asymptotic Significance (2-sided) | Exact Sig. (2-sided) | Exact Sig. (1-sided) |
| Pearson Chi-Square | 1.912^a^ | 1 | .167 |  |  |
| Continuity Correction^b^ | .142 | 1 | .706 |  |  |
| Likelihood Ratio | 1.404 | 1 | .236 |  |  |
| Fisher's Exact Test |  |  |  | .292 | .292 |
| Linear-by-Linear Association | 1.852 | 1 | .174 |  |  |
| N of Valid Cases | 32 |  |  |  |  |

| a. 3 cells (75,0%) have expected count less than 5. The minimum expected count is ,31. |  |  |  |  |  |
| --- | --- | --- | --- | --- | --- |
| b. Computed only for a 2x2 table |  |  |  |  |  |

| **Symmetric Measures** |  |  |  |  |  |
| --- | --- | --- | --- | --- | --- |
|  |  | Value | Asymptotic Standard Error^a^ | Approximate T^b^ | Approximate Significance |
| Ordinal by Ordinal | Kendall's tau-b | .244 | .245 | .855 | .392 |
|  | Kendall's tau-c | .086 | .100 | .855 | .392 |
| N of Valid Cases |  | 32 |  |  |  |

| a. Not assuming the null hypothesis. |  |  |  |  |  |
| --- | --- | --- | --- | --- | --- |
| b. Using the asymptotic standard error assuming the null hypothesis. |  |  |  |  |  |

| **Risk Estimate** |  |  |  |
| --- | --- | --- | --- |
|  | Value | 95% Confidence Interval |  |
|  |  | Lower | Upper |
| Odds Ratio for DVT responden (positif / negatif) | 6.500 | .335 | 126.061 |
| For cohort Malignancy = ya | 5.400 | .400 | 72.877 |
| For cohort Malignancy = tdk | .831 | .533 | 1.296 |
| N of Valid Cases | 32 |  |  |

**DVT responden * kriteriaH7 fibrinogen**

| **Crosstab** |  |  |  |  |  |
| --- | --- | --- | --- | --- | --- |
|  |  |  | kriteriaH7 fibrinogen |  | Total |
|  |  |  | tinggi > 400 | normal < = 400 |  |
| DVT responden | positif | Count | 3 | 2 | 5 |
|  |  | Expected Count | 3.0 | 2.0 | 5.0 |
|  | negatif | Count | 16 | 11 | 27 |
|  |  | Expected Count | 16.0 | 11.0 | 27.0 |
| Total |  | Count | 19 | 13 | 32 |
|  |  | Expected Count | 19.0 | 13.0 | 32.0 |

| **Chi-Square Tests** |  |  |  |  |  |
| --- | --- | --- | --- | --- | --- |
|  | Value | df | Asymptotic Significance (2-sided) | Exact Sig. (2-sided) | Exact Sig. (1-sided) |
| Pearson Chi-Square | .001^a^ | 1 | .975 |  |  |
| Continuity Correction^b^ | .000 | 1 | 1.000 |  |  |
| Likelihood Ratio | .001 | 1 | .975 |  |  |
| Fisher's Exact Test |  |  |  | 1.000 | .683 |
| Linear-by-Linear Association | .001 | 1 | .976 |  |  |
| N of Valid Cases | 32 |  |  |  |  |

| a. 2 cells (50,0%) have expected count less than 5. The minimum expected count is 2,03. |  |  |  |  |  |
| --- | --- | --- | --- | --- | --- |
| b. Computed only for a 2x2 table |  |  |  |  |  |

| **Symmetric Measures** |  |  |  |  |  |
| --- | --- | --- | --- | --- | --- |
|  |  | Value | Asymptotic Standard Error^a^ | Approximate T^b^ | Approximate Significance |
| Ordinal by Ordinal | Kendall's tau-b | .005 | .176 | .031 | .975 |
|  | Kendall's tau-c | .004 | .126 | .031 | .975 |
| N of Valid Cases |  | 32 |  |  |  |

| a. Not assuming the null hypothesis. |  |  |  |  |  |
| --- | --- | --- | --- | --- | --- |
| b. Using the asymptotic standard error assuming the null hypothesis. |  |  |  |  |  |

| **Risk Estimate** |  |  |  |
| --- | --- | --- | --- |
|  | Value | 95% Confidence Interval |  |
|  |  | Lower | Upper |
| Odds Ratio for DVT responden (positif / negatif) | 1.031 | .147 | 7.226 |
| For cohort kriteriaH7 fibrinogen = tinggi > 400 | 1.013 | .464 | 2.211 |
| For cohort kriteriaH7 fibrinogen = normal < = 400 | .982 | .306 | 3.151 |
| N of Valid Cases | 32 |  |  |

**DVT responden * RkriteriaDdimer H1**

| **Crosstab** |  |  |  |  |  |
| --- | --- | --- | --- | --- | --- |
|  |  |  | RkriteriaDdimer H1 |  | Total |
|  |  |  | tinggi | normal |  |
| DVT responden | positif | Count | 3 | 2 | 5 |
|  |  | Expected Count | 2.8 | 2.2 | 5.0 |
|  | negatif | Count | 15 | 12 | 27 |
|  |  | Expected Count | 15.2 | 11.8 | 27.0 |
| Total |  | Count | 18 | 14 | 32 |
|  |  | Expected Count | 18.0 | 14.0 | 32.0 |

| **Chi-Square Tests** |  |  |  |  |  |
| --- | --- | --- | --- | --- | --- |
|  | Value | df | Asymptotic Significance (2-sided) | Exact Sig. (2-sided) | Exact Sig. (1-sided) |
| Pearson Chi-Square | .034^a^ | 1 | .854 |  |  |
| Continuity Correction^b^ | .000 | 1 | 1.000 |  |  |
| Likelihood Ratio | .034 | 1 | .854 |  |  |
| Fisher's Exact Test |  |  |  | 1.000 | .624 |
| Linear-by-Linear Association | .033 | 1 | .856 |  |  |
| N of Valid Cases | 32 |  |  |  |  |

| a. 2 cells (50,0%) have expected count less than 5. The minimum expected count is 2,19. |  |  |  |  |  |
| --- | --- | --- | --- | --- | --- |
| b. Computed only for a 2x2 table |  |  |  |  |  |

| **Symmetric Measures** |  |  |  |  |  |
| --- | --- | --- | --- | --- | --- |
|  |  | Value | Asymptotic Standard Error^a^ | Approximate T^b^ | Approximate Significance |
| Ordinal by Ordinal | Kendall's tau-b | .033 | .175 | .186 | .853 |
|  | Kendall's tau-c | .023 | .126 | .186 | .853 |
| N of Valid Cases |  | 32 |  |  |  |

| a. Not assuming the null hypothesis. |  |  |  |  |  |
| --- | --- | --- | --- | --- | --- |
| b. Using the asymptotic standard error assuming the null hypothesis. |  |  |  |  |  |

| **Risk Estimate** |  |  |  |
| --- | --- | --- | --- |
|  | Value | 95% Confidence Interval |  |
|  |  | Lower | Upper |
| Odds Ratio for DVT responden (positif / negatif) | 1.200 | .172 | 8.380 |
| For cohort RkriteriaDdimer H1 = tinggi | 1.080 | .490 | 2.383 |
| For cohort RkriteriaDdimer H1 = normal | .900 | .284 | 2.852 |
| N of Valid Cases | 32 |  |  |

**DVT responden * kriteria H7 D-dimer**

| **Crosstab** |  |  |  |  |  |
| --- | --- | --- | --- | --- | --- |
|  |  |  | kriteria H7 D-dimer |  | Total |
|  |  |  | tinggi > =500 | normal < 500 |  |
| DVT responden | positif | Count | 4 | 1 | 5 |
|  |  | Expected Count | 2.7 | 2.3 | 5.0 |
|  | negatif | Count | 13 | 14 | 27 |
|  |  | Expected Count | 14.3 | 12.7 | 27.0 |
| Total |  | Count | 17 | 15 | 32 |
|  |  | Expected Count | 17.0 | 15.0 | 32.0 |

| **Chi-Square Tests** |  |  |  |  |  |
| --- | --- | --- | --- | --- | --- |
|  | Value | df | Asymptotic Significance (2-sided) | Exact Sig. (2-sided) | Exact Sig. (1-sided) |
| Pearson Chi-Square | 1.719^a^ | 1 | .190 |  |  |
| Continuity Correction^b^ | .678 | 1 | .410 |  |  |
| Likelihood Ratio | 1.839 | 1 | .175 |  |  |
| Fisher's Exact Test |  |  |  | .338 | .208 |
| Linear-by-Linear Association | 1.665 | 1 | .197 |  |  |
| N of Valid Cases | 32 |  |  |  |  |

| a. 2 cells (50,0%) have expected count less than 5. The minimum expected count is 2,34. |  |  |  |  |  |
| --- | --- | --- | --- | --- | --- |
| b. Computed only for a 2x2 table |  |  |  |  |  |

| **Symmetric Measures** |  |  |  |  |  |
| --- | --- | --- | --- | --- | --- |
|  |  | Value | Asymptotic Standard Error^a^ | Approximate T^b^ | Approximate Significance |
| Ordinal by Ordinal | Kendall's tau-b | .232 | .153 | 1.389 | .165 |
|  | Kendall's tau-c | .168 | .121 | 1.389 | .165 |
| N of Valid Cases |  | 32 |  |  |  |

| a. Not assuming the null hypothesis. |  |  |  |  |  |
| --- | --- | --- | --- | --- | --- |
| b. Using the asymptotic standard error assuming the null hypothesis. |  |  |  |  |  |

| **Risk Estimate** |  |  |  |
| --- | --- | --- | --- |
|  | Value | 95% Confidence Interval |  |
|  |  | Lower | Upper |
| Odds Ratio for DVT responden (positif / negatif) | 4.308 | .424 | 43.733 |
| For cohort kriteria H7 D-dimer = tinggi > =500 | 1.662 | .923 | 2.990 |
| For cohort kriteria H7 D-dimer = normal < 500 | .386 | .064 | 2.311 |
| N of Valid Cases | 32 |  |  |

**DVT responden * RKriteria DdimerH7**

| **Crosstab** |  |  |  |  |  |
| --- | --- | --- | --- | --- | --- |
|  |  |  | RKriteria DdimerH7 |  | Total |
|  |  |  | tinggi | normal |  |
| DVT responden | positif | Count | 4 | 1 | 5 |
|  |  | Expected Count | 2.7 | 2.3 | 5.0 |
|  | negatif | Count | 13 | 14 | 27 |
|  |  | Expected Count | 14.3 | 12.7 | 27.0 |
| Total |  | Count | 17 | 15 | 32 |
|  |  | Expected Count | 17.0 | 15.0 | 32.0 |

| **Chi-Square Tests** |  |  |  |  |  |
| --- | --- | --- | --- | --- | --- |
|  | Value | df | Asymptotic Significance (2-sided) | Exact Sig. (2-sided) | Exact Sig. (1-sided) |
| Pearson Chi-Square | 1.719^a^ | 1 | .190 |  |  |
| Continuity Correction^b^ | .678 | 1 | .410 |  |  |
| Likelihood Ratio | 1.839 | 1 | .175 |  |  |
| Fisher's Exact Test |  |  |  | .338 | .208 |
| Linear-by-Linear Association | 1.665 | 1 | .197 |  |  |
| N of Valid Cases | 32 |  |  |  |  |

| a. 2 cells (50,0%) have expected count less than 5. The minimum expected count is 2,34. |  |  |  |  |  |
| --- | --- | --- | --- | --- | --- |
| b. Computed only for a 2x2 table |  |  |  |  |  |

| **Symmetric Measures** |  |  |  |  |  |
| --- | --- | --- | --- | --- | --- |
|  |  | Value | Asymptotic Standard Error^a^ | Approximate T^b^ | Approximate Significance |
| Ordinal by Ordinal | Kendall's tau-b | .232 | .153 | 1.389 | .165 |
|  | Kendall's tau-c | .168 | .121 | 1.389 | .165 |
| N of Valid Cases |  | 32 |  |  |  |

| a. Not assuming the null hypothesis. |  |  |  |  |  |
| --- | --- | --- | --- | --- | --- |
| b. Using the asymptotic standard error assuming the null hypothesis. |  |  |  |  |  |

| **Risk Estimate** |  |  |  |
| --- | --- | --- | --- |
|  | Value | 95% Confidence Interval |  |
|  |  | Lower | Upper |
| Odds Ratio for DVT responden (positif / negatif) | 4.308 | .424 | 43.733 |
| For cohort RKriteria DdimerH7 = tinggi | 1.662 | .923 | 2.990 |
| For cohort RKriteria DdimerH7 = normal | .386 | .064 | 2.311 |
| N of Valid Cases | 32 |  |  |

**DVT responden * kriteria HDL**

| **Crosstab** |  |  |  |  |  |
| --- | --- | --- | --- | --- | --- |
|  |  |  | kriteria HDL |  | Total |
|  |  |  | rendah <=40 | tinggi >40 |  |
| DVT responden | positif | Count | 3 | 2 | 5 |
|  |  | Expected Count | 1.4 | 3.6 | 5.0 |
|  | negatif | Count | 6 | 21 | 27 |
|  |  | Expected Count | 7.6 | 19.4 | 27.0 |
| Total |  | Count | 9 | 23 | 32 |
|  |  | Expected Count | 9.0 | 23.0 | 32.0 |

| **Chi-Square Tests** |  |  |  |  |  |
| --- | --- | --- | --- | --- | --- |
|  | Value | df | Asymptotic Significance (2-sided) | Exact Sig. (2-sided) | Exact Sig. (1-sided) |
| Pearson Chi-Square | 2.978^a^ | 1 | .084 |  |  |
| Continuity Correction^b^ | 1.403 | 1 | .236 |  |  |
| Likelihood Ratio | 2.690 | 1 | .101 |  |  |
| Fisher's Exact Test |  |  |  | .121 | .121 |
| Linear-by-Linear Association | 2.885 | 1 | .089 |  |  |
| N of Valid Cases | 32 |  |  |  |  |

| a. 2 cells (50,0%) have expected count less than 5. The minimum expected count is 1,41. |  |  |  |  |  |
| --- | --- | --- | --- | --- | --- |
| b. Computed only for a 2x2 table |  |  |  |  |  |

| **Symmetric Measures** |  |  |  |  |  |
| --- | --- | --- | --- | --- | --- |
|  |  | Value | Asymptotic Standard Error^a^ | Approximate T^b^ | Approximate Significance |
| Ordinal by Ordinal | Kendall's tau-b | .305 | .192 | 1.424 | .154 |
|  | Kendall's tau-c | .199 | .140 | 1.424 | .154 |
| N of Valid Cases |  | 32 |  |  |  |

| a. Not assuming the null hypothesis. |  |  |  |  |  |
| --- | --- | --- | --- | --- | --- |
| b. Using the asymptotic standard error assuming the null hypothesis. |  |  |  |  |  |

| **Risk Estimate** |  |  |  |
| --- | --- | --- | --- |
|  | Value | 95% Confidence Interval |  |
|  |  | Lower | Upper |
| Odds Ratio for DVT responden (positif / negatif) | 5.250 | .706 | 39.029 |
| For cohort kriteria HDL = rendah <=40 | 2.700 | .988 | 7.377 |
| For cohort kriteria HDL = tinggi >40 | .514 | .173 | 1.533 |
| N of Valid Cases | 32 |  |  |

**DVT responden * kriteria LDL**

| **Crosstab** |  |  |  |  |  |
| --- | --- | --- | --- | --- | --- |
|  |  |  | kriteria LDL |  | Total |
|  |  |  | tinggi >= 130 | normal <130 |  |
| DVT responden | positif | Count | 1 | 4 | 5 |
|  |  | Expected Count | 1.4 | 3.6 | 5.0 |
|  | negatif | Count | 8 | 19 | 27 |
|  |  | Expected Count | 7.6 | 19.4 | 27.0 |
| Total |  | Count | 9 | 23 | 32 |
|  |  | Expected Count | 9.0 | 23.0 | 32.0 |

| **Chi-Square Tests** |  |  |  |  |  |
| --- | --- | --- | --- | --- | --- |
|  | Value | df | Asymptotic Significance (2-sided) | Exact Sig. (2-sided) | Exact Sig. (1-sided) |
| Pearson Chi-Square | .194^a^ | 1 | .660 |  |  |
| Continuity Correction^b^ | .000 | 1 | 1.000 |  |  |
| Likelihood Ratio | .205 | 1 | .651 |  |  |
| Fisher's Exact Test |  |  |  | 1.000 | .563 |
| Linear-by-Linear Association | .187 | 1 | .665 |  |  |
| N of Valid Cases | 32 |  |  |  |  |

| a. 2 cells (50,0%) have expected count less than 5. The minimum expected count is 1,41. |  |  |  |  |  |
| --- | --- | --- | --- | --- | --- |
| b. Computed only for a 2x2 table |  |  |  |  |  |

| **Symmetric Measures** |  |  |  |  |  |
| --- | --- | --- | --- | --- | --- |
|  |  | Value | Asymptotic Standard Error^a^ | Approximate T^b^ | Approximate Significance |
| Ordinal by Ordinal | Kendall's tau-b | -.078 | .161 | -.477 | .633 |
|  | Kendall's tau-c | -.051 | .106 | -.477 | .633 |
| N of Valid Cases |  | 32 |  |  |  |

| a. Not assuming the null hypothesis. |  |  |  |  |  |
| --- | --- | --- | --- | --- | --- |
| b. Using the asymptotic standard error assuming the null hypothesis. |  |  |  |  |  |

| **Risk Estimate** |  |  |  |
| --- | --- | --- | --- |
|  | Value | 95% Confidence Interval |  |
|  |  | Lower | Upper |
| Odds Ratio for DVT responden (positif / negatif) | .594 | .057 | 6.175 |
| For cohort kriteria LDL = tinggi >= 130 | .675 | .106 | 4.280 |
| For cohort kriteria LDL = normal <130 | 1.137 | .688 | 1.878 |
| N of Valid Cases | 32 |  |  |

**DVT responden * kriteria trigliserida**

| **Crosstab** |  |  |  |  |  |
| --- | --- | --- | --- | --- | --- |
|  |  |  | kriteria trigliserida |  | Total |
|  |  |  | tinggi >150 | normal <=150 |  |
| DVT responden | positif | Count | 1 | 4 | 5 |
|  |  | Expected Count | .8 | 4.2 | 5.0 |
|  | negatif | Count | 4 | 23 | 27 |
|  |  | Expected Count | 4.2 | 22.8 | 27.0 |
| Total |  | Count | 5 | 27 | 32 |
|  |  | Expected Count | 5.0 | 27.0 | 32.0 |

| **Chi-Square Tests** |  |  |  |  |  |
| --- | --- | --- | --- | --- | --- |
|  | Value | df | Asymptotic Significance (2-sided) | Exact Sig. (2-sided) | Exact Sig. (1-sided) |
| Pearson Chi-Square | .086^a^ | 1 | .769 |  |  |
| Continuity Correction^b^ | .000 | 1 | 1.000 |  |  |
| Likelihood Ratio | .081 | 1 | .775 |  |  |
| Fisher's Exact Test |  |  |  | 1.000 | .599 |
| Linear-by-Linear Association | .083 | 1 | .773 |  |  |
| N of Valid Cases | 32 |  |  |  |  |

| a. 3 cells (75,0%) have expected count less than 5. The minimum expected count is ,78. |  |  |  |  |  |
| --- | --- | --- | --- | --- | --- |
| b. Computed only for a 2x2 table |  |  |  |  |  |

| **Symmetric Measures** |  |  |  |  |  |
| --- | --- | --- | --- | --- | --- |
|  |  | Value | Asymptotic Standard Error^a^ | Approximate T^b^ | Approximate Significance |
| Ordinal by Ordinal | Kendall's tau-b | .052 | .191 | .270 | .787 |
|  | Kendall's tau-c | .027 | .101 | .270 | .787 |
| N of Valid Cases |  | 32 |  |  |  |

| a. Not assuming the null hypothesis. |  |  |  |  |  |
| --- | --- | --- | --- | --- | --- |
| b. Using the asymptotic standard error assuming the null hypothesis. |  |  |  |  |  |

| **Risk Estimate** |  |  |  |
| --- | --- | --- | --- |
|  | Value | 95% Confidence Interval |  |
|  |  | Lower | Upper |
| Odds Ratio for DVT responden (positif / negatif) | 1.438 | .126 | 16.410 |
| For cohort kriteria trigliserida = tinggi >150 | 1.350 | .188 | 9.706 |
| For cohort kriteria trigliserida = normal <=150 | .939 | .590 | 1.496 |
| N of Valid Cases | 32 |  |  |

**DVT responden * kriteria tot.col**

| **Crosstab** |  |  |  |  |  |
| --- | --- | --- | --- | --- | --- |
|  |  |  | kriteria tot.col |  | Total |
|  |  |  | tinggi >200 | normal <=200 |  |
| DVT responden | positif | Count | 1 | 4 | 5 |
|  |  | Expected Count | 1.6 | 3.4 | 5.0 |
|  | negatif | Count | 9 | 18 | 27 |
|  |  | Expected Count | 8.4 | 18.6 | 27.0 |
| Total |  | Count | 10 | 22 | 32 |
|  |  | Expected Count | 10.0 | 22.0 | 32.0 |

| **Chi-Square Tests** |  |  |  |  |  |
| --- | --- | --- | --- | --- | --- |
|  | Value | df | Asymptotic Significance (2-sided) | Exact Sig. (2-sided) | Exact Sig. (1-sided) |
| Pearson Chi-Square | .349^a^ | 1 | .555 |  |  |
| Continuity Correction^b^ | .004 | 1 | .948 |  |  |
| Likelihood Ratio | .374 | 1 | .541 |  |  |
| Fisher's Exact Test |  |  |  | 1.000 | .494 |
| Linear-by-Linear Association | .338 | 1 | .561 |  |  |
| N of Valid Cases | 32 |  |  |  |  |

| a. 2 cells (50,0%) have expected count less than 5. The minimum expected count is 1,56. |  |  |  |  |  |
| --- | --- | --- | --- | --- | --- |
| b. Computed only for a 2x2 table |  |  |  |  |  |

| **Symmetric Measures** |  |  |  |  |  |
| --- | --- | --- | --- | --- | --- |
|  |  | Value | Asymptotic Standard Error^a^ | Approximate T^b^ | Approximate Significance |
| Ordinal by Ordinal | Kendall's tau-b | -.104 | .157 | -.649 | .516 |
|  | Kendall's tau-c | -.070 | .108 | -.649 | .516 |
| N of Valid Cases |  | 32 |  |  |  |

| a. Not assuming the null hypothesis. |  |  |  |  |  |
| --- | --- | --- | --- | --- | --- |
| b. Using the asymptotic standard error assuming the null hypothesis. |  |  |  |  |  |

| **Risk Estimate** |  |  |  |
| --- | --- | --- | --- |
|  | Value | 95% Confidence Interval |  |
|  |  | Lower | Upper |
| Odds Ratio for DVT responden (positif / negatif) | .500 | .049 | 5.154 |
| For cohort kriteria tot.col = tinggi >200 | .600 | .096 | 3.749 |
| For cohort kriteria tot.col = normal <=200 | 1.200 | .718 | 2.004 |
| N of Valid Cases | 32 |  |  |
