# Supplementary material for: Metaphyseal trauma of the lower extremities in major orthopedic surgery as an independent risk factor for deep vein thrombosis
Source: Eur J Orthop Surg Traumatol. 2024 May 23;34(5):2797–803. doi: 10.1007/s00590-024-03960-4 (PMC11291529; doi:10.1007/s00590-024-03960-4)
Supplement: Supplementary file 4 — Supplementary file4 (DOCX 48 kb) [file 590_2024_3960_MOESM4_ESM.docx]

CROSSTABS
 /TABLES=DVTcross BY kriteriausia kriteriaBMI hipertensi DM RfibrinogenH1 stroke talasemia
 Rlamaoperasi Rtotalperdarahan masalah_jantung Merokok jeniskelamin malgnancy kriteriH7fibrinogen
 RkriteriaDdimerH1 kriteriaH7d_dimer RkriteriaDdimerH7 kriteriHDL kriteriaLDL kriteriatrigliserida
 kriteriatotcolestr
 /FORMAT=AVALUE TABLES
 /STATISTICS=CHISQ BTAU CTAU RISK
 /CELLS=COUNT EXPECTED
 /COUNT ROUND CELL.

**Crosstabs**

| **Notes** |  |  |
| --- | --- | --- |
| Output Created |  | 19-JAN-2024 10:15:40 |
| Comments |  |  |
| Input | Data | C:\Users\nicho\Documents\ortho\101RR_artikel 2 R-2 revisi dr Iwan[1].sav |
|  | Active Dataset | DataSet1 |
|  | Filter | <none> |
|  | Weight | <none> |
|  | Split File | <none> |
|  | N of Rows in Working Data File | 26 |
| Missing Value Handling | Definition of Missing | User-defined missing values are treated as missing. |
|  | Cases Used | Statistics for each table are based on all the cases with valid data in the specified range(s) for all variables in each table. |
| Syntax |  | CROSSTABS /TABLES=DVTcross BY kriteriausia kriteriaBMI hipertensi DM RfibrinogenH1 stroke talasemia Rlamaoperasi Rtotalperdarahan masalah_jantung Merokok jeniskelamin malgnancy kriteriH7fibrinogen RkriteriaDdimerH1 kriteriaH7d_dimer RkriteriaDdimerH7 kriteriHDL kriteriaLDL kriteriatrigliserida kriteriatotcolestr /FORMAT=AVALUE TABLES /STATISTICS=CHISQ BTAU CTAU RISK /CELLS=COUNT EXPECTED /COUNT ROUND CELL. |
| Resources | Processor Time | 00:00:00,03 |
|  | Elapsed Time | 00:00:00,05 |
|  | Dimensions Requested | 2 |
|  | Cells Available | 349496 |

| **Case Processing Summary** |  |  |  |  |  |  |
| --- | --- | --- | --- | --- | --- | --- |
|  | Cases |  |  |  |  |  |
|  | Valid |  | Missing |  | Total |  |
|  | N | Percent | N | Percent | N | Percent |
| DVT responden * kriteria usia | 26 | 100.0% | 0 | 0.0% | 26 | 100.0% |
| DVT responden * Kriteria BMi | 26 | 100.0% | 0 | 0.0% | 26 | 100.0% |
| DVT responden * Riwayat hipertensi | 26 | 100.0% | 0 | 0.0% | 26 | 100.0% |
| DVT responden * Riwayat DM | 26 | 100.0% | 0 | 0.0% | 26 | 100.0% |
| DVT responden * Rfibrinogen H1 | 26 | 100.0% | 0 | 0.0% | 26 | 100.0% |
| DVT responden * Riwatat stroke | 26 | 100.0% | 0 | 0.0% | 26 | 100.0% |
| DVT responden * Riwayat talasemia pasien | 26 | 100.0% | 0 | 0.0% | 26 | 100.0% |
| DVT responden * R lama operasi | 26 | 100.0% | 0 | 0.0% | 26 | 100.0% |
| DVT responden * R totalperdarahn | 26 | 100.0% | 0 | 0.0% | 26 | 100.0% |
| DVT responden * Riwayat jantung | 26 | 100.0% | 0 | 0.0% | 26 | 100.0% |
| DVT responden * Riwayat merokok | 26 | 100.0% | 0 | 0.0% | 26 | 100.0% |
| DVT responden * jenis kelamin responden | 26 | 100.0% | 0 | 0.0% | 26 | 100.0% |
| DVT responden * Malignancy | 26 | 100.0% | 0 | 0.0% | 26 | 100.0% |
| DVT responden * kriteriaH7 fibrinogen | 26 | 100.0% | 0 | 0.0% | 26 | 100.0% |
| DVT responden * RkriteriaDdimer H1 | 26 | 100.0% | 0 | 0.0% | 26 | 100.0% |
| DVT responden * kriteria H7 D-dimer | 26 | 100.0% | 0 | 0.0% | 26 | 100.0% |
| DVT responden * RKriteria DdimerH7 | 26 | 100.0% | 0 | 0.0% | 26 | 100.0% |
| DVT responden * kriteria HDL | 26 | 100.0% | 0 | 0.0% | 26 | 100.0% |
| DVT responden * kriteria LDL | 26 | 100.0% | 0 | 0.0% | 26 | 100.0% |
| DVT responden * kriteria trigliserida | 26 | 100.0% | 0 | 0.0% | 26 | 100.0% |
| DVT responden * kriteria tot.col | 26 | 100.0% | 0 | 0.0% | 26 | 100.0% |

**DVT responden * kriteria usia**

| **Crosstab** |  |  |  |  |  |
| --- | --- | --- | --- | --- | --- |
|  |  |  | kriteria usia |  | Total |
|  |  |  | >=71 | 50-70 |  |
| DVT responden | positif | Count | 3 | 1 | 4 |
|  |  | Expected Count | 2.8 | 1.2 | 4.0 |
|  | negatif | Count | 15 | 7 | 22 |
|  |  | Expected Count | 15.2 | 6.8 | 22.0 |
| Total |  | Count | 18 | 8 | 26 |
|  |  | Expected Count | 18.0 | 8.0 | 26.0 |

| **Chi-Square Tests** |  |  |  |  |  |
| --- | --- | --- | --- | --- | --- |
|  | Value | df | Asymptotic Significance (2-sided) | Exact Sig. (2-sided) | Exact Sig. (1-sided) |
| Pearson Chi-Square | .074^a^ | 1 | .786 |  |  |
| Continuity Correction^b^ | .000 | 1 | 1.000 |  |  |
| Likelihood Ratio | .076 | 1 | .782 |  |  |
| Fisher's Exact Test |  |  |  | 1.000 | .641 |
| Linear-by-Linear Association | .071 | 1 | .790 |  |  |
| N of Valid Cases | 26 |  |  |  |  |

| a. 2 cells (50,0%) have expected count less than 5. The minimum expected count is 1,23. |  |  |  |  |  |
| --- | --- | --- | --- | --- | --- |
| b. Computed only for a 2x2 table |  |  |  |  |  |

| **Symmetric Measures** |  |  |  |  |  |
| --- | --- | --- | --- | --- | --- |
|  |  | Value | Asymptotic Standard Error^a^ | Approximate T^b^ | Approximate Significance |
| Ordinal by Ordinal | Kendall's tau-b | .053 | .186 | .285 | .776 |
|  | Kendall's tau-c | .036 | .125 | .285 | .776 |
| N of Valid Cases |  | 26 |  |  |  |

| a. Not assuming the null hypothesis. |  |  |  |  |  |
| --- | --- | --- | --- | --- | --- |
| b. Using the asymptotic standard error assuming the null hypothesis. |  |  |  |  |  |

| **Risk Estimate** |  |  |  |
| --- | --- | --- | --- |
|  | Value | 95% Confidence Interval |  |
|  |  | Lower | Upper |
| Odds Ratio for DVT responden (positif / negatif) | 1.400 | .123 | 15.974 |
| For cohort kriteria usia = >=71 | 1.100 | .584 | 2.073 |
| For cohort kriteria usia = 50-70 | .786 | .129 | 4.773 |
| N of Valid Cases | 26 |  |  |

**DVT responden * Kriteria BMi**

| **Crosstab** |  |  |  |  |  |
| --- | --- | --- | --- | --- | --- |
|  |  |  | Kriteria BMi |  | Total |
|  |  |  | overweight >=25.0 | normal <25 |  |
| DVT responden | positif | Count | 1 | 3 | 4 |
|  |  | Expected Count | .8 | 3.2 | 4.0 |
|  | negatif | Count | 4 | 18 | 22 |
|  |  | Expected Count | 4.2 | 17.8 | 22.0 |
| Total |  | Count | 5 | 21 | 26 |
|  |  | Expected Count | 5.0 | 21.0 | 26.0 |

| **Chi-Square Tests** |  |  |  |  |  |
| --- | --- | --- | --- | --- | --- |
|  | Value | df | Asymptotic Significance (2-sided) | Exact Sig. (2-sided) | Exact Sig. (1-sided) |
| Pearson Chi-Square | .101^a^ | 1 | .750 |  |  |
| Continuity Correction^b^ | .000 | 1 | 1.000 |  |  |
| Likelihood Ratio | .096 | 1 | .757 |  |  |
| Fisher's Exact Test |  |  |  | 1.000 | .600 |
| Linear-by-Linear Association | .097 | 1 | .755 |  |  |
| N of Valid Cases | 26 |  |  |  |  |

| a. 3 cells (75,0%) have expected count less than 5. The minimum expected count is ,77. |  |  |  |  |  |
| --- | --- | --- | --- | --- | --- |
| b. Computed only for a 2x2 table |  |  |  |  |  |

| **Symmetric Measures** |  |  |  |  |  |
| --- | --- | --- | --- | --- | --- |
|  |  | Value | Asymptotic Standard Error^a^ | Approximate T^b^ | Approximate Significance |
| Ordinal by Ordinal | Kendall's tau-b | .062 | .212 | .293 | .770 |
|  | Kendall's tau-c | .036 | .121 | .293 | .770 |
| N of Valid Cases |  | 26 |  |  |  |

| a. Not assuming the null hypothesis. |  |  |  |  |  |
| --- | --- | --- | --- | --- | --- |
| b. Using the asymptotic standard error assuming the null hypothesis. |  |  |  |  |  |

| **Risk Estimate** |  |  |  |
| --- | --- | --- | --- |
|  | Value | 95% Confidence Interval |  |
|  |  | Lower | Upper |
| Odds Ratio for DVT responden (positif / negatif) | 1.500 | .122 | 18.441 |
| For cohort Kriteria BMi = overweight >=25.0 | 1.375 | .203 | 9.331 |
| For cohort Kriteria BMi = normal <25 | .917 | .504 | 1.669 |
| N of Valid Cases | 26 |  |  |

**DVT responden * Riwayat hipertensi**

| **Crosstab** |  |  |  |  |  |
| --- | --- | --- | --- | --- | --- |
|  |  |  | Riwayat hipertensi |  | Total |
|  |  |  | ya | tdk |  |
| DVT responden | positif | Count | 3 | 1 | 4 |
|  |  | Expected Count | 2.5 | 1.5 | 4.0 |
|  | negatif | Count | 13 | 9 | 22 |
|  |  | Expected Count | 13.5 | 8.5 | 22.0 |
| Total |  | Count | 16 | 10 | 26 |
|  |  | Expected Count | 16.0 | 10.0 | 26.0 |

| **Chi-Square Tests** |  |  |  |  |  |
| --- | --- | --- | --- | --- | --- |
|  | Value | df | Asymptotic Significance (2-sided) | Exact Sig. (2-sided) | Exact Sig. (1-sided) |
| Pearson Chi-Square | .362^a^ | 1 | .547 |  |  |
| Continuity Correction^b^ | .002 | 1 | .966 |  |  |
| Likelihood Ratio | .381 | 1 | .537 |  |  |
| Fisher's Exact Test |  |  |  | 1.000 | .496 |
| Linear-by-Linear Association | .348 | 1 | .555 |  |  |
| N of Valid Cases | 26 |  |  |  |  |

| a. 2 cells (50,0%) have expected count less than 5. The minimum expected count is 1,54. |  |  |  |  |  |
| --- | --- | --- | --- | --- | --- |
| b. Computed only for a 2x2 table |  |  |  |  |  |

| **Symmetric Measures** |  |  |  |  |  |
| --- | --- | --- | --- | --- | --- |
|  |  | Value | Asymptotic Standard Error^a^ | Approximate T^b^ | Approximate Significance |
| Ordinal by Ordinal | Kendall's tau-b | .118 | .179 | .642 | .521 |
|  | Kendall's tau-c | .083 | .129 | .642 | .521 |
| N of Valid Cases |  | 26 |  |  |  |

| a. Not assuming the null hypothesis. |  |  |  |  |  |
| --- | --- | --- | --- | --- | --- |
| b. Using the asymptotic standard error assuming the null hypothesis. |  |  |  |  |  |

| **Risk Estimate** |  |  |  |
| --- | --- | --- | --- |
|  | Value | 95% Confidence Interval |  |
|  |  | Lower | Upper |
| Odds Ratio for DVT responden (positif / negatif) | 2.077 | .185 | 23.298 |
| For cohort Riwayat hipertensi = ya | 1.269 | .653 | 2.466 |
| For cohort Riwayat hipertensi = tdk | .611 | .104 | 3.588 |
| N of Valid Cases | 26 |  |  |

**DVT responden * Riwayat DM**

| **Crosstab** |  |  |  |  |  |
| --- | --- | --- | --- | --- | --- |
|  |  |  | Riwayat DM |  | Total |
|  |  |  | ya | tdk |  |
| DVT responden | positif | Count | 2 | 2 | 4 |
|  |  | Expected Count | 1.1 | 2.9 | 4.0 |
|  | negatif | Count | 5 | 17 | 22 |
|  |  | Expected Count | 5.9 | 16.1 | 22.0 |
| Total |  | Count | 7 | 19 | 26 |
|  |  | Expected Count | 7.0 | 19.0 | 26.0 |

| **Chi-Square Tests** |  |  |  |  |  |
| --- | --- | --- | --- | --- | --- |
|  | Value | df | Asymptotic Significance (2-sided) | Exact Sig. (2-sided) | Exact Sig. (1-sided) |
| Pearson Chi-Square | 1.280^a^ | 1 | .258 |  |  |
| Continuity Correction^b^ | .269 | 1 | .604 |  |  |
| Likelihood Ratio | 1.162 | 1 | .281 |  |  |
| Fisher's Exact Test |  |  |  | .287 | .287 |
| Linear-by-Linear Association | 1.230 | 1 | .267 |  |  |
| N of Valid Cases | 26 |  |  |  |  |

| a. 2 cells (50,0%) have expected count less than 5. The minimum expected count is 1,08. |  |  |  |  |  |
| --- | --- | --- | --- | --- | --- |
| b. Computed only for a 2x2 table |  |  |  |  |  |

| **Symmetric Measures** |  |  |  |  |  |
| --- | --- | --- | --- | --- | --- |
|  |  | Value | Asymptotic Standard Error^a^ | Approximate T^b^ | Approximate Significance |
| Ordinal by Ordinal | Kendall's tau-b | .222 | .217 | .958 | .338 |
|  | Kendall's tau-c | .142 | .148 | .958 | .338 |
| N of Valid Cases |  | 26 |  |  |  |

| a. Not assuming the null hypothesis. |  |  |  |  |  |
| --- | --- | --- | --- | --- | --- |
| b. Using the asymptotic standard error assuming the null hypothesis. |  |  |  |  |  |

| **Risk Estimate** |  |  |  |
| --- | --- | --- | --- |
|  | Value | 95% Confidence Interval |  |
|  |  | Lower | Upper |
| Odds Ratio for DVT responden (positif / negatif) | 3.400 | .377 | 30.655 |
| For cohort Riwayat DM = ya | 2.200 | .632 | 7.653 |
| For cohort Riwayat DM = tdk | .647 | .237 | 1.769 |
| N of Valid Cases | 26 |  |  |

**DVT responden * Rfibrinogen H1**

| **Crosstab** |  |  |  |  |  |
| --- | --- | --- | --- | --- | --- |
|  |  |  | Rfibrinogen H1 |  | Total |
|  |  |  | tinggi | normal |  |
| DVT responden | positif | Count | 1 | 3 | 4 |
|  |  | Expected Count | .9 | 3.1 | 4.0 |
|  | negatif | Count | 5 | 17 | 22 |
|  |  | Expected Count | 5.1 | 16.9 | 22.0 |
| Total |  | Count | 6 | 20 | 26 |
|  |  | Expected Count | 6.0 | 20.0 | 26.0 |

| **Chi-Square Tests** |  |  |  |  |  |
| --- | --- | --- | --- | --- | --- |
|  | Value | df | Asymptotic Significance (2-sided) | Exact Sig. (2-sided) | Exact Sig. (1-sided) |
| Pearson Chi-Square | .010^a^ | 1 | .921 |  |  |
| Continuity Correction^b^ | .000 | 1 | 1.000 |  |  |
| Likelihood Ratio | .010 | 1 | .922 |  |  |
| Fisher's Exact Test |  |  |  | 1.000 | .676 |
| Linear-by-Linear Association | .009 | 1 | .922 |  |  |
| N of Valid Cases | 26 |  |  |  |  |

| a. 2 cells (50,0%) have expected count less than 5. The minimum expected count is ,92. |  |  |  |  |  |
| --- | --- | --- | --- | --- | --- |
| b. Computed only for a 2x2 table |  |  |  |  |  |

| **Symmetric Measures** |  |  |  |  |  |
| --- | --- | --- | --- | --- | --- |
|  |  | Value | Asymptotic Standard Error^a^ | Approximate T^b^ | Approximate Significance |
| Ordinal by Ordinal | Kendall's tau-b | .019 | .201 | .097 | .923 |
|  | Kendall's tau-c | .012 | .122 | .097 | .923 |
| N of Valid Cases |  | 26 |  |  |  |

| a. Not assuming the null hypothesis. |  |  |  |  |  |
| --- | --- | --- | --- | --- | --- |
| b. Using the asymptotic standard error assuming the null hypothesis. |  |  |  |  |  |

| **Risk Estimate** |  |  |  |
| --- | --- | --- | --- |
|  | Value | 95% Confidence Interval |  |
|  |  | Lower | Upper |
| Odds Ratio for DVT responden (positif / negatif) | 1.133 | .096 | 13.440 |
| For cohort Rfibrinogen H1 = tinggi | 1.100 | .171 | 7.095 |
| For cohort Rfibrinogen H1 = normal | .971 | .528 | 1.785 |
| N of Valid Cases | 26 |  |  |

**DVT responden * Riwatat stroke**

| **Crosstab** |  |  |  |  |  |
| --- | --- | --- | --- | --- | --- |
|  |  |  | Riwatat stroke |  | Total |
|  |  |  | ya | tdk |  |
| DVT responden | positif | Count | 0 | 4 | 4 |
|  |  | Expected Count | .5 | 3.5 | 4.0 |
|  | negatif | Count | 3 | 19 | 22 |
|  |  | Expected Count | 2.5 | 19.5 | 22.0 |
| Total |  | Count | 3 | 23 | 26 |
|  |  | Expected Count | 3.0 | 23.0 | 26.0 |

| **Chi-Square Tests** |  |  |  |  |  |
| --- | --- | --- | --- | --- | --- |
|  | Value | df | Asymptotic Significance (2-sided) | Exact Sig. (2-sided) | Exact Sig. (1-sided) |
| Pearson Chi-Square | .617^a^ | 1 | .432 |  |  |
| Continuity Correction^b^ | .000 | 1 | 1.000 |  |  |
| Likelihood Ratio | 1.071 | 1 | .301 |  |  |
| Fisher's Exact Test |  |  |  | 1.000 | .592 |
| Linear-by-Linear Association | .593 | 1 | .441 |  |  |
| N of Valid Cases | 26 |  |  |  |  |

| a. 3 cells (75,0%) have expected count less than 5. The minimum expected count is ,46. |  |  |  |  |  |
| --- | --- | --- | --- | --- | --- |
| b. Computed only for a 2x2 table |  |  |  |  |  |

| **Symmetric Measures** |  |  |  |  |  |
| --- | --- | --- | --- | --- | --- |
|  |  | Value | Asymptotic Standard Error^a^ | Approximate T^b^ | Approximate Significance |
| Ordinal by Ordinal | Kendall's tau-b | -.154 | .058 | -1.526 | .127 |
|  | Kendall's tau-c | -.071 | .047 | -1.526 | .127 |
| N of Valid Cases |  | 26 |  |  |  |

| a. Not assuming the null hypothesis. |  |  |  |  |  |
| --- | --- | --- | --- | --- | --- |
| b. Using the asymptotic standard error assuming the null hypothesis. |  |  |  |  |  |

| **Risk Estimate** |  |  |  |
| --- | --- | --- | --- |
|  | Value | 95% Confidence Interval |  |
|  |  | Lower | Upper |
| For cohort Riwatat stroke = tdk | 1.158 | .981 | 1.367 |
| N of Valid Cases | 26 |  |  |

**DVT responden * Riwayat talasemia pasien**

| **Crosstab** |  |  |  |  |  |
| --- | --- | --- | --- | --- | --- |
|  |  |  | Riwayat talasemia pasien |  | Total |
|  |  |  | ya | tdk |  |
| DVT responden | positif | Count | 1 | 3 | 4 |
|  |  | Expected Count | .2 | 3.8 | 4.0 |
|  | negatif | Count | 0 | 22 | 22 |
|  |  | Expected Count | .8 | 21.2 | 22.0 |
| Total |  | Count | 1 | 25 | 26 |
|  |  | Expected Count | 1.0 | 25.0 | 26.0 |

| **Chi-Square Tests** |  |  |  |  |  |
| --- | --- | --- | --- | --- | --- |
|  | Value | df | Asymptotic Significance (2-sided) | Exact Sig. (2-sided) | Exact Sig. (1-sided) |
| Pearson Chi-Square | 5.720^a^ | 1 | .017 |  |  |
| Continuity Correction^b^ | .957 | 1 | .328 |  |  |
| Likelihood Ratio | 3.979 | 1 | .046 |  |  |
| Fisher's Exact Test |  |  |  | .154 | .154 |
| Linear-by-Linear Association | 5.500 | 1 | .019 |  |  |
| N of Valid Cases | 26 |  |  |  |  |

| a. 3 cells (75,0%) have expected count less than 5. The minimum expected count is ,15. |  |  |  |  |  |
| --- | --- | --- | --- | --- | --- |
| b. Computed only for a 2x2 table |  |  |  |  |  |

| **Symmetric Measures** |  |  |  |  |  |
| --- | --- | --- | --- | --- | --- |
|  |  | Value | Asymptotic Standard Error^a^ | Approximate T^b^ | Approximate Significance |
| Ordinal by Ordinal | Kendall's tau-b | .469 | .212 | 1.059 | .290 |
|  | Kendall's tau-c | .130 | .123 | 1.059 | .290 |
| N of Valid Cases |  | 26 |  |  |  |

| a. Not assuming the null hypothesis. |  |  |  |  |  |
| --- | --- | --- | --- | --- | --- |
| b. Using the asymptotic standard error assuming the null hypothesis. |  |  |  |  |  |

| **Risk Estimate** |  |  |  |
| --- | --- | --- | --- |
|  | Value | 95% Confidence Interval |  |
|  |  | Lower | Upper |
| For cohort Riwayat talasemia pasien = tdk | .750 | .426 | 1.321 |
| N of Valid Cases | 26 |  |  |

**DVT responden * R lama operasi**

| **Crosstab** |  |  |  |  |  |
| --- | --- | --- | --- | --- | --- |
|  |  |  | R lama operasi |  | Total |
|  |  |  | >= 150 | <150 |  |
| DVT responden | positif | Count | 0 | 4 | 4 |
|  |  | Expected Count | .3 | 3.7 | 4.0 |
|  | negatif | Count | 2 | 20 | 22 |
|  |  | Expected Count | 1.7 | 20.3 | 22.0 |
| Total |  | Count | 2 | 24 | 26 |
|  |  | Expected Count | 2.0 | 24.0 | 26.0 |

| **Chi-Square Tests** |  |  |  |  |  |
| --- | --- | --- | --- | --- | --- |
|  | Value | df | Asymptotic Significance (2-sided) | Exact Sig. (2-sided) | Exact Sig. (1-sided) |
| Pearson Chi-Square | .394^a^ | 1 | .530 |  |  |
| Continuity Correction^b^ | .000 | 1 | 1.000 |  |  |
| Likelihood Ratio | .698 | 1 | .404 |  |  |
| Fisher's Exact Test |  |  |  | 1.000 | .711 |
| Linear-by-Linear Association | .379 | 1 | .538 |  |  |
| N of Valid Cases | 26 |  |  |  |  |

| a. 3 cells (75,0%) have expected count less than 5. The minimum expected count is ,31. |  |  |  |  |  |
| --- | --- | --- | --- | --- | --- |
| b. Computed only for a 2x2 table |  |  |  |  |  |

| **Symmetric Measures** |  |  |  |  |  |
| --- | --- | --- | --- | --- | --- |
|  |  | Value | Asymptotic Standard Error^a^ | Approximate T^b^ | Approximate Significance |
| Ordinal by Ordinal | Kendall's tau-b | -.123 | .053 | -1.295 | .195 |
|  | Kendall's tau-c | -.047 | .037 | -1.295 | .195 |
| N of Valid Cases |  | 26 |  |  |  |

| a. Not assuming the null hypothesis. |  |  |  |  |  |
| --- | --- | --- | --- | --- | --- |
| b. Using the asymptotic standard error assuming the null hypothesis. |  |  |  |  |  |

| **Risk Estimate** |  |  |  |
| --- | --- | --- | --- |
|  | Value | 95% Confidence Interval |  |
|  |  | Lower | Upper |
| For cohort R lama operasi = <150 | 1.100 | .964 | 1.255 |
| N of Valid Cases | 26 |  |  |

**DVT responden * R totalperdarahn**

| **Crosstab** |  |  |  |  |  |
| --- | --- | --- | --- | --- | --- |
|  |  |  | R totalperdarahn |  | Total |
|  |  |  | >=500 | <500 |  |
| DVT responden | positif | Count | 1 | 3 | 4 |
|  |  | Expected Count | .9 | 3.1 | 4.0 |
|  | negatif | Count | 5 | 17 | 22 |
|  |  | Expected Count | 5.1 | 16.9 | 22.0 |
| Total |  | Count | 6 | 20 | 26 |
|  |  | Expected Count | 6.0 | 20.0 | 26.0 |

| **Chi-Square Tests** |  |  |  |  |  |
| --- | --- | --- | --- | --- | --- |
|  | Value | df | Asymptotic Significance (2-sided) | Exact Sig. (2-sided) | Exact Sig. (1-sided) |
| Pearson Chi-Square | .010^a^ | 1 | .921 |  |  |
| Continuity Correction^b^ | .000 | 1 | 1.000 |  |  |
| Likelihood Ratio | .010 | 1 | .922 |  |  |
| Fisher's Exact Test |  |  |  | 1.000 | .676 |
| Linear-by-Linear Association | .009 | 1 | .922 |  |  |
| N of Valid Cases | 26 |  |  |  |  |

| a. 2 cells (50,0%) have expected count less than 5. The minimum expected count is ,92. |  |  |  |  |  |
| --- | --- | --- | --- | --- | --- |
| b. Computed only for a 2x2 table |  |  |  |  |  |

| **Symmetric Measures** |  |  |  |  |  |
| --- | --- | --- | --- | --- | --- |
|  |  | Value | Asymptotic Standard Error^a^ | Approximate T^b^ | Approximate Significance |
| Ordinal by Ordinal | Kendall's tau-b | .019 | .201 | .097 | .923 |
|  | Kendall's tau-c | .012 | .122 | .097 | .923 |
| N of Valid Cases |  | 26 |  |  |  |

| a. Not assuming the null hypothesis. |  |  |  |  |  |
| --- | --- | --- | --- | --- | --- |
| b. Using the asymptotic standard error assuming the null hypothesis. |  |  |  |  |  |

| **Risk Estimate** |  |  |  |
| --- | --- | --- | --- |
|  | Value | 95% Confidence Interval |  |
|  |  | Lower | Upper |
| Odds Ratio for DVT responden (positif / negatif) | 1.133 | .096 | 13.440 |
| For cohort R totalperdarahn = >=500 | 1.100 | .171 | 7.095 |
| For cohort R totalperdarahn = <500 | .971 | .528 | 1.785 |
| N of Valid Cases | 26 |  |  |

**DVT responden * Riwayat jantung**

| **Crosstab** |  |  |  |  |  |
| --- | --- | --- | --- | --- | --- |
|  |  |  | Riwayat jantung |  | Total |
|  |  |  | ya | tdk |  |
| DVT responden | positif | Count | 1 | 3 | 4 |
|  |  | Expected Count | .8 | 3.2 | 4.0 |
|  | negatif | Count | 4 | 18 | 22 |
|  |  | Expected Count | 4.2 | 17.8 | 22.0 |
| Total |  | Count | 5 | 21 | 26 |
|  |  | Expected Count | 5.0 | 21.0 | 26.0 |

| **Chi-Square Tests** |  |  |  |  |  |
| --- | --- | --- | --- | --- | --- |
|  | Value | df | Asymptotic Significance (2-sided) | Exact Sig. (2-sided) | Exact Sig. (1-sided) |
| Pearson Chi-Square | .101^a^ | 1 | .750 |  |  |
| Continuity Correction^b^ | .000 | 1 | 1.000 |  |  |
| Likelihood Ratio | .096 | 1 | .757 |  |  |
| Fisher's Exact Test |  |  |  | 1.000 | .600 |
| Linear-by-Linear Association | .097 | 1 | .755 |  |  |
| N of Valid Cases | 26 |  |  |  |  |

| a. 3 cells (75,0%) have expected count less than 5. The minimum expected count is ,77. |  |  |  |  |  |
| --- | --- | --- | --- | --- | --- |
| b. Computed only for a 2x2 table |  |  |  |  |  |

| **Symmetric Measures** |  |  |  |  |  |
| --- | --- | --- | --- | --- | --- |
|  |  | Value | Asymptotic Standard Error^a^ | Approximate T^b^ | Approximate Significance |
| Ordinal by Ordinal | Kendall's tau-b | .062 | .212 | .293 | .770 |
|  | Kendall's tau-c | .036 | .121 | .293 | .770 |
| N of Valid Cases |  | 26 |  |  |  |

| a. Not assuming the null hypothesis. |  |  |  |  |  |
| --- | --- | --- | --- | --- | --- |
| b. Using the asymptotic standard error assuming the null hypothesis. |  |  |  |  |  |

| **Risk Estimate** |  |  |  |
| --- | --- | --- | --- |
|  | Value | 95% Confidence Interval |  |
|  |  | Lower | Upper |
| Odds Ratio for DVT responden (positif / negatif) | 1.500 | .122 | 18.441 |
| For cohort Riwayat jantung = ya | 1.375 | .203 | 9.331 |
| For cohort Riwayat jantung = tdk | .917 | .504 | 1.669 |
| N of Valid Cases | 26 |  |  |

**DVT responden * Riwayat merokok**

| **Crosstab** |  |  |  |  |  |
| --- | --- | --- | --- | --- | --- |
|  |  |  | Riwayat merokok |  | Total |
|  |  |  | ya | tdk |  |
| DVT responden | positif | Count | 1 | 3 | 4 |
|  |  | Expected Count | .2 | 3.8 | 4.0 |
|  | negatif | Count | 0 | 22 | 22 |
|  |  | Expected Count | .8 | 21.2 | 22.0 |
| Total |  | Count | 1 | 25 | 26 |
|  |  | Expected Count | 1.0 | 25.0 | 26.0 |

| **Chi-Square Tests** |  |  |  |  |  |
| --- | --- | --- | --- | --- | --- |
|  | Value | df | Asymptotic Significance (2-sided) | Exact Sig. (2-sided) | Exact Sig. (1-sided) |
| Pearson Chi-Square | 5.720^a^ | 1 | .017 |  |  |
| Continuity Correction^b^ | .957 | 1 | .328 |  |  |
| Likelihood Ratio | 3.979 | 1 | .046 |  |  |
| Fisher's Exact Test |  |  |  | .154 | .154 |
| Linear-by-Linear Association | 5.500 | 1 | .019 |  |  |
| N of Valid Cases | 26 |  |  |  |  |

| a. 3 cells (75,0%) have expected count less than 5. The minimum expected count is ,15. |  |  |  |  |  |
| --- | --- | --- | --- | --- | --- |
| b. Computed only for a 2x2 table |  |  |  |  |  |

| **Symmetric Measures** |  |  |  |  |  |
| --- | --- | --- | --- | --- | --- |
|  |  | Value | Asymptotic Standard Error^a^ | Approximate T^b^ | Approximate Significance |
| Ordinal by Ordinal | Kendall's tau-b | .469 | .212 | 1.059 | .290 |
|  | Kendall's tau-c | .130 | .123 | 1.059 | .290 |
| N of Valid Cases |  | 26 |  |  |  |

| a. Not assuming the null hypothesis. |  |  |  |  |  |
| --- | --- | --- | --- | --- | --- |
| b. Using the asymptotic standard error assuming the null hypothesis. |  |  |  |  |  |

| **Risk Estimate** |  |  |  |
| --- | --- | --- | --- |
|  | Value | 95% Confidence Interval |  |
|  |  | Lower | Upper |
| For cohort Riwayat merokok = tdk | .750 | .426 | 1.321 |
| N of Valid Cases | 26 |  |  |

**DVT responden * jenis kelamin responden**

| **Crosstab** |  |  |  |  |  |
| --- | --- | --- | --- | --- | --- |
|  |  |  | jenis kelamin responden |  | Total |
|  |  |  | P | L |  |
| DVT responden | positif | Count | 3 | 1 | 4 |
|  |  | Expected Count | 2.3 | 1.7 | 4.0 |
|  | negatif | Count | 12 | 10 | 22 |
|  |  | Expected Count | 12.7 | 9.3 | 22.0 |
| Total |  | Count | 15 | 11 | 26 |
|  |  | Expected Count | 15.0 | 11.0 | 26.0 |

| **Chi-Square Tests** |  |  |  |  |  |
| --- | --- | --- | --- | --- | --- |
|  | Value | df | Asymptotic Significance (2-sided) | Exact Sig. (2-sided) | Exact Sig. (1-sided) |
| Pearson Chi-Square | .580^a^ | 1 | .446 |  |  |
| Continuity Correction^b^ | .045 | 1 | .832 |  |  |
| Likelihood Ratio | .611 | 1 | .435 |  |  |
| Fisher's Exact Test |  |  |  | .614 | .426 |
| Linear-by-Linear Association | .558 | 1 | .455 |  |  |
| N of Valid Cases | 26 |  |  |  |  |

| a. 2 cells (50,0%) have expected count less than 5. The minimum expected count is 1,69. |  |  |  |  |  |
| --- | --- | --- | --- | --- | --- |
| b. Computed only for a 2x2 table |  |  |  |  |  |

| **Symmetric Measures** |  |  |  |  |  |
| --- | --- | --- | --- | --- | --- |
|  |  | Value | Asymptotic Standard Error^a^ | Approximate T^b^ | Approximate Significance |
| Ordinal by Ordinal | Kendall's tau-b | .149 | .178 | .808 | .419 |
|  | Kendall's tau-c | .107 | .132 | .808 | .419 |
| N of Valid Cases |  | 26 |  |  |  |

| a. Not assuming the null hypothesis. |  |  |  |  |  |
| --- | --- | --- | --- | --- | --- |
| b. Using the asymptotic standard error assuming the null hypothesis. |  |  |  |  |  |

| **Risk Estimate** |  |  |  |
| --- | --- | --- | --- |
|  | Value | 95% Confidence Interval |  |
|  |  | Lower | Upper |
| Odds Ratio for DVT responden (positif / negatif) | 2.500 | .224 | 27.940 |
| For cohort jenis kelamin responden = P | 1.375 | .695 | 2.721 |
| For cohort jenis kelamin responden = L | .550 | .095 | 3.191 |
| N of Valid Cases | 26 |  |  |

**DVT responden * Malignancy**

| **Crosstab** |  |  |  |  |  |
| --- | --- | --- | --- | --- | --- |
|  |  |  | Malignancy |  | Total |
|  |  |  | ya | tdk |  |
| DVT responden | positif | Count | 0 | 4 | 4 |
|  |  | Expected Count | .2 | 3.8 | 4.0 |
|  | negatif | Count | 1 | 21 | 22 |
|  |  | Expected Count | .8 | 21.2 | 22.0 |
| Total |  | Count | 1 | 25 | 26 |
|  |  | Expected Count | 1.0 | 25.0 | 26.0 |

| **Chi-Square Tests** |  |  |  |  |  |
| --- | --- | --- | --- | --- | --- |
|  | Value | df | Asymptotic Significance (2-sided) | Exact Sig. (2-sided) | Exact Sig. (1-sided) |
| Pearson Chi-Square | .189^a^ | 1 | .664 |  |  |
| Continuity Correction^b^ | .000 | 1 | 1.000 |  |  |
| Likelihood Ratio | .341 | 1 | .559 |  |  |
| Fisher's Exact Test |  |  |  | 1.000 | .846 |
| Linear-by-Linear Association | .182 | 1 | .670 |  |  |
| N of Valid Cases | 26 |  |  |  |  |

| a. 3 cells (75,0%) have expected count less than 5. The minimum expected count is ,15. |  |  |  |  |  |
| --- | --- | --- | --- | --- | --- |
| b. Computed only for a 2x2 table |  |  |  |  |  |

| **Symmetric Measures** |  |  |  |  |  |
| --- | --- | --- | --- | --- | --- |
|  |  | Value | Asymptotic Standard Error^a^ | Approximate T^b^ | Approximate Significance |
| Ordinal by Ordinal | Kendall's tau-b | -.085 | .047 | -.955 | .340 |
|  | Kendall's tau-c | -.024 | .025 | -.955 | .340 |
| N of Valid Cases |  | 26 |  |  |  |

| a. Not assuming the null hypothesis. |  |  |  |  |  |
| --- | --- | --- | --- | --- | --- |
| b. Using the asymptotic standard error assuming the null hypothesis. |  |  |  |  |  |

| **Risk Estimate** |  |  |  |
| --- | --- | --- | --- |
|  | Value | 95% Confidence Interval |  |
|  |  | Lower | Upper |
| For cohort Malignancy = tdk | 1.048 | .956 | 1.148 |
| N of Valid Cases | 26 |  |  |

**DVT responden * kriteriaH7 fibrinogen**

| **Crosstab** |  |  |  |  |  |
| --- | --- | --- | --- | --- | --- |
|  |  |  | kriteriaH7 fibrinogen |  | Total |
|  |  |  | tinggi > 400 | normal < = 400 |  |
| DVT responden | positif | Count | 2 | 2 | 4 |
|  |  | Expected Count | 2.6 | 1.4 | 4.0 |
|  | negatif | Count | 15 | 7 | 22 |
|  |  | Expected Count | 14.4 | 7.6 | 22.0 |
| Total |  | Count | 17 | 9 | 26 |
|  |  | Expected Count | 17.0 | 9.0 | 26.0 |

| **Chi-Square Tests** |  |  |  |  |  |
| --- | --- | --- | --- | --- | --- |
|  | Value | df | Asymptotic Significance (2-sided) | Exact Sig. (2-sided) | Exact Sig. (1-sided) |
| Pearson Chi-Square | .494^a^ | 1 | .482 |  |  |
| Continuity Correction^b^ | .017 | 1 | .895 |  |  |
| Likelihood Ratio | .475 | 1 | .491 |  |  |
| Fisher's Exact Test |  |  |  | .591 | .431 |
| Linear-by-Linear Association | .475 | 1 | .491 |  |  |
| N of Valid Cases | 26 |  |  |  |  |

| a. 2 cells (50,0%) have expected count less than 5. The minimum expected count is 1,38. |  |  |  |  |  |
| --- | --- | --- | --- | --- | --- |
| b. Computed only for a 2x2 table |  |  |  |  |  |

| **Symmetric Measures** |  |  |  |  |  |
| --- | --- | --- | --- | --- | --- |
|  |  | Value | Asymptotic Standard Error^a^ | Approximate T^b^ | Approximate Significance |
| Ordinal by Ordinal | Kendall's tau-b | -.138 | .205 | -.655 | .512 |
|  | Kendall's tau-c | -.095 | .145 | -.655 | .512 |
| N of Valid Cases |  | 26 |  |  |  |

| a. Not assuming the null hypothesis. |  |  |  |  |  |
| --- | --- | --- | --- | --- | --- |
| b. Using the asymptotic standard error assuming the null hypothesis. |  |  |  |  |  |

| **Risk Estimate** |  |  |  |
| --- | --- | --- | --- |
|  | Value | 95% Confidence Interval |  |
|  |  | Lower | Upper |
| Odds Ratio for DVT responden (positif / negatif) | .467 | .054 | 4.029 |
| For cohort kriteriaH7 fibrinogen = tinggi > 400 | .733 | .264 | 2.035 |
| For cohort kriteriaH7 fibrinogen = normal < = 400 | 1.571 | .495 | 4.989 |
| N of Valid Cases | 26 |  |  |

**DVT responden * RkriteriaDdimer H1**

| **Crosstab** |  |  |  |  |  |
| --- | --- | --- | --- | --- | --- |
|  |  |  | RkriteriaDdimer H1 |  | Total |
|  |  |  | tinggi | normal |  |
| DVT responden | positif | Count | 3 | 1 | 4 |
|  |  | Expected Count | 3.1 | .9 | 4.0 |
|  | negatif | Count | 17 | 5 | 22 |
|  |  | Expected Count | 16.9 | 5.1 | 22.0 |
| Total |  | Count | 20 | 6 | 26 |
|  |  | Expected Count | 20.0 | 6.0 | 26.0 |

| **Chi-Square Tests** |  |  |  |  |  |
| --- | --- | --- | --- | --- | --- |
|  | Value | df | Asymptotic Significance (2-sided) | Exact Sig. (2-sided) | Exact Sig. (1-sided) |
| Pearson Chi-Square | .010^a^ | 1 | .921 |  |  |
| Continuity Correction^b^ | .000 | 1 | 1.000 |  |  |
| Likelihood Ratio | .010 | 1 | .922 |  |  |
| Fisher's Exact Test |  |  |  | 1.000 | .676 |
| Linear-by-Linear Association | .009 | 1 | .922 |  |  |
| N of Valid Cases | 26 |  |  |  |  |

| a. 2 cells (50,0%) have expected count less than 5. The minimum expected count is ,92. |  |  |  |  |  |
| --- | --- | --- | --- | --- | --- |
| b. Computed only for a 2x2 table |  |  |  |  |  |

| **Symmetric Measures** |  |  |  |  |  |
| --- | --- | --- | --- | --- | --- |
|  |  | Value | Asymptotic Standard Error^a^ | Approximate T^b^ | Approximate Significance |
| Ordinal by Ordinal | Kendall's tau-b | -.019 | .201 | -.097 | .923 |
|  | Kendall's tau-c | -.012 | .122 | -.097 | .923 |
| N of Valid Cases |  | 26 |  |  |  |

| a. Not assuming the null hypothesis. |  |  |  |  |  |
| --- | --- | --- | --- | --- | --- |
| b. Using the asymptotic standard error assuming the null hypothesis. |  |  |  |  |  |

| **Risk Estimate** |  |  |  |
| --- | --- | --- | --- |
|  | Value | 95% Confidence Interval |  |
|  |  | Lower | Upper |
| Odds Ratio for DVT responden (positif / negatif) | .882 | .074 | 10.464 |
| For cohort RkriteriaDdimer H1 = tinggi | .971 | .528 | 1.785 |
| For cohort RkriteriaDdimer H1 = normal | 1.100 | .171 | 7.095 |
| N of Valid Cases | 26 |  |  |

**DVT responden * kriteria H7 D-dimer**

| **Crosstab** |  |  |  |  |  |
| --- | --- | --- | --- | --- | --- |
|  |  |  | kriteria H7 D-dimer |  | Total |
|  |  |  | tinggi > =500 | normal < 500 |  |
| DVT responden | positif | Count | 4 | 0 | 4 |
|  |  | Expected Count | 2.6 | 1.4 | 4.0 |
|  | negatif | Count | 13 | 9 | 22 |
|  |  | Expected Count | 14.4 | 7.6 | 22.0 |
| Total |  | Count | 17 | 9 | 26 |
|  |  | Expected Count | 17.0 | 9.0 | 26.0 |

| **Chi-Square Tests** |  |  |  |  |  |
| --- | --- | --- | --- | --- | --- |
|  | Value | df | Asymptotic Significance (2-sided) | Exact Sig. (2-sided) | Exact Sig. (1-sided) |
| Pearson Chi-Square | 2.503^a^ | 1 | .114 |  |  |
| Continuity Correction^b^ | 1.022 | 1 | .312 |  |  |
| Likelihood Ratio | 3.775 | 1 | .052 |  |  |
| Fisher's Exact Test |  |  |  | .263 | .159 |
| Linear-by-Linear Association | 2.406 | 1 | .121 |  |  |
| N of Valid Cases | 26 |  |  |  |  |

| a. 2 cells (50,0%) have expected count less than 5. The minimum expected count is 1,38. |  |  |  |  |  |
| --- | --- | --- | --- | --- | --- |
| b. Computed only for a 2x2 table |  |  |  |  |  |

| **Symmetric Measures** |  |  |  |  |  |
| --- | --- | --- | --- | --- | --- |
|  |  | Value | Asymptotic Standard Error^a^ | Approximate T^b^ | Approximate Significance |
| Ordinal by Ordinal | Kendall's tau-b | .310 | .089 | 2.197 | .028 |
|  | Kendall's tau-c | .213 | .097 | 2.197 | .028 |
| N of Valid Cases |  | 26 |  |  |  |

| a. Not assuming the null hypothesis. |  |  |  |  |  |
| --- | --- | --- | --- | --- | --- |
| b. Using the asymptotic standard error assuming the null hypothesis. |  |  |  |  |  |

| **Risk Estimate** |  |  |  |
| --- | --- | --- | --- |
|  | Value | 95% Confidence Interval |  |
|  |  | Lower | Upper |
| For cohort kriteria H7 D-dimer = tinggi > =500 | 1.692 | 1.195 | 2.396 |
| N of Valid Cases | 26 |  |  |

**DVT responden * RKriteria DdimerH7**

| **Crosstab** |  |  |  |  |  |
| --- | --- | --- | --- | --- | --- |
|  |  |  | RKriteria DdimerH7 |  | Total |
|  |  |  | tinggi | normal |  |
| DVT responden | positif | Count | 4 | 0 | 4 |
|  |  | Expected Count | 2.8 | 1.2 | 4.0 |
|  | negatif | Count | 14 | 8 | 22 |
|  |  | Expected Count | 15.2 | 6.8 | 22.0 |
| Total |  | Count | 18 | 8 | 26 |
|  |  | Expected Count | 18.0 | 8.0 | 26.0 |

| **Chi-Square Tests** |  |  |  |  |  |
| --- | --- | --- | --- | --- | --- |
|  | Value | df | Asymptotic Significance (2-sided) | Exact Sig. (2-sided) | Exact Sig. (1-sided) |
| Pearson Chi-Square | 2.101^a^ | 1 | .147 |  |  |
| Continuity Correction^b^ | .741 | 1 | .389 |  |  |
| Likelihood Ratio | 3.255 | 1 | .071 |  |  |
| Fisher's Exact Test |  |  |  | .277 | .205 |
| Linear-by-Linear Association | 2.020 | 1 | .155 |  |  |
| N of Valid Cases | 26 |  |  |  |  |

| a. 2 cells (50,0%) have expected count less than 5. The minimum expected count is 1,23. |  |  |  |  |  |
| --- | --- | --- | --- | --- | --- |
| b. Computed only for a 2x2 table |  |  |  |  |  |

| **Symmetric Measures** |  |  |  |  |  |
| --- | --- | --- | --- | --- | --- |
|  |  | Value | Asymptotic Standard Error^a^ | Approximate T^b^ | Approximate Significance |
| Ordinal by Ordinal | Kendall's tau-b | .284 | .083 | 2.126 | .033 |
|  | Kendall's tau-c | .189 | .089 | 2.126 | .033 |
| N of Valid Cases |  | 26 |  |  |  |

| a. Not assuming the null hypothesis. |  |  |  |  |  |
| --- | --- | --- | --- | --- | --- |
| b. Using the asymptotic standard error assuming the null hypothesis. |  |  |  |  |  |

| **Risk Estimate** |  |  |  |
| --- | --- | --- | --- |
|  | Value | 95% Confidence Interval |  |
|  |  | Lower | Upper |
| For cohort RKriteria DdimerH7 = tinggi | 1.571 | 1.146 | 2.155 |
| N of Valid Cases | 26 |  |  |

**DVT responden * kriteria HDL**

| **Crosstab** |  |  |  |  |  |
| --- | --- | --- | --- | --- | --- |
|  |  |  | kriteria HDL |  | Total |
|  |  |  | rendah <=40 | tinggi >40 |  |
| DVT responden | positif | Count | 3 | 1 | 4 |
|  |  | Expected Count | 1.2 | 2.8 | 4.0 |
|  | negatif | Count | 5 | 17 | 22 |
|  |  | Expected Count | 6.8 | 15.2 | 22.0 |
| Total |  | Count | 8 | 18 | 26 |
|  |  | Expected Count | 8.0 | 18.0 | 26.0 |

| **Chi-Square Tests** |  |  |  |  |  |
| --- | --- | --- | --- | --- | --- |
|  | Value | df | Asymptotic Significance (2-sided) | Exact Sig. (2-sided) | Exact Sig. (1-sided) |
| Pearson Chi-Square | 4.342^a^ | 1 | .037 |  |  |
| Continuity Correction^b^ | 2.234 | 1 | .135 |  |  |
| Likelihood Ratio | 4.016 | 1 | .045 |  |  |
| Fisher's Exact Test |  |  |  | .072 | .072 |
| Linear-by-Linear Association | 4.175 | 1 | .041 |  |  |
| N of Valid Cases | 26 |  |  |  |  |

| a. 2 cells (50,0%) have expected count less than 5. The minimum expected count is 1,23. |  |  |  |  |  |
| --- | --- | --- | --- | --- | --- |
| b. Computed only for a 2x2 table |  |  |  |  |  |

| **Symmetric Measures** |  |  |  |  |  |
| --- | --- | --- | --- | --- | --- |
|  |  | Value | Asymptotic Standard Error^a^ | Approximate T^b^ | Approximate Significance |
| Ordinal by Ordinal | Kendall's tau-b | .409 | .195 | 1.709 | .087 |
|  | Kendall's tau-c | .272 | .159 | 1.709 | .087 |
| N of Valid Cases |  | 26 |  |  |  |

| a. Not assuming the null hypothesis. |  |  |  |  |  |
| --- | --- | --- | --- | --- | --- |
| b. Using the asymptotic standard error assuming the null hypothesis. |  |  |  |  |  |

| **Risk Estimate** |  |  |  |
| --- | --- | --- | --- |
|  | Value | 95% Confidence Interval |  |
|  |  | Lower | Upper |
| Odds Ratio for DVT responden (positif / negatif) | 10.200 | .860 | 120.963 |
| For cohort kriteria HDL = rendah <=40 | 3.300 | 1.269 | 8.584 |
| For cohort kriteria HDL = tinggi >40 | .324 | .058 | 1.793 |
| N of Valid Cases | 26 |  |  |

**DVT responden * kriteria LDL**

| **Crosstab** |  |  |  |  |  |
| --- | --- | --- | --- | --- | --- |
|  |  |  | kriteria LDL |  | Total |
|  |  |  | tinggi >= 130 | normal <130 |  |
| DVT responden | positif | Count | 0 | 4 | 4 |
|  |  | Expected Count | .9 | 3.1 | 4.0 |
|  | negatif | Count | 6 | 16 | 22 |
|  |  | Expected Count | 5.1 | 16.9 | 22.0 |
| Total |  | Count | 6 | 20 | 26 |
|  |  | Expected Count | 6.0 | 20.0 | 26.0 |

| **Chi-Square Tests** |  |  |  |  |  |
| --- | --- | --- | --- | --- | --- |
|  | Value | df | Asymptotic Significance (2-sided) | Exact Sig. (2-sided) | Exact Sig. (1-sided) |
| Pearson Chi-Square | 1.418^a^ | 1 | .234 |  |  |
| Continuity Correction^b^ | .298 | 1 | .585 |  |  |
| Likelihood Ratio | 2.309 | 1 | .129 |  |  |
| Fisher's Exact Test |  |  |  | .542 | .324 |
| Linear-by-Linear Association | 1.364 | 1 | .243 |  |  |
| N of Valid Cases | 26 |  |  |  |  |

| a. 2 cells (50,0%) have expected count less than 5. The minimum expected count is ,92. |  |  |  |  |  |
| --- | --- | --- | --- | --- | --- |
| b. Computed only for a 2x2 table |  |  |  |  |  |

| **Symmetric Measures** |  |  |  |  |  |
| --- | --- | --- | --- | --- | --- |
|  |  | Value | Asymptotic Standard Error^a^ | Approximate T^b^ | Approximate Significance |
| Ordinal by Ordinal | Kendall's tau-b | -.234 | .073 | -1.951 | .051 |
|  | Kendall's tau-c | -.142 | .073 | -1.951 | .051 |
| N of Valid Cases |  | 26 |  |  |  |

| a. Not assuming the null hypothesis. |  |  |  |  |  |
| --- | --- | --- | --- | --- | --- |
| b. Using the asymptotic standard error assuming the null hypothesis. |  |  |  |  |  |

| **Risk Estimate** |  |  |  |
| --- | --- | --- | --- |
|  | Value | 95% Confidence Interval |  |
|  |  | Lower | Upper |
| For cohort kriteria LDL = normal <130 | 1.375 | 1.065 | 1.776 |
| N of Valid Cases | 26 |  |  |

**DVT responden * kriteria trigliserida**

| **Crosstab** |  |  |  |  |  |
| --- | --- | --- | --- | --- | --- |
|  |  |  | kriteria trigliserida |  | Total |
|  |  |  | tinggi >150 | normal <=150 |  |
| DVT responden | positif | Count | 1 | 3 | 4 |
|  |  | Expected Count | .6 | 3.4 | 4.0 |
|  | negatif | Count | 3 | 19 | 22 |
|  |  | Expected Count | 3.4 | 18.6 | 22.0 |
| Total |  | Count | 4 | 22 | 26 |
|  |  | Expected Count | 4.0 | 22.0 | 26.0 |

| **Chi-Square Tests** |  |  |  |  |  |
| --- | --- | --- | --- | --- | --- |
|  | Value | df | Asymptotic Significance (2-sided) | Exact Sig. (2-sided) | Exact Sig. (1-sided) |
| Pearson Chi-Square | .336^a^ | 1 | .562 |  |  |
| Continuity Correction^b^ | .000 | 1 | 1.000 |  |  |
| Likelihood Ratio | .301 | 1 | .584 |  |  |
| Fisher's Exact Test |  |  |  | .511 | .511 |
| Linear-by-Linear Association | .323 | 1 | .570 |  |  |
| N of Valid Cases | 26 |  |  |  |  |

| a. 3 cells (75,0%) have expected count less than 5. The minimum expected count is ,62. |  |  |  |  |  |
| --- | --- | --- | --- | --- | --- |
| b. Computed only for a 2x2 table |  |  |  |  |  |

| **Symmetric Measures** |  |  |  |  |  |
| --- | --- | --- | --- | --- | --- |
|  |  | Value | Asymptotic Standard Error^a^ | Approximate T^b^ | Approximate Significance |
| Ordinal by Ordinal | Kendall's tau-b | .114 | .227 | .489 | .625 |
|  | Kendall's tau-c | .059 | .121 | .489 | .625 |
| N of Valid Cases |  | 26 |  |  |  |

| a. Not assuming the null hypothesis. |  |  |  |  |  |
| --- | --- | --- | --- | --- | --- |
| b. Using the asymptotic standard error assuming the null hypothesis. |  |  |  |  |  |

| **Risk Estimate** |  |  |  |
| --- | --- | --- | --- |
|  | Value | 95% Confidence Interval |  |
|  |  | Lower | Upper |
| Odds Ratio for DVT responden (positif / negatif) | 2.111 | .162 | 27.582 |
| For cohort kriteria trigliserida = tinggi >150 | 1.833 | .249 | 13.503 |
| For cohort kriteria trigliserida = normal <=150 | .868 | .482 | 1.566 |
| N of Valid Cases | 26 |  |  |

**DVT responden * kriteria tot.col**

| **Crosstab** |  |  |  |  |  |
| --- | --- | --- | --- | --- | --- |
|  |  |  | kriteria tot.col |  | Total |
|  |  |  | tinggi >200 | normal <=200 |  |
| DVT responden | positif | Count | 0 | 4 | 4 |
|  |  | Expected Count | .9 | 3.1 | 4.0 |
|  | negatif | Count | 6 | 16 | 22 |
|  |  | Expected Count | 5.1 | 16.9 | 22.0 |
| Total |  | Count | 6 | 20 | 26 |
|  |  | Expected Count | 6.0 | 20.0 | 26.0 |

| **Chi-Square Tests** |  |  |  |  |  |
| --- | --- | --- | --- | --- | --- |
|  | Value | df | Asymptotic Significance (2-sided) | Exact Sig. (2-sided) | Exact Sig. (1-sided) |
| Pearson Chi-Square | 1.418^a^ | 1 | .234 |  |  |
| Continuity Correction^b^ | .298 | 1 | .585 |  |  |
| Likelihood Ratio | 2.309 | 1 | .129 |  |  |
| Fisher's Exact Test |  |  |  | .542 | .324 |
| Linear-by-Linear Association | 1.364 | 1 | .243 |  |  |
| N of Valid Cases | 26 |  |  |  |  |

| a. 2 cells (50,0%) have expected count less than 5. The minimum expected count is ,92. |  |  |  |  |  |
| --- | --- | --- | --- | --- | --- |
| b. Computed only for a 2x2 table |  |  |  |  |  |

| **Symmetric Measures** |  |  |  |  |  |
| --- | --- | --- | --- | --- | --- |
|  |  | Value | Asymptotic Standard Error^a^ | Approximate T^b^ | Approximate Significance |
| Ordinal by Ordinal | Kendall's tau-b | -.234 | .073 | -1.951 | .051 |
|  | Kendall's tau-c | -.142 | .073 | -1.951 | .051 |
| N of Valid Cases |  | 26 |  |  |  |

| a. Not assuming the null hypothesis. |  |  |  |  |  |
| --- | --- | --- | --- | --- | --- |
| b. Using the asymptotic standard error assuming the null hypothesis. |  |  |  |  |  |

| **Risk Estimate** |  |  |  |
| --- | --- | --- | --- |
|  | Value | 95% Confidence Interval |  |
|  |  | Lower | Upper |
| For cohort kriteria tot.col = normal <=200 | 1.375 | 1.065 | 1.776 |
| N of Valid Cases | 26 |  |  |
