# Supplementary material for: Metaphyseal trauma of the lower extremities in major orthopedic surgery as an independent risk factor for deep vein thrombosis
Source: Eur J Orthop Surg Traumatol. 2024 May 23;34(5):2797–803. doi: 10.1007/s00590-024-03960-4 (PMC11291529; doi:10.1007/s00590-024-03960-4)
Supplement: Supplementary file 5 — Supplementary file5 (DOCX 49 kb) [file 590_2024_3960_MOESM5_ESM.docx]

CROSSTABS
 /TABLES=DVTcross BY kriteriausia kriteriaBMI kriteriaFibrinogenH1 hipertensi DM RfibrinogenH1
 stroke talasemia Rlamaoperasi Rtotalperdarahan masalah_jantung Merokok jeniskelamin malgnancy
 kriteriH7fibrinogen RkriteriaDdimerH1 kriteriaH7d_dimer RkriteriaDdimerH7 kriteriHDL kriteriaLDL
 kriteriatrigliserida kriteriatotcolestr
 /FORMAT=AVALUE TABLES
 /STATISTICS=CHISQ BTAU CTAU RISK
 /CELLS=COUNT EXPECTED
 /COUNT ROUND CELL.

**Crosstabs**

| **Notes** |  |  |
| --- | --- | --- |
| Output Created |  | 19-JAN-2024 10:18:28 |
| Comments |  |  |
| Input | Data | C:\Users\nicho\Documents\ortho\101RR_artikel 2 R-2 revisi dr Iwan[1].sav |
|  | Active Dataset | DataSet1 |
|  | Filter | <none> |
|  | Weight | <none> |
|  | Split File | <none> |
|  | N of Rows in Working Data File | 43 |
| Missing Value Handling | Definition of Missing | User-defined missing values are treated as missing. |
|  | Cases Used | Statistics for each table are based on all the cases with valid data in the specified range(s) for all variables in each table. |
| Syntax |  | CROSSTABS /TABLES=DVTcross BY kriteriausia kriteriaBMI kriteriaFibrinogenH1 hipertensi DM RfibrinogenH1 stroke talasemia Rlamaoperasi Rtotalperdarahan masalah_jantung Merokok jeniskelamin malgnancy kriteriH7fibrinogen RkriteriaDdimerH1 kriteriaH7d_dimer RkriteriaDdimerH7 kriteriHDL kriteriaLDL kriteriatrigliserida kriteriatotcolestr /FORMAT=AVALUE TABLES /STATISTICS=CHISQ BTAU CTAU RISK /CELLS=COUNT EXPECTED /COUNT ROUND CELL. |
| Resources | Processor Time | 00:00:00,00 |
|  | Elapsed Time | 00:00:00,04 |
|  | Dimensions Requested | 2 |
|  | Cells Available | 349496 |

| **Case Processing Summary** |  |  |  |  |  |  |
| --- | --- | --- | --- | --- | --- | --- |
|  | Cases |  |  |  |  |  |
|  | Valid |  | Missing |  | Total |  |
|  | N | Percent | N | Percent | N | Percent |
| DVT responden * kriteria usia | 43 | 100.0% | 0 | 0.0% | 43 | 100.0% |
| DVT responden * Kriteria BMi | 43 | 100.0% | 0 | 0.0% | 43 | 100.0% |
| DVT responden * kriteria Fibrinogen H1 | 43 | 100.0% | 0 | 0.0% | 43 | 100.0% |
| DVT responden * Riwayat hipertensi | 43 | 100.0% | 0 | 0.0% | 43 | 100.0% |
| DVT responden * Riwayat DM | 43 | 100.0% | 0 | 0.0% | 43 | 100.0% |
| DVT responden * Rfibrinogen H1 | 43 | 100.0% | 0 | 0.0% | 43 | 100.0% |
| DVT responden * Riwatat stroke | 43 | 100.0% | 0 | 0.0% | 43 | 100.0% |
| DVT responden * Riwayat talasemia pasien | 43 | 100.0% | 0 | 0.0% | 43 | 100.0% |
| DVT responden * R lama operasi | 43 | 100.0% | 0 | 0.0% | 43 | 100.0% |
| DVT responden * R totalperdarahn | 43 | 100.0% | 0 | 0.0% | 43 | 100.0% |
| DVT responden * Riwayat jantung | 43 | 100.0% | 0 | 0.0% | 43 | 100.0% |
| DVT responden * Riwayat merokok | 43 | 100.0% | 0 | 0.0% | 43 | 100.0% |
| DVT responden * jenis kelamin responden | 43 | 100.0% | 0 | 0.0% | 43 | 100.0% |
| DVT responden * Malignancy | 43 | 100.0% | 0 | 0.0% | 43 | 100.0% |
| DVT responden * kriteriaH7 fibrinogen | 43 | 100.0% | 0 | 0.0% | 43 | 100.0% |
| DVT responden * RkriteriaDdimer H1 | 43 | 100.0% | 0 | 0.0% | 43 | 100.0% |
| DVT responden * kriteria H7 D-dimer | 43 | 100.0% | 0 | 0.0% | 43 | 100.0% |
| DVT responden * RKriteria DdimerH7 | 43 | 100.0% | 0 | 0.0% | 43 | 100.0% |
| DVT responden * kriteria HDL | 43 | 100.0% | 0 | 0.0% | 43 | 100.0% |
| DVT responden * kriteria LDL | 43 | 100.0% | 0 | 0.0% | 43 | 100.0% |
| DVT responden * kriteria trigliserida | 43 | 100.0% | 0 | 0.0% | 43 | 100.0% |
| DVT responden * kriteria tot.col | 43 | 100.0% | 0 | 0.0% | 43 | 100.0% |

**DVT responden * kriteria usia**

| **Crosstab** |  |  |  |  |  |
| --- | --- | --- | --- | --- | --- |
|  |  |  | kriteria usia |  | Total |
|  |  |  | >=71 | 50-70 |  |
| DVT responden | positif | Count | 9 | 7 | 16 |
|  |  | Expected Count | 7.1 | 8.9 | 16.0 |
|  | negatif | Count | 10 | 17 | 27 |
|  |  | Expected Count | 11.9 | 15.1 | 27.0 |
| Total |  | Count | 19 | 24 | 43 |
|  |  | Expected Count | 19.0 | 24.0 | 43.0 |

| **Chi-Square Tests** |  |  |  |  |  |
| --- | --- | --- | --- | --- | --- |
|  | Value | df | Asymptotic Significance (2-sided) | Exact Sig. (2-sided) | Exact Sig. (1-sided) |
| Pearson Chi-Square | 1.504^a^ | 1 | .220 |  |  |
| Continuity Correction^b^ | .826 | 1 | .364 |  |  |
| Likelihood Ratio | 1.504 | 1 | .220 |  |  |
| Fisher's Exact Test |  |  |  | .341 | .182 |
| Linear-by-Linear Association | 1.469 | 1 | .226 |  |  |
| N of Valid Cases | 43 |  |  |  |  |

| a. 0 cells (,0%) have expected count less than 5. The minimum expected count is 7,07. |  |  |  |  |  |
| --- | --- | --- | --- | --- | --- |
| b. Computed only for a 2x2 table |  |  |  |  |  |

| **Symmetric Measures** |  |  |  |  |  |
| --- | --- | --- | --- | --- | --- |
|  |  | Value | Asymptotic Standard Error^a^ | Approximate T^b^ | Approximate Significance |
| Ordinal by Ordinal | Kendall's tau-b | .187 | .151 | 1.234 | .217 |
|  | Kendall's tau-c | .180 | .146 | 1.234 | .217 |
| N of Valid Cases |  | 43 |  |  |  |

| a. Not assuming the null hypothesis. |  |  |  |  |  |
| --- | --- | --- | --- | --- | --- |
| b. Using the asymptotic standard error assuming the null hypothesis. |  |  |  |  |  |

| **Risk Estimate** |  |  |  |
| --- | --- | --- | --- |
|  | Value | 95% Confidence Interval |  |
|  |  | Lower | Upper |
| Odds Ratio for DVT responden (positif / negatif) | 2.186 | .620 | 7.700 |
| For cohort kriteria usia = >=71 | 1.519 | .789 | 2.923 |
| For cohort kriteria usia = 50-70 | .695 | .371 | 1.300 |
| N of Valid Cases | 43 |  |  |

**DVT responden * Kriteria BMi**

| **Crosstab** |  |  |  |  |  |
| --- | --- | --- | --- | --- | --- |
|  |  |  | Kriteria BMi |  | Total |
|  |  |  | overweight >=25.0 | normal <25 |  |
| DVT responden | positif | Count | 9 | 7 | 16 |
|  |  | Expected Count | 8.6 | 7.4 | 16.0 |
|  | negatif | Count | 14 | 13 | 27 |
|  |  | Expected Count | 14.4 | 12.6 | 27.0 |
| Total |  | Count | 23 | 20 | 43 |
|  |  | Expected Count | 23.0 | 20.0 | 43.0 |

| **Chi-Square Tests** |  |  |  |  |  |
| --- | --- | --- | --- | --- | --- |
|  | Value | df | Asymptotic Significance (2-sided) | Exact Sig. (2-sided) | Exact Sig. (1-sided) |
| Pearson Chi-Square | .078^a^ | 1 | .780 |  |  |
| Continuity Correction^b^ | .000 | 1 | 1.000 |  |  |
| Likelihood Ratio | .078 | 1 | .780 |  |  |
| Fisher's Exact Test |  |  |  | 1.000 | .515 |
| Linear-by-Linear Association | .076 | 1 | .782 |  |  |
| N of Valid Cases | 43 |  |  |  |  |

| a. 0 cells (,0%) have expected count less than 5. The minimum expected count is 7,44. |  |  |  |  |  |
| --- | --- | --- | --- | --- | --- |
| b. Computed only for a 2x2 table |  |  |  |  |  |

| **Symmetric Measures** |  |  |  |  |  |
| --- | --- | --- | --- | --- | --- |
|  |  | Value | Asymptotic Standard Error^a^ | Approximate T^b^ | Approximate Significance |
| Ordinal by Ordinal | Kendall's tau-b | .043 | .152 | .280 | .779 |
|  | Kendall's tau-c | .041 | .147 | .280 | .779 |
| N of Valid Cases |  | 43 |  |  |  |

| a. Not assuming the null hypothesis. |  |  |  |  |  |
| --- | --- | --- | --- | --- | --- |
| b. Using the asymptotic standard error assuming the null hypothesis. |  |  |  |  |  |

| **Risk Estimate** |  |  |  |
| --- | --- | --- | --- |
|  | Value | 95% Confidence Interval |  |
|  |  | Lower | Upper |
| Odds Ratio for DVT responden (positif / negatif) | 1.194 | .344 | 4.139 |
| For cohort Kriteria BMi = overweight >=25.0 | 1.085 | .617 | 1.908 |
| For cohort Kriteria BMi = normal <25 | .909 | .461 | 1.793 |
| N of Valid Cases | 43 |  |  |

**DVT responden * kriteria Fibrinogen H1**

| **Crosstab** |  |  |  |  |  |
| --- | --- | --- | --- | --- | --- |
|  |  |  | kriteria Fibrinogen H1 |  | Total |
|  |  |  | tinggi >400 | normal <=400 |  |
| DVT responden | positif | Count | 4 | 12 | 16 |
|  |  | Expected Count | 3.7 | 12.3 | 16.0 |
|  | negatif | Count | 6 | 21 | 27 |
|  |  | Expected Count | 6.3 | 20.7 | 27.0 |
| Total |  | Count | 10 | 33 | 43 |
|  |  | Expected Count | 10.0 | 33.0 | 43.0 |

| **Chi-Square Tests** |  |  |  |  |  |
| --- | --- | --- | --- | --- | --- |
|  | Value | df | Asymptotic Significance (2-sided) | Exact Sig. (2-sided) | Exact Sig. (1-sided) |
| Pearson Chi-Square | .043^a^ | 1 | .835 |  |  |
| Continuity Correction^b^ | .000 | 1 | 1.000 |  |  |
| Likelihood Ratio | .043 | 1 | .835 |  |  |
| Fisher's Exact Test |  |  |  | 1.000 | .558 |
| Linear-by-Linear Association | .042 | 1 | .837 |  |  |
| N of Valid Cases | 43 |  |  |  |  |

| a. 1 cells (25,0%) have expected count less than 5. The minimum expected count is 3,72. |  |  |  |  |  |
| --- | --- | --- | --- | --- | --- |
| b. Computed only for a 2x2 table |  |  |  |  |  |

| **Symmetric Measures** |  |  |  |  |  |
| --- | --- | --- | --- | --- | --- |
|  |  | Value | Asymptotic Standard Error^a^ | Approximate T^b^ | Approximate Significance |
| Ordinal by Ordinal | Kendall's tau-b | .032 | .154 | .206 | .837 |
|  | Kendall's tau-c | .026 | .126 | .206 | .837 |
| N of Valid Cases |  | 43 |  |  |  |

| a. Not assuming the null hypothesis. |  |  |  |  |  |
| --- | --- | --- | --- | --- | --- |
| b. Using the asymptotic standard error assuming the null hypothesis. |  |  |  |  |  |

| **Risk Estimate** |  |  |  |
| --- | --- | --- | --- |
|  | Value | 95% Confidence Interval |  |
|  |  | Lower | Upper |
| Odds Ratio for DVT responden (positif / negatif) | 1.167 | .274 | 4.976 |
| For cohort kriteria Fibrinogen H1 = tinggi >400 | 1.125 | .373 | 3.392 |
| For cohort kriteria Fibrinogen H1 = normal <=400 | .964 | .681 | 1.365 |
| N of Valid Cases | 43 |  |  |

**DVT responden * Riwayat hipertensi**

| **Crosstab** |  |  |  |  |  |
| --- | --- | --- | --- | --- | --- |
|  |  |  | Riwayat hipertensi |  | Total |
|  |  |  | ya | tdk |  |
| DVT responden | positif | Count | 10 | 6 | 16 |
|  |  | Expected Count | 6.7 | 9.3 | 16.0 |
|  | negatif | Count | 8 | 19 | 27 |
|  |  | Expected Count | 11.3 | 15.7 | 27.0 |
| Total |  | Count | 18 | 25 | 43 |
|  |  | Expected Count | 18.0 | 25.0 | 43.0 |

| **Chi-Square Tests** |  |  |  |  |  |
| --- | --- | --- | --- | --- | --- |
|  | Value | df | Asymptotic Significance (2-sided) | Exact Sig. (2-sided) | Exact Sig. (1-sided) |
| Pearson Chi-Square | 4.460^a^ | 1 | .035 |  |  |
| Continuity Correction^b^ | 3.212 | 1 | .073 |  |  |
| Likelihood Ratio | 4.481 | 1 | .034 |  |  |
| Fisher's Exact Test |  |  |  | .055 | .037 |
| Linear-by-Linear Association | 4.356 | 1 | .037 |  |  |
| N of Valid Cases | 43 |  |  |  |  |

| a. 0 cells (,0%) have expected count less than 5. The minimum expected count is 6,70. |  |  |  |  |  |
| --- | --- | --- | --- | --- | --- |
| b. Computed only for a 2x2 table |  |  |  |  |  |

| **Symmetric Measures** |  |  |  |  |  |
| --- | --- | --- | --- | --- | --- |
|  |  | Value | Asymptotic Standard Error^a^ | Approximate T^b^ | Approximate Significance |
| Ordinal by Ordinal | Kendall's tau-b | .322 | .147 | 2.164 | .030 |
|  | Kendall's tau-c | .307 | .142 | 2.164 | .030 |
| N of Valid Cases |  | 43 |  |  |  |

| a. Not assuming the null hypothesis. |  |  |  |  |  |
| --- | --- | --- | --- | --- | --- |
| b. Using the asymptotic standard error assuming the null hypothesis. |  |  |  |  |  |

| **Risk Estimate** |  |  |  |
| --- | --- | --- | --- |
|  | Value | 95% Confidence Interval |  |
|  |  | Lower | Upper |
| Odds Ratio for DVT responden (positif / negatif) | 3.958 | 1.072 | 14.618 |
| For cohort Riwayat hipertensi = ya | 2.109 | 1.054 | 4.223 |
| For cohort Riwayat hipertensi = tdk | .533 | .270 | 1.050 |
| N of Valid Cases | 43 |  |  |

**DVT responden * Riwayat DM**

| **Crosstab** |  |  |  |  |  |
| --- | --- | --- | --- | --- | --- |
|  |  |  | Riwayat DM |  | Total |
|  |  |  | ya | tdk |  |
| DVT responden | positif | Count | 1 | 15 | 16 |
|  |  | Expected Count | 1.5 | 14.5 | 16.0 |
|  | negatif | Count | 3 | 24 | 27 |
|  |  | Expected Count | 2.5 | 24.5 | 27.0 |
| Total |  | Count | 4 | 39 | 43 |
|  |  | Expected Count | 4.0 | 39.0 | 43.0 |

| **Chi-Square Tests** |  |  |  |  |  |
| --- | --- | --- | --- | --- | --- |
|  | Value | df | Asymptotic Significance (2-sided) | Exact Sig. (2-sided) | Exact Sig. (1-sided) |
| Pearson Chi-Square | .281^a^ | 1 | .596 |  |  |
| Continuity Correction^b^ | .000 | 1 | 1.000 |  |  |
| Likelihood Ratio | .297 | 1 | .586 |  |  |
| Fisher's Exact Test |  |  |  | 1.000 | .521 |
| Linear-by-Linear Association | .275 | 1 | .600 |  |  |
| N of Valid Cases | 43 |  |  |  |  |

| a. 2 cells (50,0%) have expected count less than 5. The minimum expected count is 1,49. |  |  |  |  |  |
| --- | --- | --- | --- | --- | --- |
| b. Computed only for a 2x2 table |  |  |  |  |  |

| **Symmetric Measures** |  |  |  |  |  |
| --- | --- | --- | --- | --- | --- |
|  |  | Value | Asymptotic Standard Error^a^ | Approximate T^b^ | Approximate Significance |
| Ordinal by Ordinal | Kendall's tau-b | -.081 | .139 | -.568 | .570 |
|  | Kendall's tau-c | -.045 | .080 | -.568 | .570 |
| N of Valid Cases |  | 43 |  |  |  |

| a. Not assuming the null hypothesis. |  |  |  |  |  |
| --- | --- | --- | --- | --- | --- |
| b. Using the asymptotic standard error assuming the null hypothesis. |  |  |  |  |  |

| **Risk Estimate** |  |  |  |
| --- | --- | --- | --- |
|  | Value | 95% Confidence Interval |  |
|  |  | Lower | Upper |
| Odds Ratio for DVT responden (positif / negatif) | .533 | .051 | 5.611 |
| For cohort Riwayat DM = ya | .563 | .064 | 4.961 |
| For cohort Riwayat DM = tdk | 1.055 | .878 | 1.268 |
| N of Valid Cases | 43 |  |  |

**DVT responden * Rfibrinogen H1**

| **Crosstab** |  |  |  |  |  |
| --- | --- | --- | --- | --- | --- |
|  |  |  | Rfibrinogen H1 |  | Total |
|  |  |  | tinggi | normal |  |
| DVT responden | positif | Count | 4 | 12 | 16 |
|  |  | Expected Count | 3.7 | 12.3 | 16.0 |
|  | negatif | Count | 6 | 21 | 27 |
|  |  | Expected Count | 6.3 | 20.7 | 27.0 |
| Total |  | Count | 10 | 33 | 43 |
|  |  | Expected Count | 10.0 | 33.0 | 43.0 |

| **Chi-Square Tests** |  |  |  |  |  |
| --- | --- | --- | --- | --- | --- |
|  | Value | df | Asymptotic Significance (2-sided) | Exact Sig. (2-sided) | Exact Sig. (1-sided) |
| Pearson Chi-Square | .043^a^ | 1 | .835 |  |  |
| Continuity Correction^b^ | .000 | 1 | 1.000 |  |  |
| Likelihood Ratio | .043 | 1 | .835 |  |  |
| Fisher's Exact Test |  |  |  | 1.000 | .558 |
| Linear-by-Linear Association | .042 | 1 | .837 |  |  |
| N of Valid Cases | 43 |  |  |  |  |

| a. 1 cells (25,0%) have expected count less than 5. The minimum expected count is 3,72. |  |  |  |  |  |
| --- | --- | --- | --- | --- | --- |
| b. Computed only for a 2x2 table |  |  |  |  |  |

| **Symmetric Measures** |  |  |  |  |  |
| --- | --- | --- | --- | --- | --- |
|  |  | Value | Asymptotic Standard Error^a^ | Approximate T^b^ | Approximate Significance |
| Ordinal by Ordinal | Kendall's tau-b | .032 | .154 | .206 | .837 |
|  | Kendall's tau-c | .026 | .126 | .206 | .837 |
| N of Valid Cases |  | 43 |  |  |  |

| a. Not assuming the null hypothesis. |  |  |  |  |  |
| --- | --- | --- | --- | --- | --- |
| b. Using the asymptotic standard error assuming the null hypothesis. |  |  |  |  |  |

| **Risk Estimate** |  |  |  |
| --- | --- | --- | --- |
|  | Value | 95% Confidence Interval |  |
|  |  | Lower | Upper |
| Odds Ratio for DVT responden (positif / negatif) | 1.167 | .274 | 4.976 |
| For cohort Rfibrinogen H1 = tinggi | 1.125 | .373 | 3.392 |
| For cohort Rfibrinogen H1 = normal | .964 | .681 | 1.365 |
| N of Valid Cases | 43 |  |  |

**DVT responden * Riwatat stroke**

| **Crosstab** |  |  |  |  |  |
| --- | --- | --- | --- | --- | --- |
|  |  |  | Riwatat stroke |  | Total |
|  |  |  | ya | tdk |  |
| DVT responden | positif | Count | 1 | 15 | 16 |
|  |  | Expected Count | .4 | 15.6 | 16.0 |
|  | negatif | Count | 0 | 27 | 27 |
|  |  | Expected Count | .6 | 26.4 | 27.0 |
| Total |  | Count | 1 | 42 | 43 |
|  |  | Expected Count | 1.0 | 42.0 | 43.0 |

| **Chi-Square Tests** |  |  |  |  |  |
| --- | --- | --- | --- | --- | --- |
|  | Value | df | Asymptotic Significance (2-sided) | Exact Sig. (2-sided) | Exact Sig. (1-sided) |
| Pearson Chi-Square | 1.728^a^ | 1 | .189 |  |  |
| Continuity Correction^b^ | .072 | 1 | .789 |  |  |
| Likelihood Ratio | 2.018 | 1 | .155 |  |  |
| Fisher's Exact Test |  |  |  | .372 | .372 |
| Linear-by-Linear Association | 1.688 | 1 | .194 |  |  |
| N of Valid Cases | 43 |  |  |  |  |

| a. 2 cells (50,0%) have expected count less than 5. The minimum expected count is ,37. |  |  |  |  |  |
| --- | --- | --- | --- | --- | --- |
| b. Computed only for a 2x2 table |  |  |  |  |  |

| **Symmetric Measures** |  |  |  |  |  |
| --- | --- | --- | --- | --- | --- |
|  |  | Value | Asymptotic Standard Error^a^ | Approximate T^b^ | Approximate Significance |
| Ordinal by Ordinal | Kendall's tau-b | .200 | .100 | 1.029 | .303 |
|  | Kendall's tau-c | .058 | .057 | 1.029 | .303 |
| N of Valid Cases |  | 43 |  |  |  |

| a. Not assuming the null hypothesis. |  |  |  |  |  |
| --- | --- | --- | --- | --- | --- |
| b. Using the asymptotic standard error assuming the null hypothesis. |  |  |  |  |  |

| **Risk Estimate** |  |  |  |
| --- | --- | --- | --- |
|  | Value | 95% Confidence Interval |  |
|  |  | Lower | Upper |
| For cohort Riwatat stroke = tdk | .938 | .826 | 1.064 |
| N of Valid Cases | 43 |  |  |

**DVT responden * Riwayat talasemia pasien**

| **Crosstab** |  |  |  |  |
| --- | --- | --- | --- | --- |
|  |  |  | Riwayat talasemia pasien | Total |
|  |  |  | tdk |  |
| DVT responden | positif | Count | 16 | 16 |
|  |  | Expected Count | 16.0 | 16.0 |
|  | negatif | Count | 27 | 27 |
|  |  | Expected Count | 27.0 | 27.0 |
| Total |  | Count | 43 | 43 |
|  |  | Expected Count | 43.0 | 43.0 |

| **Chi-Square Tests** |  |
| --- | --- |
|  | Value |
| Pearson Chi-Square | .^a^ |
| N of Valid Cases | 43 |

| a. No statistics are computed because Riwayat talasemia pasien is a constant. |  |
| --- | --- |

| **Symmetric Measures** |  |  |
| --- | --- | --- |
|  |  | Value |
| Ordinal by Ordinal | Kendall's tau-b | .^a^ |
| N of Valid Cases |  | 43 |

| a. No statistics are computed because Riwayat talasemia pasien is a constant. |  |  |
| --- | --- | --- |

| **Risk Estimate** |  |
| --- | --- |
|  | Value |
| Odds Ratio for DVT responden (positif / negatif) | .^a^ |

| a. No statistics are computed because Riwayat talasemia pasien is a constant. |  |
| --- | --- |

**DVT responden * R lama operasi**

| **Crosstab** |  |  |  |  |  |
| --- | --- | --- | --- | --- | --- |
|  |  |  | R lama operasi |  | Total |
|  |  |  | >= 150 | <150 |  |
| DVT responden | positif | Count | 10 | 6 | 16 |
|  |  | Expected Count | 9.7 | 6.3 | 16.0 |
|  | negatif | Count | 16 | 11 | 27 |
|  |  | Expected Count | 16.3 | 10.7 | 27.0 |
| Total |  | Count | 26 | 17 | 43 |
|  |  | Expected Count | 26.0 | 17.0 | 43.0 |

| **Chi-Square Tests** |  |  |  |  |  |
| --- | --- | --- | --- | --- | --- |
|  | Value | df | Asymptotic Significance (2-sided) | Exact Sig. (2-sided) | Exact Sig. (1-sided) |
| Pearson Chi-Square | .044^a^ | 1 | .834 |  |  |
| Continuity Correction^b^ | .000 | 1 | 1.000 |  |  |
| Likelihood Ratio | .044 | 1 | .833 |  |  |
| Fisher's Exact Test |  |  |  | 1.000 | .547 |
| Linear-by-Linear Association | .043 | 1 | .836 |  |  |
| N of Valid Cases | 43 |  |  |  |  |

| a. 0 cells (,0%) have expected count less than 5. The minimum expected count is 6,33. |  |  |  |  |  |
| --- | --- | --- | --- | --- | --- |
| b. Computed only for a 2x2 table |  |  |  |  |  |

| **Symmetric Measures** |  |  |  |  |  |
| --- | --- | --- | --- | --- | --- |
|  |  | Value | Asymptotic Standard Error^a^ | Approximate T^b^ | Approximate Significance |
| Ordinal by Ordinal | Kendall's tau-b | .032 | .152 | .211 | .833 |
|  | Kendall's tau-c | .030 | .144 | .211 | .833 |
| N of Valid Cases |  | 43 |  |  |  |

| a. Not assuming the null hypothesis. |  |  |  |  |  |
| --- | --- | --- | --- | --- | --- |
| b. Using the asymptotic standard error assuming the null hypothesis. |  |  |  |  |  |

| **Risk Estimate** |  |  |  |
| --- | --- | --- | --- |
|  | Value | 95% Confidence Interval |  |
|  |  | Lower | Upper |
| Odds Ratio for DVT responden (positif / negatif) | 1.146 | .322 | 4.081 |
| For cohort R lama operasi = >= 150 | 1.055 | .645 | 1.725 |
| For cohort R lama operasi = <150 | .920 | .422 | 2.006 |
| N of Valid Cases | 43 |  |  |

**DVT responden * R totalperdarahn**

| **Crosstab** |  |  |  |  |  |
| --- | --- | --- | --- | --- | --- |
|  |  |  | R totalperdarahn |  | Total |
|  |  |  | >=500 | <500 |  |
| DVT responden | positif | Count | 13 | 3 | 16 |
|  |  | Expected Count | 11.9 | 4.1 | 16.0 |
|  | negatif | Count | 19 | 8 | 27 |
|  |  | Expected Count | 20.1 | 6.9 | 27.0 |
| Total |  | Count | 32 | 11 | 43 |
|  |  | Expected Count | 32.0 | 11.0 | 43.0 |

| **Chi-Square Tests** |  |  |  |  |  |
| --- | --- | --- | --- | --- | --- |
|  | Value | df | Asymptotic Significance (2-sided) | Exact Sig. (2-sided) | Exact Sig. (1-sided) |
| Pearson Chi-Square | .625^a^ | 1 | .429 |  |  |
| Continuity Correction^b^ | .184 | 1 | .668 |  |  |
| Likelihood Ratio | .644 | 1 | .422 |  |  |
| Fisher's Exact Test |  |  |  | .494 | .340 |
| Linear-by-Linear Association | .610 | 1 | .435 |  |  |
| N of Valid Cases | 43 |  |  |  |  |

| a. 1 cells (25,0%) have expected count less than 5. The minimum expected count is 4,09. |  |  |  |  |  |
| --- | --- | --- | --- | --- | --- |
| b. Computed only for a 2x2 table |  |  |  |  |  |

| **Symmetric Measures** |  |  |  |  |  |
| --- | --- | --- | --- | --- | --- |
|  |  | Value | Asymptotic Standard Error^a^ | Approximate T^b^ | Approximate Significance |
| Ordinal by Ordinal | Kendall's tau-b | .121 | .144 | .827 | .408 |
|  | Kendall's tau-c | .102 | .123 | .827 | .408 |
| N of Valid Cases |  | 43 |  |  |  |

| a. Not assuming the null hypothesis. |  |  |  |  |  |
| --- | --- | --- | --- | --- | --- |
| b. Using the asymptotic standard error assuming the null hypothesis. |  |  |  |  |  |

| **Risk Estimate** |  |  |  |
| --- | --- | --- | --- |
|  | Value | 95% Confidence Interval |  |
|  |  | Lower | Upper |
| Odds Ratio for DVT responden (positif / negatif) | 1.825 | .406 | 8.200 |
| For cohort R totalperdarahn = >=500 | 1.155 | .822 | 1.621 |
| For cohort R totalperdarahn = <500 | .633 | .196 | 2.047 |
| N of Valid Cases | 43 |  |  |

**DVT responden * Riwayat jantung**

| **Crosstab** |  |  |  |  |  |
| --- | --- | --- | --- | --- | --- |
|  |  |  | Riwayat jantung |  | Total |
|  |  |  | ya | tdk |  |
| DVT responden | positif | Count | 4 | 12 | 16 |
|  |  | Expected Count | 2.6 | 13.4 | 16.0 |
|  | negatif | Count | 3 | 24 | 27 |
|  |  | Expected Count | 4.4 | 22.6 | 27.0 |
| Total |  | Count | 7 | 36 | 43 |
|  |  | Expected Count | 7.0 | 36.0 | 43.0 |

| **Chi-Square Tests** |  |  |  |  |  |
| --- | --- | --- | --- | --- | --- |
|  | Value | df | Asymptotic Significance (2-sided) | Exact Sig. (2-sided) | Exact Sig. (1-sided) |
| Pearson Chi-Square | 1.422^a^ | 1 | .233 |  |  |
| Continuity Correction^b^ | .585 | 1 | .444 |  |  |
| Likelihood Ratio | 1.375 | 1 | .241 |  |  |
| Fisher's Exact Test |  |  |  | .394 | .220 |
| Linear-by-Linear Association | 1.389 | 1 | .239 |  |  |
| N of Valid Cases | 43 |  |  |  |  |

| a. 2 cells (50,0%) have expected count less than 5. The minimum expected count is 2,60. |  |  |  |  |  |
| --- | --- | --- | --- | --- | --- |
| b. Computed only for a 2x2 table |  |  |  |  |  |

| **Symmetric Measures** |  |  |  |  |  |
| --- | --- | --- | --- | --- | --- |
|  |  | Value | Asymptotic Standard Error^a^ | Approximate T^b^ | Approximate Significance |
| Ordinal by Ordinal | Kendall's tau-b | .182 | .157 | 1.115 | .265 |
|  | Kendall's tau-c | .130 | .116 | 1.115 | .265 |
| N of Valid Cases |  | 43 |  |  |  |

| a. Not assuming the null hypothesis. |  |  |  |  |  |
| --- | --- | --- | --- | --- | --- |
| b. Using the asymptotic standard error assuming the null hypothesis. |  |  |  |  |  |

| **Risk Estimate** |  |  |  |
| --- | --- | --- | --- |
|  | Value | 95% Confidence Interval |  |
|  |  | Lower | Upper |
| Odds Ratio for DVT responden (positif / negatif) | 2.667 | .512 | 13.879 |
| For cohort Riwayat jantung = ya | 2.250 | .576 | 8.795 |
| For cohort Riwayat jantung = tdk | .844 | .617 | 1.154 |
| N of Valid Cases | 43 |  |  |

**DVT responden * Riwayat merokok**

| **Crosstab** |  |  |  |  |
| --- | --- | --- | --- | --- |
|  |  |  | Riwayat merokok | Total |
|  |  |  | tdk |  |
| DVT responden | positif | Count | 16 | 16 |
|  |  | Expected Count | 16.0 | 16.0 |
|  | negatif | Count | 27 | 27 |
|  |  | Expected Count | 27.0 | 27.0 |
| Total |  | Count | 43 | 43 |
|  |  | Expected Count | 43.0 | 43.0 |

| **Chi-Square Tests** |  |
| --- | --- |
|  | Value |
| Pearson Chi-Square | .^a^ |
| N of Valid Cases | 43 |

| a. No statistics are computed because Riwayat merokok is a constant. |  |
| --- | --- |

| **Symmetric Measures** |  |  |
| --- | --- | --- |
|  |  | Value |
| Ordinal by Ordinal | Kendall's tau-b | .^a^ |
| N of Valid Cases |  | 43 |

| a. No statistics are computed because Riwayat merokok is a constant. |  |  |
| --- | --- | --- |

| **Risk Estimate** |  |
| --- | --- |
|  | Value |
| Odds Ratio for DVT responden (positif / negatif) | .^a^ |

| a. No statistics are computed because Riwayat merokok is a constant. |  |
| --- | --- |

**DVT responden * jenis kelamin responden**

| **Crosstab** |  |  |  |  |  |
| --- | --- | --- | --- | --- | --- |
|  |  |  | jenis kelamin responden |  | Total |
|  |  |  | P | L |  |
| DVT responden | positif | Count | 13 | 3 | 16 |
|  |  | Expected Count | 13.0 | 3.0 | 16.0 |
|  | negatif | Count | 22 | 5 | 27 |
|  |  | Expected Count | 22.0 | 5.0 | 27.0 |
| Total |  | Count | 35 | 8 | 43 |
|  |  | Expected Count | 35.0 | 8.0 | 43.0 |

| **Chi-Square Tests** |  |  |  |  |  |
| --- | --- | --- | --- | --- | --- |
|  | Value | df | Asymptotic Significance (2-sided) | Exact Sig. (2-sided) | Exact Sig. (1-sided) |
| Pearson Chi-Square | .000^a^ | 1 | .985 |  |  |
| Continuity Correction^b^ | .000 | 1 | 1.000 |  |  |
| Likelihood Ratio | .000 | 1 | .985 |  |  |
| Fisher's Exact Test |  |  |  | 1.000 | .642 |
| Linear-by-Linear Association | .000 | 1 | .985 |  |  |
| N of Valid Cases | 43 |  |  |  |  |

| a. 1 cells (25,0%) have expected count less than 5. The minimum expected count is 2,98. |  |  |  |  |  |
| --- | --- | --- | --- | --- | --- |
| b. Computed only for a 2x2 table |  |  |  |  |  |

| **Symmetric Measures** |  |  |  |  |  |
| --- | --- | --- | --- | --- | --- |
|  |  | Value | Asymptotic Standard Error^a^ | Approximate T^b^ | Approximate Significance |
| Ordinal by Ordinal | Kendall's tau-b | -.003 | .153 | -.019 | .985 |
|  | Kendall's tau-c | -.002 | .115 | -.019 | .985 |
| N of Valid Cases |  | 43 |  |  |  |

| a. Not assuming the null hypothesis. |  |  |  |  |  |
| --- | --- | --- | --- | --- | --- |
| b. Using the asymptotic standard error assuming the null hypothesis. |  |  |  |  |  |

| **Risk Estimate** |  |  |  |
| --- | --- | --- | --- |
|  | Value | 95% Confidence Interval |  |
|  |  | Lower | Upper |
| Odds Ratio for DVT responden (positif / negatif) | .985 | .201 | 4.815 |
| For cohort jenis kelamin responden = P | .997 | .742 | 1.341 |
| For cohort jenis kelamin responden = L | 1.013 | .278 | 3.681 |
| N of Valid Cases | 43 |  |  |

**DVT responden * Malignancy**

| **Crosstab** |  |  |  |  |  |
| --- | --- | --- | --- | --- | --- |
|  |  |  | Malignancy |  | Total |
|  |  |  | ya | tdk |  |
| DVT responden | positif | Count | 1 | 15 | 16 |
|  |  | Expected Count | .4 | 15.6 | 16.0 |
|  | negatif | Count | 0 | 27 | 27 |
|  |  | Expected Count | .6 | 26.4 | 27.0 |
| Total |  | Count | 1 | 42 | 43 |
|  |  | Expected Count | 1.0 | 42.0 | 43.0 |

| **Chi-Square Tests** |  |  |  |  |  |
| --- | --- | --- | --- | --- | --- |
|  | Value | df | Asymptotic Significance (2-sided) | Exact Sig. (2-sided) | Exact Sig. (1-sided) |
| Pearson Chi-Square | 1.728^a^ | 1 | .189 |  |  |
| Continuity Correction^b^ | .072 | 1 | .789 |  |  |
| Likelihood Ratio | 2.018 | 1 | .155 |  |  |
| Fisher's Exact Test |  |  |  | .372 | .372 |
| Linear-by-Linear Association | 1.688 | 1 | .194 |  |  |
| N of Valid Cases | 43 |  |  |  |  |

| a. 2 cells (50,0%) have expected count less than 5. The minimum expected count is ,37. |  |  |  |  |  |
| --- | --- | --- | --- | --- | --- |
| b. Computed only for a 2x2 table |  |  |  |  |  |

| **Symmetric Measures** |  |  |  |  |  |
| --- | --- | --- | --- | --- | --- |
|  |  | Value | Asymptotic Standard Error^a^ | Approximate T^b^ | Approximate Significance |
| Ordinal by Ordinal | Kendall's tau-b | .200 | .100 | 1.029 | .303 |
|  | Kendall's tau-c | .058 | .057 | 1.029 | .303 |
| N of Valid Cases |  | 43 |  |  |  |

| a. Not assuming the null hypothesis. |  |  |  |  |  |
| --- | --- | --- | --- | --- | --- |
| b. Using the asymptotic standard error assuming the null hypothesis. |  |  |  |  |  |

| **Risk Estimate** |  |  |  |
| --- | --- | --- | --- |
|  | Value | 95% Confidence Interval |  |
|  |  | Lower | Upper |
| For cohort Malignancy = tdk | .938 | .826 | 1.064 |
| N of Valid Cases | 43 |  |  |

**DVT responden * kriteriaH7 fibrinogen**

| **Crosstab** |  |  |  |  |  |
| --- | --- | --- | --- | --- | --- |
|  |  |  | kriteriaH7 fibrinogen |  | Total |
|  |  |  | tinggi > 400 | normal < = 400 |  |
| DVT responden | positif | Count | 15 | 1 | 16 |
|  |  | Expected Count | 13.8 | 2.2 | 16.0 |
|  | negatif | Count | 22 | 5 | 27 |
|  |  | Expected Count | 23.2 | 3.8 | 27.0 |
| Total |  | Count | 37 | 6 | 43 |
|  |  | Expected Count | 37.0 | 6.0 | 43.0 |

| **Chi-Square Tests** |  |  |  |  |  |
| --- | --- | --- | --- | --- | --- |
|  | Value | df | Asymptotic Significance (2-sided) | Exact Sig. (2-sided) | Exact Sig. (1-sided) |
| Pearson Chi-Square | 1.259^a^ | 1 | .262 |  |  |
| Continuity Correction^b^ | .445 | 1 | .505 |  |  |
| Likelihood Ratio | 1.398 | 1 | .237 |  |  |
| Fisher's Exact Test |  |  |  | .386 | .260 |
| Linear-by-Linear Association | 1.230 | 1 | .267 |  |  |
| N of Valid Cases | 43 |  |  |  |  |

| a. 2 cells (50,0%) have expected count less than 5. The minimum expected count is 2,23. |  |  |  |  |  |
| --- | --- | --- | --- | --- | --- |
| b. Computed only for a 2x2 table |  |  |  |  |  |

| **Symmetric Measures** |  |  |  |  |  |
| --- | --- | --- | --- | --- | --- |
|  |  | Value | Asymptotic Standard Error^a^ | Approximate T^b^ | Approximate Significance |
| Ordinal by Ordinal | Kendall's tau-b | .171 | .126 | 1.269 | .204 |
|  | Kendall's tau-c | .115 | .090 | 1.269 | .204 |
| N of Valid Cases |  | 43 |  |  |  |

| a. Not assuming the null hypothesis. |  |  |  |  |  |
| --- | --- | --- | --- | --- | --- |
| b. Using the asymptotic standard error assuming the null hypothesis. |  |  |  |  |  |

| **Risk Estimate** |  |  |  |
| --- | --- | --- | --- |
|  | Value | 95% Confidence Interval |  |
|  |  | Lower | Upper |
| Odds Ratio for DVT responden (positif / negatif) | 3.409 | .361 | 32.186 |
| For cohort kriteriaH7 fibrinogen = tinggi > 400 | 1.151 | .923 | 1.434 |
| For cohort kriteriaH7 fibrinogen = normal < = 400 | .338 | .043 | 2.638 |
| N of Valid Cases | 43 |  |  |

**DVT responden * RkriteriaDdimer H1**

| **Crosstab** |  |  |  |  |  |
| --- | --- | --- | --- | --- | --- |
|  |  |  | RkriteriaDdimer H1 |  | Total |
|  |  |  | tinggi | normal |  |
| DVT responden | positif | Count | 2 | 14 | 16 |
|  |  | Expected Count | 3.3 | 12.7 | 16.0 |
|  | negatif | Count | 7 | 20 | 27 |
|  |  | Expected Count | 5.7 | 21.3 | 27.0 |
| Total |  | Count | 9 | 34 | 43 |
|  |  | Expected Count | 9.0 | 34.0 | 43.0 |

| **Chi-Square Tests** |  |  |  |  |  |
| --- | --- | --- | --- | --- | --- |
|  | Value | df | Asymptotic Significance (2-sided) | Exact Sig. (2-sided) | Exact Sig. (1-sided) |
| Pearson Chi-Square | 1.094^a^ | 1 | .296 |  |  |
| Continuity Correction^b^ | .433 | 1 | .510 |  |  |
| Likelihood Ratio | 1.161 | 1 | .281 |  |  |
| Fisher's Exact Test |  |  |  | .446 | .260 |
| Linear-by-Linear Association | 1.069 | 1 | .301 |  |  |
| N of Valid Cases | 43 |  |  |  |  |

| a. 1 cells (25,0%) have expected count less than 5. The minimum expected count is 3,35. |  |  |  |  |  |
| --- | --- | --- | --- | --- | --- |
| b. Computed only for a 2x2 table |  |  |  |  |  |

| **Symmetric Measures** |  |  |  |  |  |
| --- | --- | --- | --- | --- | --- |
|  |  | Value | Asymptotic Standard Error^a^ | Approximate T^b^ | Approximate Significance |
| Ordinal by Ordinal | Kendall's tau-b | -.160 | .137 | -1.132 | .258 |
|  | Kendall's tau-c | -.125 | .111 | -1.132 | .258 |
| N of Valid Cases |  | 43 |  |  |  |

| a. Not assuming the null hypothesis. |  |  |  |  |  |
| --- | --- | --- | --- | --- | --- |
| b. Using the asymptotic standard error assuming the null hypothesis. |  |  |  |  |  |

| **Risk Estimate** |  |  |  |
| --- | --- | --- | --- |
|  | Value | 95% Confidence Interval |  |
|  |  | Lower | Upper |
| Odds Ratio for DVT responden (positif / negatif) | .408 | .074 | 2.265 |
| For cohort RkriteriaDdimer H1 = tinggi | .482 | .114 | 2.045 |
| For cohort RkriteriaDdimer H1 = normal | 1.181 | .884 | 1.579 |
| N of Valid Cases | 43 |  |  |

**DVT responden * kriteria H7 D-dimer**

| **Crosstab** |  |  |  |  |  |
| --- | --- | --- | --- | --- | --- |
|  |  |  | kriteria H7 D-dimer |  | Total |
|  |  |  | tinggi > =500 | normal < 500 |  |
| DVT responden | positif | Count | 14 | 2 | 16 |
|  |  | Expected Count | 13.4 | 2.6 | 16.0 |
|  | negatif | Count | 22 | 5 | 27 |
|  |  | Expected Count | 22.6 | 4.4 | 27.0 |
| Total |  | Count | 36 | 7 | 43 |
|  |  | Expected Count | 36.0 | 7.0 | 43.0 |

| **Chi-Square Tests** |  |  |  |  |  |
| --- | --- | --- | --- | --- | --- |
|  | Value | df | Asymptotic Significance (2-sided) | Exact Sig. (2-sided) | Exact Sig. (1-sided) |
| Pearson Chi-Square | .267^a^ | 1 | .605 |  |  |
| Continuity Correction^b^ | .008 | 1 | .929 |  |  |
| Likelihood Ratio | .276 | 1 | .600 |  |  |
| Fisher's Exact Test |  |  |  | .695 | .475 |
| Linear-by-Linear Association | .261 | 1 | .610 |  |  |
| N of Valid Cases | 43 |  |  |  |  |

| a. 2 cells (50,0%) have expected count less than 5. The minimum expected count is 2,60. |  |  |  |  |  |
| --- | --- | --- | --- | --- | --- |
| b. Computed only for a 2x2 table |  |  |  |  |  |

| **Symmetric Measures** |  |  |  |  |  |
| --- | --- | --- | --- | --- | --- |
|  |  | Value | Asymptotic Standard Error^a^ | Approximate T^b^ | Approximate Significance |
| Ordinal by Ordinal | Kendall's tau-b | .079 | .145 | .539 | .590 |
|  | Kendall's tau-c | .056 | .104 | .539 | .590 |
| N of Valid Cases |  | 43 |  |  |  |

| a. Not assuming the null hypothesis. |  |  |  |  |  |
| --- | --- | --- | --- | --- | --- |
| b. Using the asymptotic standard error assuming the null hypothesis. |  |  |  |  |  |

| **Risk Estimate** |  |  |  |
| --- | --- | --- | --- |
|  | Value | 95% Confidence Interval |  |
|  |  | Lower | Upper |
| Odds Ratio for DVT responden (positif / negatif) | 1.591 | .271 | 9.354 |
| For cohort kriteria H7 D-dimer = tinggi > =500 | 1.074 | .830 | 1.390 |
| For cohort kriteria H7 D-dimer = normal < 500 | .675 | .148 | 3.082 |
| N of Valid Cases | 43 |  |  |

**DVT responden * RKriteria DdimerH7**

| **Crosstab** |  |  |  |  |  |
| --- | --- | --- | --- | --- | --- |
|  |  |  | RKriteria DdimerH7 |  | Total |
|  |  |  | tinggi | normal |  |
| DVT responden | positif | Count | 14 | 2 | 16 |
|  |  | Expected Count | 13.4 | 2.6 | 16.0 |
|  | negatif | Count | 22 | 5 | 27 |
|  |  | Expected Count | 22.6 | 4.4 | 27.0 |
| Total |  | Count | 36 | 7 | 43 |
|  |  | Expected Count | 36.0 | 7.0 | 43.0 |

| **Chi-Square Tests** |  |  |  |  |  |
| --- | --- | --- | --- | --- | --- |
|  | Value | df | Asymptotic Significance (2-sided) | Exact Sig. (2-sided) | Exact Sig. (1-sided) |
| Pearson Chi-Square | .267^a^ | 1 | .605 |  |  |
| Continuity Correction^b^ | .008 | 1 | .929 |  |  |
| Likelihood Ratio | .276 | 1 | .600 |  |  |
| Fisher's Exact Test |  |  |  | .695 | .475 |
| Linear-by-Linear Association | .261 | 1 | .610 |  |  |
| N of Valid Cases | 43 |  |  |  |  |

| a. 2 cells (50,0%) have expected count less than 5. The minimum expected count is 2,60. |  |  |  |  |  |
| --- | --- | --- | --- | --- | --- |
| b. Computed only for a 2x2 table |  |  |  |  |  |

| **Symmetric Measures** |  |  |  |  |  |
| --- | --- | --- | --- | --- | --- |
|  |  | Value | Asymptotic Standard Error^a^ | Approximate T^b^ | Approximate Significance |
| Ordinal by Ordinal | Kendall's tau-b | .079 | .145 | .539 | .590 |
|  | Kendall's tau-c | .056 | .104 | .539 | .590 |
| N of Valid Cases |  | 43 |  |  |  |

| a. Not assuming the null hypothesis. |  |  |  |  |  |
| --- | --- | --- | --- | --- | --- |
| b. Using the asymptotic standard error assuming the null hypothesis. |  |  |  |  |  |

| **Risk Estimate** |  |  |  |
| --- | --- | --- | --- |
|  | Value | 95% Confidence Interval |  |
|  |  | Lower | Upper |
| Odds Ratio for DVT responden (positif / negatif) | 1.591 | .271 | 9.354 |
| For cohort RKriteria DdimerH7 = tinggi | 1.074 | .830 | 1.390 |
| For cohort RKriteria DdimerH7 = normal | .675 | .148 | 3.082 |
| N of Valid Cases | 43 |  |  |

**DVT responden * kriteria HDL**

| **Crosstab** |  |  |  |  |  |
| --- | --- | --- | --- | --- | --- |
|  |  |  | kriteria HDL |  | Total |
|  |  |  | rendah <=40 | tinggi >40 |  |
| DVT responden | positif | Count | 3 | 13 | 16 |
|  |  | Expected Count | 2.6 | 13.4 | 16.0 |
|  | negatif | Count | 4 | 23 | 27 |
|  |  | Expected Count | 4.4 | 22.6 | 27.0 |
| Total |  | Count | 7 | 36 | 43 |
|  |  | Expected Count | 7.0 | 36.0 | 43.0 |

| **Chi-Square Tests** |  |  |  |  |  |
| --- | --- | --- | --- | --- | --- |
|  | Value | df | Asymptotic Significance (2-sided) | Exact Sig. (2-sided) | Exact Sig. (1-sided) |
| Pearson Chi-Square | .114^a^ | 1 | .735 |  |  |
| Continuity Correction^b^ | .000 | 1 | 1.000 |  |  |
| Likelihood Ratio | .113 | 1 | .737 |  |  |
| Fisher's Exact Test |  |  |  | 1.000 | .525 |
| Linear-by-Linear Association | .111 | 1 | .738 |  |  |
| N of Valid Cases | 43 |  |  |  |  |

| a. 2 cells (50,0%) have expected count less than 5. The minimum expected count is 2,60. |  |  |  |  |  |
| --- | --- | --- | --- | --- | --- |
| b. Computed only for a 2x2 table |  |  |  |  |  |

| **Symmetric Measures** |  |  |  |  |  |
| --- | --- | --- | --- | --- | --- |
|  |  | Value | Asymptotic Standard Error^a^ | Approximate T^b^ | Approximate Significance |
| Ordinal by Ordinal | Kendall's tau-b | .052 | .156 | .330 | .741 |
|  | Kendall's tau-c | .037 | .111 | .330 | .741 |
| N of Valid Cases |  | 43 |  |  |  |

| a. Not assuming the null hypothesis. |  |  |  |  |  |
| --- | --- | --- | --- | --- | --- |
| b. Using the asymptotic standard error assuming the null hypothesis. |  |  |  |  |  |

| **Risk Estimate** |  |  |  |
| --- | --- | --- | --- |
|  | Value | 95% Confidence Interval |  |
|  |  | Lower | Upper |
| Odds Ratio for DVT responden (positif / negatif) | 1.327 | .256 | 6.869 |
| For cohort kriteria HDL = rendah <=40 | 1.266 | .324 | 4.947 |
| For cohort kriteria HDL = tinggi >40 | .954 | .719 | 1.266 |
| N of Valid Cases | 43 |  |  |

**DVT responden * kriteria LDL**

| **Crosstab** |  |  |  |  |  |
| --- | --- | --- | --- | --- | --- |
|  |  |  | kriteria LDL |  | Total |
|  |  |  | tinggi >= 130 | normal <130 |  |
| DVT responden | positif | Count | 10 | 6 | 16 |
|  |  | Expected Count | 7.4 | 8.6 | 16.0 |
|  | negatif | Count | 10 | 17 | 27 |
|  |  | Expected Count | 12.6 | 14.4 | 27.0 |
| Total |  | Count | 20 | 23 | 43 |
|  |  | Expected Count | 20.0 | 23.0 | 43.0 |

| **Chi-Square Tests** |  |  |  |  |  |
| --- | --- | --- | --- | --- | --- |
|  | Value | df | Asymptotic Significance (2-sided) | Exact Sig. (2-sided) | Exact Sig. (1-sided) |
| Pearson Chi-Square | 2.618^a^ | 1 | .106 |  |  |
| Continuity Correction^b^ | 1.695 | 1 | .193 |  |  |
| Likelihood Ratio | 2.637 | 1 | .104 |  |  |
| Fisher's Exact Test |  |  |  | .127 | .096 |
| Linear-by-Linear Association | 2.557 | 1 | .110 |  |  |
| N of Valid Cases | 43 |  |  |  |  |

| a. 0 cells (,0%) have expected count less than 5. The minimum expected count is 7,44. |  |  |  |  |  |
| --- | --- | --- | --- | --- | --- |
| b. Computed only for a 2x2 table |  |  |  |  |  |

| **Symmetric Measures** |  |  |  |  |  |
| --- | --- | --- | --- | --- | --- |
|  |  | Value | Asymptotic Standard Error^a^ | Approximate T^b^ | Approximate Significance |
| Ordinal by Ordinal | Kendall's tau-b | .247 | .148 | 1.654 | .098 |
|  | Kendall's tau-c | .238 | .144 | 1.654 | .098 |
| N of Valid Cases |  | 43 |  |  |  |

| a. Not assuming the null hypothesis. |  |  |  |  |  |
| --- | --- | --- | --- | --- | --- |
| b. Using the asymptotic standard error assuming the null hypothesis. |  |  |  |  |  |

| **Risk Estimate** |  |  |  |
| --- | --- | --- | --- |
|  | Value | 95% Confidence Interval |  |
|  |  | Lower | Upper |
| Odds Ratio for DVT responden (positif / negatif) | 2.833 | .789 | 10.175 |
| For cohort kriteria LDL = tinggi >= 130 | 1.688 | .907 | 3.141 |
| For cohort kriteria LDL = normal <130 | .596 | .297 | 1.194 |
| N of Valid Cases | 43 |  |  |

**DVT responden * kriteria trigliserida**

| **Crosstab** |  |  |  |  |  |
| --- | --- | --- | --- | --- | --- |
|  |  |  | kriteria trigliserida |  | Total |
|  |  |  | tinggi >150 | normal <=150 |  |
| DVT responden | positif | Count | 4 | 12 | 16 |
|  |  | Expected Count | 3.7 | 12.3 | 16.0 |
|  | negatif | Count | 6 | 21 | 27 |
|  |  | Expected Count | 6.3 | 20.7 | 27.0 |
| Total |  | Count | 10 | 33 | 43 |
|  |  | Expected Count | 10.0 | 33.0 | 43.0 |

| **Chi-Square Tests** |  |  |  |  |  |
| --- | --- | --- | --- | --- | --- |
|  | Value | df | Asymptotic Significance (2-sided) | Exact Sig. (2-sided) | Exact Sig. (1-sided) |
| Pearson Chi-Square | .043^a^ | 1 | .835 |  |  |
| Continuity Correction^b^ | .000 | 1 | 1.000 |  |  |
| Likelihood Ratio | .043 | 1 | .835 |  |  |
| Fisher's Exact Test |  |  |  | 1.000 | .558 |
| Linear-by-Linear Association | .042 | 1 | .837 |  |  |
| N of Valid Cases | 43 |  |  |  |  |

| a. 1 cells (25,0%) have expected count less than 5. The minimum expected count is 3,72. |  |  |  |  |  |
| --- | --- | --- | --- | --- | --- |
| b. Computed only for a 2x2 table |  |  |  |  |  |

| **Symmetric Measures** |  |  |  |  |  |
| --- | --- | --- | --- | --- | --- |
|  |  | Value | Asymptotic Standard Error^a^ | Approximate T^b^ | Approximate Significance |
| Ordinal by Ordinal | Kendall's tau-b | .032 | .154 | .206 | .837 |
|  | Kendall's tau-c | .026 | .126 | .206 | .837 |
| N of Valid Cases |  | 43 |  |  |  |

| a. Not assuming the null hypothesis. |  |  |  |  |  |
| --- | --- | --- | --- | --- | --- |
| b. Using the asymptotic standard error assuming the null hypothesis. |  |  |  |  |  |

| **Risk Estimate** |  |  |  |
| --- | --- | --- | --- |
|  | Value | 95% Confidence Interval |  |
|  |  | Lower | Upper |
| Odds Ratio for DVT responden (positif / negatif) | 1.167 | .274 | 4.976 |
| For cohort kriteria trigliserida = tinggi >150 | 1.125 | .373 | 3.392 |
| For cohort kriteria trigliserida = normal <=150 | .964 | .681 | 1.365 |
| N of Valid Cases | 43 |  |  |

**DVT responden * kriteria tot.col**

| **Crosstab** |  |  |  |  |  |
| --- | --- | --- | --- | --- | --- |
|  |  |  | kriteria tot.col |  | Total |
|  |  |  | tinggi >200 | normal <=200 |  |
| DVT responden | positif | Count | 9 | 7 | 16 |
|  |  | Expected Count | 8.2 | 7.8 | 16.0 |
|  | negatif | Count | 13 | 14 | 27 |
|  |  | Expected Count | 13.8 | 13.2 | 27.0 |
| Total |  | Count | 22 | 21 | 43 |
|  |  | Expected Count | 22.0 | 21.0 | 43.0 |

| **Chi-Square Tests** |  |  |  |  |  |
| --- | --- | --- | --- | --- | --- |
|  | Value | df | Asymptotic Significance (2-sided) | Exact Sig. (2-sided) | Exact Sig. (1-sided) |
| Pearson Chi-Square | .264^a^ | 1 | .607 |  |  |
| Continuity Correction^b^ | .039 | 1 | .843 |  |  |
| Likelihood Ratio | .264 | 1 | .607 |  |  |
| Fisher's Exact Test |  |  |  | .755 | .422 |
| Linear-by-Linear Association | .258 | 1 | .612 |  |  |
| N of Valid Cases | 43 |  |  |  |  |

| a. 0 cells (,0%) have expected count less than 5. The minimum expected count is 7,81. |  |  |  |  |  |
| --- | --- | --- | --- | --- | --- |
| b. Computed only for a 2x2 table |  |  |  |  |  |

| **Symmetric Measures** |  |  |  |  |  |
| --- | --- | --- | --- | --- | --- |
|  |  | Value | Asymptotic Standard Error^a^ | Approximate T^b^ | Approximate Significance |
| Ordinal by Ordinal | Kendall's tau-b | .078 | .152 | .516 | .606 |
|  | Kendall's tau-c | .076 | .147 | .516 | .606 |
| N of Valid Cases |  | 43 |  |  |  |

| a. Not assuming the null hypothesis. |  |  |  |  |  |
| --- | --- | --- | --- | --- | --- |
| b. Using the asymptotic standard error assuming the null hypothesis. |  |  |  |  |  |

| **Risk Estimate** |  |  |  |
| --- | --- | --- | --- |
|  | Value | 95% Confidence Interval |  |
|  |  | Lower | Upper |
| Odds Ratio for DVT responden (positif / negatif) | 1.385 | .399 | 4.800 |
| For cohort kriteria tot.col = tinggi >200 | 1.168 | .652 | 2.093 |
| For cohort kriteria tot.col = normal <=200 | .844 | .434 | 1.639 |
| N of Valid Cases | 43 |  |  |
