# Supplementary material for: Metaphyseal trauma of the lower extremities in major orthopedic surgery as an independent risk factor for deep vein thrombosis
Source: Eur J Orthop Surg Traumatol. 2024 May 23;34(5):2797–803. doi: 10.1007/s00590-024-03960-4 (PMC11291529; doi:10.1007/s00590-024-03960-4)
Supplement: Supplementary file 6 — Supplementary file6 (DOCX 52 kb) [file 590_2024_3960_MOESM6_ESM.docx]

CROSSTABS
 /TABLES=DVTcross BY kriteriausia kriteriaBMI kriteriaFibrinogenH1 hipertensi DM RfibrinogenH1
 stroke talasemia Rlamaoperasi Rtotalperdarahan masalah_jantung Merokok jeniskelamin malgnancy
 kriteriH7fibrinogen RkriteriaDdimerH1 kriteriaH7d_dimer RkriteriaDdimerH7 kriteriHDL kriteriaLDL
 kriteriatrigliserida kriteriatotcolestr
 /FORMAT=AVALUE TABLES
 /STATISTICS=CHISQ BTAU CTAU RISK
 /CELLS=COUNT EXPECTED
 /COUNT ROUND CELL.

**Crosstabs**

| **Notes** |  |  |
| --- | --- | --- |
| Output Created |  | 19-JAN-2024 10:16:27 |
| Comments |  |  |
| Input | Data | C:\Users\nicho\Documents\ortho\101RR_artikel 2 R-2 revisi dr Iwan[1].sav |
|  | Active Dataset | DataSet1 |
|  | Filter | <none> |
|  | Weight | <none> |
|  | Split File | <none> |
|  | N of Rows in Working Data File | 101 |
| Missing Value Handling | Definition of Missing | User-defined missing values are treated as missing. |
|  | Cases Used | Statistics for each table are based on all the cases with valid data in the specified range(s) for all variables in each table. |
| Syntax |  | CROSSTABS /TABLES=DVTcross BY kriteriausia kriteriaBMI kriteriaFibrinogenH1 hipertensi DM RfibrinogenH1 stroke talasemia Rlamaoperasi Rtotalperdarahan masalah_jantung Merokok jeniskelamin malgnancy kriteriH7fibrinogen RkriteriaDdimerH1 kriteriaH7d_dimer RkriteriaDdimerH7 kriteriHDL kriteriaLDL kriteriatrigliserida kriteriatotcolestr /FORMAT=AVALUE TABLES /STATISTICS=CHISQ BTAU CTAU RISK /CELLS=COUNT EXPECTED /COUNT ROUND CELL. |
| Resources | Processor Time | 00:00:00,02 |
|  | Elapsed Time | 00:00:00,04 |
|  | Dimensions Requested | 2 |
|  | Cells Available | 349496 |

| **Case Processing Summary** |  |  |  |  |  |  |
| --- | --- | --- | --- | --- | --- | --- |
|  | Cases |  |  |  |  |  |
|  | Valid |  | Missing |  | Total |  |
|  | N | Percent | N | Percent | N | Percent |
| DVT responden * kriteria usia | 101 | 100.0% | 0 | 0.0% | 101 | 100.0% |
| DVT responden * Kriteria BMi | 101 | 100.0% | 0 | 0.0% | 101 | 100.0% |
| DVT responden * kriteria Fibrinogen H1 | 101 | 100.0% | 0 | 0.0% | 101 | 100.0% |
| DVT responden * Riwayat hipertensi | 101 | 100.0% | 0 | 0.0% | 101 | 100.0% |
| DVT responden * Riwayat DM | 101 | 100.0% | 0 | 0.0% | 101 | 100.0% |
| DVT responden * Rfibrinogen H1 | 101 | 100.0% | 0 | 0.0% | 101 | 100.0% |
| DVT responden * Riwatat stroke | 101 | 100.0% | 0 | 0.0% | 101 | 100.0% |
| DVT responden * Riwayat talasemia pasien | 101 | 100.0% | 0 | 0.0% | 101 | 100.0% |
| DVT responden * R lama operasi | 101 | 100.0% | 0 | 0.0% | 101 | 100.0% |
| DVT responden * R totalperdarahn | 101 | 100.0% | 0 | 0.0% | 101 | 100.0% |
| DVT responden * Riwayat jantung | 101 | 100.0% | 0 | 0.0% | 101 | 100.0% |
| DVT responden * Riwayat merokok | 101 | 100.0% | 0 | 0.0% | 101 | 100.0% |
| DVT responden * jenis kelamin responden | 101 | 100.0% | 0 | 0.0% | 101 | 100.0% |
| DVT responden * Malignancy | 101 | 100.0% | 0 | 0.0% | 101 | 100.0% |
| DVT responden * kriteriaH7 fibrinogen | 101 | 100.0% | 0 | 0.0% | 101 | 100.0% |
| DVT responden * RkriteriaDdimer H1 | 101 | 100.0% | 0 | 0.0% | 101 | 100.0% |
| DVT responden * kriteria H7 D-dimer | 101 | 100.0% | 0 | 0.0% | 101 | 100.0% |
| DVT responden * RKriteria DdimerH7 | 101 | 100.0% | 0 | 0.0% | 101 | 100.0% |
| DVT responden * kriteria HDL | 101 | 100.0% | 0 | 0.0% | 101 | 100.0% |
| DVT responden * kriteria LDL | 101 | 100.0% | 0 | 0.0% | 101 | 100.0% |
| DVT responden * kriteria trigliserida | 101 | 100.0% | 0 | 0.0% | 101 | 100.0% |
| DVT responden * kriteria tot.col | 101 | 100.0% | 0 | 0.0% | 101 | 100.0% |

**DVT responden * kriteria usia**

| **Crosstab** |  |  |  |  |  |
| --- | --- | --- | --- | --- | --- |
|  |  |  | kriteria usia |  | Total |
|  |  |  | >=71 | 50-70 |  |
| DVT responden | positif | Count | 16 | 9 | 25 |
|  |  | Expected Count | 15.3 | 9.7 | 25.0 |
|  | negatif | Count | 46 | 30 | 76 |
|  |  | Expected Count | 46.7 | 29.3 | 76.0 |
| Total |  | Count | 62 | 39 | 101 |
|  |  | Expected Count | 62.0 | 39.0 | 101.0 |

| **Chi-Square Tests** |  |  |  |  |  |
| --- | --- | --- | --- | --- | --- |
|  | Value | df | Asymptotic Significance (2-sided) | Exact Sig. (2-sided) | Exact Sig. (1-sided) |
| Pearson Chi-Square | .096^a^ | 1 | .757 |  |  |
| Continuity Correction^b^ | .005 | 1 | .942 |  |  |
| Likelihood Ratio | .096 | 1 | .756 |  |  |
| Fisher's Exact Test |  |  |  | .816 | .475 |
| Linear-by-Linear Association | .095 | 1 | .758 |  |  |
| N of Valid Cases | 101 |  |  |  |  |

| a. 0 cells (,0%) have expected count less than 5. The minimum expected count is 9,65. |  |  |  |  |  |
| --- | --- | --- | --- | --- | --- |
| b. Computed only for a 2x2 table |  |  |  |  |  |

| **Symmetric Measures** |  |  |  |  |  |
| --- | --- | --- | --- | --- | --- |
|  |  | Value | Asymptotic Standard Error^a^ | Approximate T^b^ | Approximate Significance |
| Ordinal by Ordinal | Kendall's tau-b | .031 | .099 | .312 | .755 |
|  | Kendall's tau-c | .026 | .083 | .312 | .755 |
| N of Valid Cases |  | 101 |  |  |  |

| a. Not assuming the null hypothesis. |  |  |  |  |  |
| --- | --- | --- | --- | --- | --- |
| b. Using the asymptotic standard error assuming the null hypothesis. |  |  |  |  |  |

| **Risk Estimate** |  |  |  |
| --- | --- | --- | --- |
|  | Value | 95% Confidence Interval |  |
|  |  | Lower | Upper |
| Odds Ratio for DVT responden (positif / negatif) | 1.159 | .454 | 2.960 |
| For cohort kriteria usia = >=71 | 1.057 | .748 | 1.494 |
| For cohort kriteria usia = 50-70 | .912 | .504 | 1.649 |
| N of Valid Cases | 101 |  |  |

**DVT responden * Kriteria BMi**

| **Crosstab** |  |  |  |  |  |
| --- | --- | --- | --- | --- | --- |
|  |  |  | Kriteria BMi |  | Total |
|  |  |  | overweight >=25.0 | normal <25 |  |
| DVT responden | positif | Count | 10 | 15 | 25 |
|  |  | Expected Count | 7.9 | 17.1 | 25.0 |
|  | negatif | Count | 22 | 54 | 76 |
|  |  | Expected Count | 24.1 | 51.9 | 76.0 |
| Total |  | Count | 32 | 69 | 101 |
|  |  | Expected Count | 32.0 | 69.0 | 101.0 |

| **Chi-Square Tests** |  |  |  |  |  |
| --- | --- | --- | --- | --- | --- |
|  | Value | df | Asymptotic Significance (2-sided) | Exact Sig. (2-sided) | Exact Sig. (1-sided) |
| Pearson Chi-Square | 1.062^a^ | 1 | .303 |  |  |
| Continuity Correction^b^ | .612 | 1 | .434 |  |  |
| Likelihood Ratio | 1.035 | 1 | .309 |  |  |
| Fisher's Exact Test |  |  |  | .329 | .215 |
| Linear-by-Linear Association | 1.051 | 1 | .305 |  |  |
| N of Valid Cases | 101 |  |  |  |  |

| a. 0 cells (,0%) have expected count less than 5. The minimum expected count is 7,92. |  |  |  |  |  |
| --- | --- | --- | --- | --- | --- |
| b. Computed only for a 2x2 table |  |  |  |  |  |

| **Symmetric Measures** |  |  |  |  |  |
| --- | --- | --- | --- | --- | --- |
|  |  | Value | Asymptotic Standard Error^a^ | Approximate T^b^ | Approximate Significance |
| Ordinal by Ordinal | Kendall's tau-b | .103 | .103 | .990 | .322 |
|  | Kendall's tau-c | .082 | .083 | .990 | .322 |
| N of Valid Cases |  | 101 |  |  |  |

| a. Not assuming the null hypothesis. |  |  |  |  |  |
| --- | --- | --- | --- | --- | --- |
| b. Using the asymptotic standard error assuming the null hypothesis. |  |  |  |  |  |

| **Risk Estimate** |  |  |  |
| --- | --- | --- | --- |
|  | Value | 95% Confidence Interval |  |
|  |  | Lower | Upper |
| Odds Ratio for DVT responden (positif / negatif) | 1.636 | .638 | 4.194 |
| For cohort Kriteria BMi = overweight >=25.0 | 1.382 | .762 | 2.506 |
| For cohort Kriteria BMi = normal <25 | .844 | .595 | 1.199 |
| N of Valid Cases | 101 |  |  |

**DVT responden * kriteria Fibrinogen H1**

| **Crosstab** |  |  |  |  |  |
| --- | --- | --- | --- | --- | --- |
|  |  |  | kriteria Fibrinogen H1 |  | Total |
|  |  |  | tinggi >400 | normal <=400 |  |
| DVT responden | positif | Count | 6 | 19 | 25 |
|  |  | Expected Count | 7.4 | 17.6 | 25.0 |
|  | negatif | Count | 24 | 52 | 76 |
|  |  | Expected Count | 22.6 | 53.4 | 76.0 |
| Total |  | Count | 30 | 71 | 101 |
|  |  | Expected Count | 30.0 | 71.0 | 101.0 |

| **Chi-Square Tests** |  |  |  |  |  |
| --- | --- | --- | --- | --- | --- |
|  | Value | df | Asymptotic Significance (2-sided) | Exact Sig. (2-sided) | Exact Sig. (1-sided) |
| Pearson Chi-Square | .518^a^ | 1 | .472 |  |  |
| Continuity Correction^b^ | .218 | 1 | .640 |  |  |
| Likelihood Ratio | .532 | 1 | .466 |  |  |
| Fisher's Exact Test |  |  |  | .616 | .326 |
| Linear-by-Linear Association | .512 | 1 | .474 |  |  |
| N of Valid Cases | 101 |  |  |  |  |

| a. 0 cells (,0%) have expected count less than 5. The minimum expected count is 7,43. |  |  |  |  |  |
| --- | --- | --- | --- | --- | --- |
| b. Computed only for a 2x2 table |  |  |  |  |  |

| **Symmetric Measures** |  |  |  |  |  |
| --- | --- | --- | --- | --- | --- |
|  |  | Value | Asymptotic Standard Error^a^ | Approximate T^b^ | Approximate Significance |
| Ordinal by Ordinal | Kendall's tau-b | -.072 | .095 | -.750 | .453 |
|  | Kendall's tau-c | -.056 | .075 | -.750 | .453 |
| N of Valid Cases |  | 101 |  |  |  |

| a. Not assuming the null hypothesis. |  |  |  |  |  |
| --- | --- | --- | --- | --- | --- |
| b. Using the asymptotic standard error assuming the null hypothesis. |  |  |  |  |  |

| **Risk Estimate** |  |  |  |
| --- | --- | --- | --- |
|  | Value | 95% Confidence Interval |  |
|  |  | Lower | Upper |
| Odds Ratio for DVT responden (positif / negatif) | .684 | .242 | 1.931 |
| For cohort kriteria Fibrinogen H1 = tinggi >400 | .760 | .351 | 1.645 |
| For cohort kriteria Fibrinogen H1 = normal <=400 | 1.111 | .850 | 1.452 |
| N of Valid Cases | 101 |  |  |

**DVT responden * Riwayat hipertensi**

| **Crosstab** |  |  |  |  |  |
| --- | --- | --- | --- | --- | --- |
|  |  |  | Riwayat hipertensi |  | Total |
|  |  |  | ya | tdk |  |
| DVT responden | positif | Count | 17 | 8 | 25 |
|  |  | Expected Count | 12.6 | 12.4 | 25.0 |
|  | negatif | Count | 34 | 42 | 76 |
|  |  | Expected Count | 38.4 | 37.6 | 76.0 |
| Total |  | Count | 51 | 50 | 101 |
|  |  | Expected Count | 51.0 | 50.0 | 101.0 |

| **Chi-Square Tests** |  |  |  |  |  |
| --- | --- | --- | --- | --- | --- |
|  | Value | df | Asymptotic Significance (2-sided) | Exact Sig. (2-sided) | Exact Sig. (1-sided) |
| Pearson Chi-Square | 4.073^a^ | 1 | .044 |  |  |
| Continuity Correction^b^ | 3.195 | 1 | .074 |  |  |
| Likelihood Ratio | 4.148 | 1 | .042 |  |  |
| Fisher's Exact Test |  |  |  | .064 | .036 |
| Linear-by-Linear Association | 4.032 | 1 | .045 |  |  |
| N of Valid Cases | 101 |  |  |  |  |

| a. 0 cells (,0%) have expected count less than 5. The minimum expected count is 12,38. |  |  |  |  |  |
| --- | --- | --- | --- | --- | --- |
| b. Computed only for a 2x2 table |  |  |  |  |  |

| **Symmetric Measures** |  |  |  |  |  |
| --- | --- | --- | --- | --- | --- |
|  |  | Value | Asymptotic Standard Error^a^ | Approximate T^b^ | Approximate Significance |
| Ordinal by Ordinal | Kendall's tau-b | .201 | .095 | 2.065 | .039 |
|  | Kendall's tau-c | .173 | .084 | 2.065 | .039 |
| N of Valid Cases |  | 101 |  |  |  |

| a. Not assuming the null hypothesis. |  |  |  |  |  |
| --- | --- | --- | --- | --- | --- |
| b. Using the asymptotic standard error assuming the null hypothesis. |  |  |  |  |  |

| **Risk Estimate** |  |  |  |
| --- | --- | --- | --- |
|  | Value | 95% Confidence Interval |  |
|  |  | Lower | Upper |
| Odds Ratio for DVT responden (positif / negatif) | 2.625 | 1.011 | 6.816 |
| For cohort Riwayat hipertensi = ya | 1.520 | 1.053 | 2.194 |
| For cohort Riwayat hipertensi = tdk | .579 | .316 | 1.062 |
| N of Valid Cases | 101 |  |  |

**DVT responden * Riwayat DM**

| **Crosstab** |  |  |  |  |  |
| --- | --- | --- | --- | --- | --- |
|  |  |  | Riwayat DM |  | Total |
|  |  |  | ya | tdk |  |
| DVT responden | positif | Count | 4 | 21 | 25 |
|  |  | Expected Count | 4.5 | 20.5 | 25.0 |
|  | negatif | Count | 14 | 62 | 76 |
|  |  | Expected Count | 13.5 | 62.5 | 76.0 |
| Total |  | Count | 18 | 83 | 101 |
|  |  | Expected Count | 18.0 | 83.0 | 101.0 |

| **Chi-Square Tests** |  |  |  |  |  |
| --- | --- | --- | --- | --- | --- |
|  | Value | df | Asymptotic Significance (2-sided) | Exact Sig. (2-sided) | Exact Sig. (1-sided) |
| Pearson Chi-Square | .075^a^ | 1 | .784 |  |  |
| Continuity Correction^b^ | .000 | 1 | 1.000 |  |  |
| Likelihood Ratio | .077 | 1 | .782 |  |  |
| Fisher's Exact Test |  |  |  | 1.000 | .524 |
| Linear-by-Linear Association | .075 | 1 | .785 |  |  |
| N of Valid Cases | 101 |  |  |  |  |

| a. 1 cells (25,0%) have expected count less than 5. The minimum expected count is 4,46. |  |  |  |  |  |
| --- | --- | --- | --- | --- | --- |
| b. Computed only for a 2x2 table |  |  |  |  |  |

| **Symmetric Measures** |  |  |  |  |  |
| --- | --- | --- | --- | --- | --- |
|  |  | Value | Asymptotic Standard Error^a^ | Approximate T^b^ | Approximate Significance |
| Ordinal by Ordinal | Kendall's tau-b | -.027 | .097 | -.282 | .778 |
|  | Kendall's tau-c | -.018 | .064 | -.282 | .778 |
| N of Valid Cases |  | 101 |  |  |  |

| a. Not assuming the null hypothesis. |  |  |  |  |  |
| --- | --- | --- | --- | --- | --- |
| b. Using the asymptotic standard error assuming the null hypothesis. |  |  |  |  |  |

| **Risk Estimate** |  |  |  |
| --- | --- | --- | --- |
|  | Value | 95% Confidence Interval |  |
|  |  | Lower | Upper |
| Odds Ratio for DVT responden (positif / negatif) | .844 | .250 | 2.847 |
| For cohort Riwayat DM = ya | .869 | .315 | 2.397 |
| For cohort Riwayat DM = tdk | 1.030 | .842 | 1.260 |
| N of Valid Cases | 101 |  |  |

**DVT responden * Rfibrinogen H1**

| **Crosstab** |  |  |  |  |  |
| --- | --- | --- | --- | --- | --- |
|  |  |  | Rfibrinogen H1 |  | Total |
|  |  |  | tinggi | normal |  |
| DVT responden | positif | Count | 6 | 19 | 25 |
|  |  | Expected Count | 7.4 | 17.6 | 25.0 |
|  | negatif | Count | 24 | 52 | 76 |
|  |  | Expected Count | 22.6 | 53.4 | 76.0 |
| Total |  | Count | 30 | 71 | 101 |
|  |  | Expected Count | 30.0 | 71.0 | 101.0 |

| **Chi-Square Tests** |  |  |  |  |  |
| --- | --- | --- | --- | --- | --- |
|  | Value | df | Asymptotic Significance (2-sided) | Exact Sig. (2-sided) | Exact Sig. (1-sided) |
| Pearson Chi-Square | .518^a^ | 1 | .472 |  |  |
| Continuity Correction^b^ | .218 | 1 | .640 |  |  |
| Likelihood Ratio | .532 | 1 | .466 |  |  |
| Fisher's Exact Test |  |  |  | .616 | .326 |
| Linear-by-Linear Association | .512 | 1 | .474 |  |  |
| N of Valid Cases | 101 |  |  |  |  |

| a. 0 cells (,0%) have expected count less than 5. The minimum expected count is 7,43. |  |  |  |  |  |
| --- | --- | --- | --- | --- | --- |
| b. Computed only for a 2x2 table |  |  |  |  |  |

| **Symmetric Measures** |  |  |  |  |  |
| --- | --- | --- | --- | --- | --- |
|  |  | Value | Asymptotic Standard Error^a^ | Approximate T^b^ | Approximate Significance |
| Ordinal by Ordinal | Kendall's tau-b | -.072 | .095 | -.750 | .453 |
|  | Kendall's tau-c | -.056 | .075 | -.750 | .453 |
| N of Valid Cases |  | 101 |  |  |  |

| a. Not assuming the null hypothesis. |  |  |  |  |  |
| --- | --- | --- | --- | --- | --- |
| b. Using the asymptotic standard error assuming the null hypothesis. |  |  |  |  |  |

| **Risk Estimate** |  |  |  |
| --- | --- | --- | --- |
|  | Value | 95% Confidence Interval |  |
|  |  | Lower | Upper |
| Odds Ratio for DVT responden (positif / negatif) | .684 | .242 | 1.931 |
| For cohort Rfibrinogen H1 = tinggi | .760 | .351 | 1.645 |
| For cohort Rfibrinogen H1 = normal | 1.111 | .850 | 1.452 |
| N of Valid Cases | 101 |  |  |

**DVT responden * Riwatat stroke**

| **Crosstab** |  |  |  |  |  |
| --- | --- | --- | --- | --- | --- |
|  |  |  | Riwatat stroke |  | Total |
|  |  |  | ya | tdk |  |
| DVT responden | positif | Count | 2 | 23 | 25 |
|  |  | Expected Count | 2.5 | 22.5 | 25.0 |
|  | negatif | Count | 8 | 68 | 76 |
|  |  | Expected Count | 7.5 | 68.5 | 76.0 |
| Total |  | Count | 10 | 91 | 101 |
|  |  | Expected Count | 10.0 | 91.0 | 101.0 |

| **Chi-Square Tests** |  |  |  |  |  |
| --- | --- | --- | --- | --- | --- |
|  | Value | df | Asymptotic Significance (2-sided) | Exact Sig. (2-sided) | Exact Sig. (1-sided) |
| Pearson Chi-Square | .135^a^ | 1 | .714 |  |  |
| Continuity Correction^b^ | .000 | 1 | 1.000 |  |  |
| Likelihood Ratio | .140 | 1 | .708 |  |  |
| Fisher's Exact Test |  |  |  | 1.000 | .529 |
| Linear-by-Linear Association | .133 | 1 | .715 |  |  |
| N of Valid Cases | 101 |  |  |  |  |

| a. 1 cells (25,0%) have expected count less than 5. The minimum expected count is 2,48. |  |  |  |  |  |
| --- | --- | --- | --- | --- | --- |
| b. Computed only for a 2x2 table |  |  |  |  |  |

| **Symmetric Measures** |  |  |  |  |  |
| --- | --- | --- | --- | --- | --- |
|  |  | Value | Asymptotic Standard Error^a^ | Approximate T^b^ | Approximate Significance |
| Ordinal by Ordinal | Kendall's tau-b | -.037 | .093 | -.390 | .696 |
|  | Kendall's tau-c | -.019 | .048 | -.390 | .696 |
| N of Valid Cases |  | 101 |  |  |  |

| a. Not assuming the null hypothesis. |  |  |  |  |  |
| --- | --- | --- | --- | --- | --- |
| b. Using the asymptotic standard error assuming the null hypothesis. |  |  |  |  |  |

| **Risk Estimate** |  |  |  |
| --- | --- | --- | --- |
|  | Value | 95% Confidence Interval |  |
|  |  | Lower | Upper |
| Odds Ratio for DVT responden (positif / negatif) | .739 | .146 | 3.735 |
| For cohort Riwatat stroke = ya | .760 | .173 | 3.346 |
| For cohort Riwatat stroke = tdk | 1.028 | .895 | 1.182 |
| N of Valid Cases | 101 |  |  |

**DVT responden * Riwayat talasemia pasien**

| **Crosstab** |  |  |  |  |  |
| --- | --- | --- | --- | --- | --- |
|  |  |  | Riwayat talasemia pasien |  | Total |
|  |  |  | ya | tdk |  |
| DVT responden | positif | Count | 2 | 23 | 25 |
|  |  | Expected Count | .5 | 24.5 | 25.0 |
|  | negatif | Count | 0 | 76 | 76 |
|  |  | Expected Count | 1.5 | 74.5 | 76.0 |
| Total |  | Count | 2 | 99 | 101 |
|  |  | Expected Count | 2.0 | 99.0 | 101.0 |

| **Chi-Square Tests** |  |  |  |  |  |
| --- | --- | --- | --- | --- | --- |
|  | Value | df | Asymptotic Significance (2-sided) | Exact Sig. (2-sided) | Exact Sig. (1-sided) |
| Pearson Chi-Square | 6.203^a^ | 1 | .013 |  |  |
| Continuity Correction^b^ | 2.766 | 1 | .096 |  |  |
| Likelihood Ratio | 5.710 | 1 | .017 |  |  |
| Fisher's Exact Test |  |  |  | .059 | .059 |
| Linear-by-Linear Association | 6.141 | 1 | .013 |  |  |
| N of Valid Cases | 101 |  |  |  |  |

| a. 2 cells (50,0%) have expected count less than 5. The minimum expected count is ,50. |  |  |  |  |  |
| --- | --- | --- | --- | --- | --- |
| b. Computed only for a 2x2 table |  |  |  |  |  |

| **Symmetric Measures** |  |  |  |  |  |
| --- | --- | --- | --- | --- | --- |
|  |  | Value | Asymptotic Standard Error^a^ | Approximate T^b^ | Approximate Significance |
| Ordinal by Ordinal | Kendall's tau-b | .248 | .086 | 1.453 | .146 |
|  | Kendall's tau-c | .060 | .041 | 1.453 | .146 |
| N of Valid Cases |  | 101 |  |  |  |

| a. Not assuming the null hypothesis. |  |  |  |  |  |
| --- | --- | --- | --- | --- | --- |
| b. Using the asymptotic standard error assuming the null hypothesis. |  |  |  |  |  |

| **Risk Estimate** |  |  |  |
| --- | --- | --- | --- |
|  | Value | 95% Confidence Interval |  |
|  |  | Lower | Upper |
| For cohort Riwayat talasemia pasien = tdk | .920 | .820 | 1.033 |
| N of Valid Cases | 101 |  |  |

**DVT responden * R lama operasi**

| **Crosstab** |  |  |  |  |  |
| --- | --- | --- | --- | --- | --- |
|  |  |  | R lama operasi |  | Total |
|  |  |  | >= 150 | <150 |  |
| DVT responden | positif | Count | 10 | 15 | 25 |
|  |  | Expected Count | 7.2 | 17.8 | 25.0 |
|  | negatif | Count | 19 | 57 | 76 |
|  |  | Expected Count | 21.8 | 54.2 | 76.0 |
| Total |  | Count | 29 | 72 | 101 |
|  |  | Expected Count | 29.0 | 72.0 | 101.0 |

| **Chi-Square Tests** |  |  |  |  |  |
| --- | --- | --- | --- | --- | --- |
|  | Value | df | Asymptotic Significance (2-sided) | Exact Sig. (2-sided) | Exact Sig. (1-sided) |
| Pearson Chi-Square | 2.068^a^ | 1 | .150 |  |  |
| Continuity Correction^b^ | 1.400 | 1 | .237 |  |  |
| Likelihood Ratio | 1.986 | 1 | .159 |  |  |
| Fisher's Exact Test |  |  |  | .202 | .119 |
| Linear-by-Linear Association | 2.047 | 1 | .152 |  |  |
| N of Valid Cases | 101 |  |  |  |  |

| a. 0 cells (,0%) have expected count less than 5. The minimum expected count is 7,18. |  |  |  |  |  |
| --- | --- | --- | --- | --- | --- |
| b. Computed only for a 2x2 table |  |  |  |  |  |

| **Symmetric Measures** |  |  |  |  |  |
| --- | --- | --- | --- | --- | --- |
|  |  | Value | Asymptotic Standard Error^a^ | Approximate T^b^ | Approximate Significance |
| Ordinal by Ordinal | Kendall's tau-b | .143 | .105 | 1.349 | .177 |
|  | Kendall's tau-c | .112 | .083 | 1.349 | .177 |
| N of Valid Cases |  | 101 |  |  |  |

| a. Not assuming the null hypothesis. |  |  |  |  |  |
| --- | --- | --- | --- | --- | --- |
| b. Using the asymptotic standard error assuming the null hypothesis. |  |  |  |  |  |

| **Risk Estimate** |  |  |  |
| --- | --- | --- | --- |
|  | Value | 95% Confidence Interval |  |
|  |  | Lower | Upper |
| Odds Ratio for DVT responden (positif / negatif) | 2.000 | .771 | 5.191 |
| For cohort R lama operasi = >= 150 | 1.600 | .862 | 2.969 |
| For cohort R lama operasi = <150 | .800 | .566 | 1.130 |
| N of Valid Cases | 101 |  |  |

**DVT responden * R totalperdarahn**

| **Crosstab** |  |  |  |  |  |
| --- | --- | --- | --- | --- | --- |
|  |  |  | R totalperdarahn |  | Total |
|  |  |  | >=500 | <500 |  |
| DVT responden | positif | Count | 17 | 8 | 25 |
|  |  | Expected Count | 13.6 | 11.4 | 25.0 |
|  | negatif | Count | 38 | 38 | 76 |
|  |  | Expected Count | 41.4 | 34.6 | 76.0 |
| Total |  | Count | 55 | 46 | 101 |
|  |  | Expected Count | 55.0 | 46.0 | 101.0 |

| **Chi-Square Tests** |  |  |  |  |  |
| --- | --- | --- | --- | --- | --- |
|  | Value | df | Asymptotic Significance (2-sided) | Exact Sig. (2-sided) | Exact Sig. (1-sided) |
| Pearson Chi-Square | 2.458^a^ | 1 | .117 |  |  |
| Continuity Correction^b^ | 1.785 | 1 | .181 |  |  |
| Likelihood Ratio | 2.511 | 1 | .113 |  |  |
| Fisher's Exact Test |  |  |  | .165 | .090 |
| Linear-by-Linear Association | 2.433 | 1 | .119 |  |  |
| N of Valid Cases | 101 |  |  |  |  |

| a. 0 cells (,0%) have expected count less than 5. The minimum expected count is 11,39. |  |  |  |  |  |
| --- | --- | --- | --- | --- | --- |
| b. Computed only for a 2x2 table |  |  |  |  |  |

| **Symmetric Measures** |  |  |  |  |  |
| --- | --- | --- | --- | --- | --- |
|  |  | Value | Asymptotic Standard Error^a^ | Approximate T^b^ | Approximate Significance |
| Ordinal by Ordinal | Kendall's tau-b | .156 | .095 | 1.614 | .106 |
|  | Kendall's tau-c | .134 | .083 | 1.614 | .106 |
| N of Valid Cases |  | 101 |  |  |  |

| a. Not assuming the null hypothesis. |  |  |  |  |  |
| --- | --- | --- | --- | --- | --- |
| b. Using the asymptotic standard error assuming the null hypothesis. |  |  |  |  |  |

| **Risk Estimate** |  |  |  |
| --- | --- | --- | --- |
|  | Value | 95% Confidence Interval |  |
|  |  | Lower | Upper |
| Odds Ratio for DVT responden (positif / negatif) | 2.125 | .819 | 5.511 |
| For cohort R totalperdarahn = >=500 | 1.360 | .958 | 1.931 |
| For cohort R totalperdarahn = <500 | .640 | .346 | 1.183 |
| N of Valid Cases | 101 |  |  |

**DVT responden * Riwayat jantung**

| **Crosstab** |  |  |  |  |  |
| --- | --- | --- | --- | --- | --- |
|  |  |  | Riwayat jantung |  | Total |
|  |  |  | ya | tdk |  |
| DVT responden | positif | Count | 7 | 18 | 25 |
|  |  | Expected Count | 4.2 | 20.8 | 25.0 |
|  | negatif | Count | 10 | 66 | 76 |
|  |  | Expected Count | 12.8 | 63.2 | 76.0 |
| Total |  | Count | 17 | 84 | 101 |
|  |  | Expected Count | 17.0 | 84.0 | 101.0 |

| **Chi-Square Tests** |  |  |  |  |  |
| --- | --- | --- | --- | --- | --- |
|  | Value | df | Asymptotic Significance (2-sided) | Exact Sig. (2-sided) | Exact Sig. (1-sided) |
| Pearson Chi-Square | 2.960^a^ | 1 | .085 |  |  |
| Continuity Correction^b^ | 1.995 | 1 | .158 |  |  |
| Likelihood Ratio | 2.715 | 1 | .099 |  |  |
| Fisher's Exact Test |  |  |  | .121 | .083 |
| Linear-by-Linear Association | 2.931 | 1 | .087 |  |  |
| N of Valid Cases | 101 |  |  |  |  |

| a. 1 cells (25,0%) have expected count less than 5. The minimum expected count is 4,21. |  |  |  |  |  |
| --- | --- | --- | --- | --- | --- |
| b. Computed only for a 2x2 table |  |  |  |  |  |

| **Symmetric Measures** |  |  |  |  |  |
| --- | --- | --- | --- | --- | --- |
|  |  | Value | Asymptotic Standard Error^a^ | Approximate T^b^ | Approximate Significance |
| Ordinal by Ordinal | Kendall's tau-b | .171 | .110 | 1.494 | .135 |
|  | Kendall's tau-c | .111 | .074 | 1.494 | .135 |
| N of Valid Cases |  | 101 |  |  |  |

| a. Not assuming the null hypothesis. |  |  |  |  |  |
| --- | --- | --- | --- | --- | --- |
| b. Using the asymptotic standard error assuming the null hypothesis. |  |  |  |  |  |

| **Risk Estimate** |  |  |  |
| --- | --- | --- | --- |
|  | Value | 95% Confidence Interval |  |
|  |  | Lower | Upper |
| Odds Ratio for DVT responden (positif / negatif) | 2.567 | .856 | 7.692 |
| For cohort Riwayat jantung = ya | 2.128 | .906 | 4.997 |
| For cohort Riwayat jantung = tdk | .829 | .640 | 1.075 |
| N of Valid Cases | 101 |  |  |

**DVT responden * Riwayat merokok**

| **Crosstab** |  |  |  |  |  |
| --- | --- | --- | --- | --- | --- |
|  |  |  | Riwayat merokok |  | Total |
|  |  |  | ya | tdk |  |
| DVT responden | positif | Count | 1 | 24 | 25 |
|  |  | Expected Count | 1.0 | 24.0 | 25.0 |
|  | negatif | Count | 3 | 73 | 76 |
|  |  | Expected Count | 3.0 | 73.0 | 76.0 |
| Total |  | Count | 4 | 97 | 101 |
|  |  | Expected Count | 4.0 | 97.0 | 101.0 |

| **Chi-Square Tests** |  |  |  |  |  |
| --- | --- | --- | --- | --- | --- |
|  | Value | df | Asymptotic Significance (2-sided) | Exact Sig. (2-sided) | Exact Sig. (1-sided) |
| Pearson Chi-Square | .000^a^ | 1 | .991 |  |  |
| Continuity Correction^b^ | .000 | 1 | 1.000 |  |  |
| Likelihood Ratio | .000 | 1 | .991 |  |  |
| Fisher's Exact Test |  |  |  | 1.000 | .686 |
| Linear-by-Linear Association | .000 | 1 | .991 |  |  |
| N of Valid Cases | 101 |  |  |  |  |

| a. 2 cells (50,0%) have expected count less than 5. The minimum expected count is ,99. |  |  |  |  |  |
| --- | --- | --- | --- | --- | --- |
| b. Computed only for a 2x2 table |  |  |  |  |  |

| **Symmetric Measures** |  |  |  |  |  |
| --- | --- | --- | --- | --- | --- |
|  |  | Value | Asymptotic Standard Error^a^ | Approximate T^b^ | Approximate Significance |
| Ordinal by Ordinal | Kendall's tau-b | .001 | .100 | .012 | .991 |
|  | Kendall's tau-c | .000 | .034 | .012 | .991 |
| N of Valid Cases |  | 101 |  |  |  |

| a. Not assuming the null hypothesis. |  |  |  |  |  |
| --- | --- | --- | --- | --- | --- |
| b. Using the asymptotic standard error assuming the null hypothesis. |  |  |  |  |  |

| **Risk Estimate** |  |  |  |
| --- | --- | --- | --- |
|  | Value | 95% Confidence Interval |  |
|  |  | Lower | Upper |
| Odds Ratio for DVT responden (positif / negatif) | 1.014 | .101 | 10.211 |
| For cohort Riwayat merokok = ya | 1.013 | .110 | 9.308 |
| For cohort Riwayat merokok = tdk | .999 | .912 | 1.096 |
| N of Valid Cases | 101 |  |  |

**DVT responden * jenis kelamin responden**

| **Crosstab** |  |  |  |  |  |
| --- | --- | --- | --- | --- | --- |
|  |  |  | jenis kelamin responden |  | Total |
|  |  |  | P | L |  |
| DVT responden | positif | Count | 21 | 4 | 25 |
|  |  | Expected Count | 18.8 | 6.2 | 25.0 |
|  | negatif | Count | 55 | 21 | 76 |
|  |  | Expected Count | 57.2 | 18.8 | 76.0 |
| Total |  | Count | 76 | 25 | 101 |
|  |  | Expected Count | 76.0 | 25.0 | 101.0 |

| **Chi-Square Tests** |  |  |  |  |  |
| --- | --- | --- | --- | --- | --- |
|  | Value | df | Asymptotic Significance (2-sided) | Exact Sig. (2-sided) | Exact Sig. (1-sided) |
| Pearson Chi-Square | 1.366^a^ | 1 | .242 |  |  |
| Continuity Correction^b^ | .813 | 1 | .367 |  |  |
| Likelihood Ratio | 1.461 | 1 | .227 |  |  |
| Fisher's Exact Test |  |  |  | .295 | .185 |
| Linear-by-Linear Association | 1.353 | 1 | .245 |  |  |
| N of Valid Cases | 101 |  |  |  |  |

| a. 0 cells (,0%) have expected count less than 5. The minimum expected count is 6,19. |  |  |  |  |  |
| --- | --- | --- | --- | --- | --- |
| b. Computed only for a 2x2 table |  |  |  |  |  |

| **Symmetric Measures** |  |  |  |  |  |
| --- | --- | --- | --- | --- | --- |
|  |  | Value | Asymptotic Standard Error^a^ | Approximate T^b^ | Approximate Significance |
| Ordinal by Ordinal | Kendall's tau-b | .116 | .089 | 1.285 | .199 |
|  | Kendall's tau-c | .087 | .067 | 1.285 | .199 |
| N of Valid Cases |  | 101 |  |  |  |

| a. Not assuming the null hypothesis. |  |  |  |  |  |
| --- | --- | --- | --- | --- | --- |
| b. Using the asymptotic standard error assuming the null hypothesis. |  |  |  |  |  |

| **Risk Estimate** |  |  |  |
| --- | --- | --- | --- |
|  | Value | 95% Confidence Interval |  |
|  |  | Lower | Upper |
| Odds Ratio for DVT responden (positif / negatif) | 2.005 | .615 | 6.534 |
| For cohort jenis kelamin responden = P | 1.161 | .931 | 1.447 |
| For cohort jenis kelamin responden = L | .579 | .220 | 1.526 |
| N of Valid Cases | 101 |  |  |

**DVT responden * Malignancy**

| **Crosstab** |  |  |  |  |  |
| --- | --- | --- | --- | --- | --- |
|  |  |  | Malignancy |  | Total |
|  |  |  | ya | tdk |  |
| DVT responden | positif | Count | 2 | 23 | 25 |
|  |  | Expected Count | 1.0 | 24.0 | 25.0 |
|  | negatif | Count | 2 | 74 | 76 |
|  |  | Expected Count | 3.0 | 73.0 | 76.0 |
| Total |  | Count | 4 | 97 | 101 |
|  |  | Expected Count | 4.0 | 97.0 | 101.0 |

| **Chi-Square Tests** |  |  |  |  |  |
| --- | --- | --- | --- | --- | --- |
|  | Value | df | Asymptotic Significance (2-sided) | Exact Sig. (2-sided) | Exact Sig. (1-sided) |
| Pearson Chi-Square | 1.425^a^ | 1 | .233 |  |  |
| Continuity Correction^b^ | .363 | 1 | .547 |  |  |
| Likelihood Ratio | 1.234 | 1 | .267 |  |  |
| Fisher's Exact Test |  |  |  | .255 | .255 |
| Linear-by-Linear Association | 1.411 | 1 | .235 |  |  |
| N of Valid Cases | 101 |  |  |  |  |

| a. 2 cells (50,0%) have expected count less than 5. The minimum expected count is ,99. |  |  |  |  |  |
| --- | --- | --- | --- | --- | --- |
| b. Computed only for a 2x2 table |  |  |  |  |  |

| **Symmetric Measures** |  |  |  |  |  |
| --- | --- | --- | --- | --- | --- |
|  |  | Value | Asymptotic Standard Error^a^ | Approximate T^b^ | Approximate Significance |
| Ordinal by Ordinal | Kendall's tau-b | .119 | .118 | .932 | .352 |
|  | Kendall's tau-c | .040 | .043 | .932 | .352 |
| N of Valid Cases |  | 101 |  |  |  |

| a. Not assuming the null hypothesis. |  |  |  |  |  |
| --- | --- | --- | --- | --- | --- |
| b. Using the asymptotic standard error assuming the null hypothesis. |  |  |  |  |  |

| **Risk Estimate** |  |  |  |
| --- | --- | --- | --- |
|  | Value | 95% Confidence Interval |  |
|  |  | Lower | Upper |
| Odds Ratio for DVT responden (positif / negatif) | 3.217 | .429 | 24.134 |
| For cohort Malignancy = ya | 3.040 | .451 | 20.471 |
| For cohort Malignancy = tdk | .945 | .837 | 1.067 |
| N of Valid Cases | 101 |  |  |

**DVT responden * kriteriaH7 fibrinogen**

| **Crosstab** |  |  |  |  |  |
| --- | --- | --- | --- | --- | --- |
|  |  |  | kriteriaH7 fibrinogen |  | Total |
|  |  |  | tinggi > 400 | normal < = 400 |  |
| DVT responden | positif | Count | 20 | 5 | 25 |
|  |  | Expected Count | 18.1 | 6.9 | 25.0 |
|  | negatif | Count | 53 | 23 | 76 |
|  |  | Expected Count | 54.9 | 21.1 | 76.0 |
| Total |  | Count | 73 | 28 | 101 |
|  |  | Expected Count | 73.0 | 28.0 | 101.0 |

| **Chi-Square Tests** |  |  |  |  |  |
| --- | --- | --- | --- | --- | --- |
|  | Value | df | Asymptotic Significance (2-sided) | Exact Sig. (2-sided) | Exact Sig. (1-sided) |
| Pearson Chi-Square | .989^a^ | 1 | .320 |  |  |
| Continuity Correction^b^ | .543 | 1 | .461 |  |  |
| Likelihood Ratio | 1.036 | 1 | .309 |  |  |
| Fisher's Exact Test |  |  |  | .441 | .234 |
| Linear-by-Linear Association | .979 | 1 | .322 |  |  |
| N of Valid Cases | 101 |  |  |  |  |

| a. 0 cells (,0%) have expected count less than 5. The minimum expected count is 6,93. |  |  |  |  |  |
| --- | --- | --- | --- | --- | --- |
| b. Computed only for a 2x2 table |  |  |  |  |  |

| **Symmetric Measures** |  |  |  |  |  |
| --- | --- | --- | --- | --- | --- |
|  |  | Value | Asymptotic Standard Error^a^ | Approximate T^b^ | Approximate Significance |
| Ordinal by Ordinal | Kendall's tau-b | .099 | .092 | 1.063 | .288 |
|  | Kendall's tau-c | .076 | .072 | 1.063 | .288 |
| N of Valid Cases |  | 101 |  |  |  |

| a. Not assuming the null hypothesis. |  |  |  |  |  |
| --- | --- | --- | --- | --- | --- |
| b. Using the asymptotic standard error assuming the null hypothesis. |  |  |  |  |  |

| **Risk Estimate** |  |  |  |
| --- | --- | --- | --- |
|  | Value | 95% Confidence Interval |  |
|  |  | Lower | Upper |
| Odds Ratio for DVT responden (positif / negatif) | 1.736 | .580 | 5.191 |
| For cohort kriteriaH7 fibrinogen = tinggi > 400 | 1.147 | .897 | 1.467 |
| For cohort kriteriaH7 fibrinogen = normal < = 400 | .661 | .281 | 1.554 |
| N of Valid Cases | 101 |  |  |

**DVT responden * RkriteriaDdimer H1**

| **Crosstab** |  |  |  |  |  |
| --- | --- | --- | --- | --- | --- |
|  |  |  | RkriteriaDdimer H1 |  | Total |
|  |  |  | tinggi | normal |  |
| DVT responden | positif | Count | 8 | 17 | 25 |
|  |  | Expected Count | 11.6 | 13.4 | 25.0 |
|  | negatif | Count | 39 | 37 | 76 |
|  |  | Expected Count | 35.4 | 40.6 | 76.0 |
| Total |  | Count | 47 | 54 | 101 |
|  |  | Expected Count | 47.0 | 54.0 | 101.0 |

| **Chi-Square Tests** |  |  |  |  |  |
| --- | --- | --- | --- | --- | --- |
|  | Value | df | Asymptotic Significance (2-sided) | Exact Sig. (2-sided) | Exact Sig. (1-sided) |
| Pearson Chi-Square | 2.821^a^ | 1 | .093 |  |  |
| Continuity Correction^b^ | 2.098 | 1 | .147 |  |  |
| Likelihood Ratio | 2.881 | 1 | .090 |  |  |
| Fisher's Exact Test |  |  |  | .110 | .073 |
| Linear-by-Linear Association | 2.793 | 1 | .095 |  |  |
| N of Valid Cases | 101 |  |  |  |  |

| a. 0 cells (,0%) have expected count less than 5. The minimum expected count is 11,63. |  |  |  |  |  |
| --- | --- | --- | --- | --- | --- |
| b. Computed only for a 2x2 table |  |  |  |  |  |

| **Symmetric Measures** |  |  |  |  |  |
| --- | --- | --- | --- | --- | --- |
|  |  | Value | Asymptotic Standard Error^a^ | Approximate T^b^ | Approximate Significance |
| Ordinal by Ordinal | Kendall's tau-b | -.167 | .095 | -1.728 | .084 |
|  | Kendall's tau-c | -.144 | .083 | -1.728 | .084 |
| N of Valid Cases |  | 101 |  |  |  |

| a. Not assuming the null hypothesis. |  |  |  |  |  |
| --- | --- | --- | --- | --- | --- |
| b. Using the asymptotic standard error assuming the null hypothesis. |  |  |  |  |  |

| **Risk Estimate** |  |  |  |
| --- | --- | --- | --- |
|  | Value | 95% Confidence Interval |  |
|  |  | Lower | Upper |
| Odds Ratio for DVT responden (positif / negatif) | .446 | .172 | 1.158 |
| For cohort RkriteriaDdimer H1 = tinggi | .624 | .338 | 1.150 |
| For cohort RkriteriaDdimer H1 = normal | 1.397 | .980 | 1.991 |
| N of Valid Cases | 101 |  |  |

**DVT responden * kriteria H7 D-dimer**

| **Crosstab** |  |  |  |  |  |
| --- | --- | --- | --- | --- | --- |
|  |  |  | kriteria H7 D-dimer |  | Total |
|  |  |  | tinggi > =500 | normal < 500 |  |
| DVT responden | positif | Count | 22 | 3 | 25 |
|  |  | Expected Count | 17.3 | 7.7 | 25.0 |
|  | negatif | Count | 48 | 28 | 76 |
|  |  | Expected Count | 52.7 | 23.3 | 76.0 |
| Total |  | Count | 70 | 31 | 101 |
|  |  | Expected Count | 70.0 | 31.0 | 101.0 |

| **Chi-Square Tests** |  |  |  |  |  |
| --- | --- | --- | --- | --- | --- |
|  | Value | df | Asymptotic Significance (2-sided) | Exact Sig. (2-sided) | Exact Sig. (1-sided) |
| Pearson Chi-Square | 5.457^a^ | 1 | .019 |  |  |
| Continuity Correction^b^ | 4.352 | 1 | .037 |  |  |
| Likelihood Ratio | 6.179 | 1 | .013 |  |  |
| Fisher's Exact Test |  |  |  | .024 | .015 |
| Linear-by-Linear Association | 5.403 | 1 | .020 |  |  |
| N of Valid Cases | 101 |  |  |  |  |

| a. 0 cells (,0%) have expected count less than 5. The minimum expected count is 7,67. |  |  |  |  |  |
| --- | --- | --- | --- | --- | --- |
| b. Computed only for a 2x2 table |  |  |  |  |  |

| **Symmetric Measures** |  |  |  |  |  |
| --- | --- | --- | --- | --- | --- |
|  |  | Value | Asymptotic Standard Error^a^ | Approximate T^b^ | Approximate Significance |
| Ordinal by Ordinal | Kendall's tau-b | .232 | .079 | 2.756 | .006 |
|  | Kendall's tau-c | .185 | .067 | 2.756 | .006 |
| N of Valid Cases |  | 101 |  |  |  |

| a. Not assuming the null hypothesis. |  |  |  |  |  |
| --- | --- | --- | --- | --- | --- |
| b. Using the asymptotic standard error assuming the null hypothesis. |  |  |  |  |  |

| **Risk Estimate** |  |  |  |
| --- | --- | --- | --- |
|  | Value | 95% Confidence Interval |  |
|  |  | Lower | Upper |
| Odds Ratio for DVT responden (positif / negatif) | 4.278 | 1.174 | 15.590 |
| For cohort kriteria H7 D-dimer = tinggi > =500 | 1.393 | 1.113 | 1.744 |
| For cohort kriteria H7 D-dimer = normal < 500 | .326 | .108 | .980 |
| N of Valid Cases | 101 |  |  |

**DVT responden * RKriteria DdimerH7**

| **Crosstab** |  |  |  |  |  |
| --- | --- | --- | --- | --- | --- |
|  |  |  | RKriteria DdimerH7 |  | Total |
|  |  |  | tinggi | normal |  |
| DVT responden | positif | Count | 22 | 3 | 25 |
|  |  | Expected Count | 17.6 | 7.4 | 25.0 |
|  | negatif | Count | 49 | 27 | 76 |
|  |  | Expected Count | 53.4 | 22.6 | 76.0 |
| Total |  | Count | 71 | 30 | 101 |
|  |  | Expected Count | 71.0 | 30.0 | 101.0 |

| **Chi-Square Tests** |  |  |  |  |  |
| --- | --- | --- | --- | --- | --- |
|  | Value | df | Asymptotic Significance (2-sided) | Exact Sig. (2-sided) | Exact Sig. (1-sided) |
| Pearson Chi-Square | 4.987^a^ | 1 | .026 |  |  |
| Continuity Correction^b^ | 3.924 | 1 | .048 |  |  |
| Likelihood Ratio | 5.638 | 1 | .018 |  |  |
| Fisher's Exact Test |  |  |  | .042 | .020 |
| Linear-by-Linear Association | 4.937 | 1 | .026 |  |  |
| N of Valid Cases | 101 |  |  |  |  |

| a. 0 cells (,0%) have expected count less than 5. The minimum expected count is 7,43. |  |  |  |  |  |
| --- | --- | --- | --- | --- | --- |
| b. Computed only for a 2x2 table |  |  |  |  |  |

| **Symmetric Measures** |  |  |  |  |  |
| --- | --- | --- | --- | --- | --- |
|  |  | Value | Asymptotic Standard Error^a^ | Approximate T^b^ | Approximate Significance |
| Ordinal by Ordinal | Kendall's tau-b | .222 | .079 | 2.632 | .008 |
|  | Kendall's tau-c | .175 | .067 | 2.632 | .008 |
| N of Valid Cases |  | 101 |  |  |  |

| a. Not assuming the null hypothesis. |  |  |  |  |  |
| --- | --- | --- | --- | --- | --- |
| b. Using the asymptotic standard error assuming the null hypothesis. |  |  |  |  |  |

| **Risk Estimate** |  |  |  |
| --- | --- | --- | --- |
|  | Value | 95% Confidence Interval |  |
|  |  | Lower | Upper |
| Odds Ratio for DVT responden (positif / negatif) | 4.041 | 1.107 | 14.746 |
| For cohort RKriteria DdimerH7 = tinggi | 1.365 | 1.094 | 1.702 |
| For cohort RKriteria DdimerH7 = normal | .338 | .112 | 1.019 |
| N of Valid Cases | 101 |  |  |

**DVT responden * kriteria HDL**

| **Crosstab** |  |  |  |  |  |
| --- | --- | --- | --- | --- | --- |
|  |  |  | kriteria HDL |  | Total |
|  |  |  | rendah <=40 | tinggi >40 |  |
| DVT responden | positif | Count | 9 | 16 | 25 |
|  |  | Expected Count | 5.9 | 19.1 | 25.0 |
|  | negatif | Count | 15 | 61 | 76 |
|  |  | Expected Count | 18.1 | 57.9 | 76.0 |
| Total |  | Count | 24 | 77 | 101 |
|  |  | Expected Count | 24.0 | 77.0 | 101.0 |

| **Chi-Square Tests** |  |  |  |  |  |
| --- | --- | --- | --- | --- | --- |
|  | Value | df | Asymptotic Significance (2-sided) | Exact Sig. (2-sided) | Exact Sig. (1-sided) |
| Pearson Chi-Square | 2.747^a^ | 1 | .097 |  |  |
| Continuity Correction^b^ | 1.922 | 1 | .166 |  |  |
| Likelihood Ratio | 2.587 | 1 | .108 |  |  |
| Fisher's Exact Test |  |  |  | .111 | .085 |
| Linear-by-Linear Association | 2.719 | 1 | .099 |  |  |
| N of Valid Cases | 101 |  |  |  |  |

| a. 0 cells (,0%) have expected count less than 5. The minimum expected count is 5,94. |  |  |  |  |  |
| --- | --- | --- | --- | --- | --- |
| b. Computed only for a 2x2 table |  |  |  |  |  |

| **Symmetric Measures** |  |  |  |  |  |
| --- | --- | --- | --- | --- | --- |
|  |  | Value | Asymptotic Standard Error^a^ | Approximate T^b^ | Approximate Significance |
| Ordinal by Ordinal | Kendall's tau-b | .165 | .107 | 1.506 | .132 |
|  | Kendall's tau-c | .121 | .080 | 1.506 | .132 |
| N of Valid Cases |  | 101 |  |  |  |

| a. Not assuming the null hypothesis. |  |  |  |  |  |
| --- | --- | --- | --- | --- | --- |
| b. Using the asymptotic standard error assuming the null hypothesis. |  |  |  |  |  |

| **Risk Estimate** |  |  |  |
| --- | --- | --- | --- |
|  | Value | 95% Confidence Interval |  |
|  |  | Lower | Upper |
| Odds Ratio for DVT responden (positif / negatif) | 2.288 | .847 | 6.175 |
| For cohort kriteria HDL = rendah <=40 | 1.824 | .913 | 3.643 |
| For cohort kriteria HDL = tinggi >40 | .797 | .582 | 1.092 |
| N of Valid Cases | 101 |  |  |

**DVT responden * kriteria LDL**

| **Crosstab** |  |  |  |  |  |
| --- | --- | --- | --- | --- | --- |
|  |  |  | kriteria LDL |  | Total |
|  |  |  | tinggi >= 130 | normal <130 |  |
| DVT responden | positif | Count | 11 | 14 | 25 |
|  |  | Expected Count | 8.7 | 16.3 | 25.0 |
|  | negatif | Count | 24 | 52 | 76 |
|  |  | Expected Count | 26.3 | 49.7 | 76.0 |
| Total |  | Count | 35 | 66 | 101 |
|  |  | Expected Count | 35.0 | 66.0 | 101.0 |

| **Chi-Square Tests** |  |  |  |  |  |
| --- | --- | --- | --- | --- | --- |
|  | Value | df | Asymptotic Significance (2-sided) | Exact Sig. (2-sided) | Exact Sig. (1-sided) |
| Pearson Chi-Square | 1.282^a^ | 1 | .258 |  |  |
| Continuity Correction^b^ | .792 | 1 | .374 |  |  |
| Likelihood Ratio | 1.254 | 1 | .263 |  |  |
| Fisher's Exact Test |  |  |  | .333 | .186 |
| Linear-by-Linear Association | 1.269 | 1 | .260 |  |  |
| N of Valid Cases | 101 |  |  |  |  |

| a. 0 cells (,0%) have expected count less than 5. The minimum expected count is 8,66. |  |  |  |  |  |
| --- | --- | --- | --- | --- | --- |
| b. Computed only for a 2x2 table |  |  |  |  |  |

| **Symmetric Measures** |  |  |  |  |  |
| --- | --- | --- | --- | --- | --- |
|  |  | Value | Asymptotic Standard Error^a^ | Approximate T^b^ | Approximate Significance |
| Ordinal by Ordinal | Kendall's tau-b | .113 | .102 | 1.093 | .274 |
|  | Kendall's tau-c | .093 | .085 | 1.093 | .274 |
| N of Valid Cases |  | 101 |  |  |  |

| a. Not assuming the null hypothesis. |  |  |  |  |  |
| --- | --- | --- | --- | --- | --- |
| b. Using the asymptotic standard error assuming the null hypothesis. |  |  |  |  |  |

| **Risk Estimate** |  |  |  |
| --- | --- | --- | --- |
|  | Value | 95% Confidence Interval |  |
|  |  | Lower | Upper |
| Odds Ratio for DVT responden (positif / negatif) | 1.702 | .674 | 4.298 |
| For cohort kriteria LDL = tinggi >= 130 | 1.393 | .802 | 2.421 |
| For cohort kriteria LDL = normal <130 | .818 | .560 | 1.196 |
| N of Valid Cases | 101 |  |  |

**DVT responden * kriteria trigliserida**

| **Crosstab** |  |  |  |  |  |
| --- | --- | --- | --- | --- | --- |
|  |  |  | kriteria trigliserida |  | Total |
|  |  |  | tinggi >150 | normal <=150 |  |
| DVT responden | positif | Count | 6 | 19 | 25 |
|  |  | Expected Count | 4.7 | 20.3 | 25.0 |
|  | negatif | Count | 13 | 63 | 76 |
|  |  | Expected Count | 14.3 | 61.7 | 76.0 |
| Total |  | Count | 19 | 82 | 101 |
|  |  | Expected Count | 19.0 | 82.0 | 101.0 |

| **Chi-Square Tests** |  |  |  |  |  |
| --- | --- | --- | --- | --- | --- |
|  | Value | df | Asymptotic Significance (2-sided) | Exact Sig. (2-sided) | Exact Sig. (1-sided) |
| Pearson Chi-Square | .586^a^ | 1 | .444 |  |  |
| Continuity Correction^b^ | .221 | 1 | .638 |  |  |
| Likelihood Ratio | .562 | 1 | .453 |  |  |
| Fisher's Exact Test |  |  |  | .556 | .311 |
| Linear-by-Linear Association | .580 | 1 | .446 |  |  |
| N of Valid Cases | 101 |  |  |  |  |

| a. 1 cells (25,0%) have expected count less than 5. The minimum expected count is 4,70. |  |  |  |  |  |
| --- | --- | --- | --- | --- | --- |
| b. Computed only for a 2x2 table |  |  |  |  |  |

| **Symmetric Measures** |  |  |  |  |  |
| --- | --- | --- | --- | --- | --- |
|  |  | Value | Asymptotic Standard Error^a^ | Approximate T^b^ | Approximate Significance |
| Ordinal by Ordinal | Kendall's tau-b | .076 | .105 | .718 | .473 |
|  | Kendall's tau-c | .051 | .072 | .718 | .473 |
| N of Valid Cases |  | 101 |  |  |  |

| a. Not assuming the null hypothesis. |  |  |  |  |  |
| --- | --- | --- | --- | --- | --- |
| b. Using the asymptotic standard error assuming the null hypothesis. |  |  |  |  |  |

| **Risk Estimate** |  |  |  |
| --- | --- | --- | --- |
|  | Value | 95% Confidence Interval |  |
|  |  | Lower | Upper |
| Odds Ratio for DVT responden (positif / negatif) | 1.530 | .512 | 4.574 |
| For cohort kriteria trigliserida = tinggi >150 | 1.403 | .597 | 3.300 |
| For cohort kriteria trigliserida = normal <=150 | .917 | .719 | 1.169 |
| N of Valid Cases | 101 |  |  |

**DVT responden * kriteria tot.col**

| **Crosstab** |  |  |  |  |  |
| --- | --- | --- | --- | --- | --- |
|  |  |  | kriteria tot.col |  | Total |
|  |  |  | tinggi >200 | normal <=200 |  |
| DVT responden | positif | Count | 10 | 15 | 25 |
|  |  | Expected Count | 9.4 | 15.6 | 25.0 |
|  | negatif | Count | 28 | 48 | 76 |
|  |  | Expected Count | 28.6 | 47.4 | 76.0 |
| Total |  | Count | 38 | 63 | 101 |
|  |  | Expected Count | 38.0 | 63.0 | 101.0 |

| **Chi-Square Tests** |  |  |  |  |  |
| --- | --- | --- | --- | --- | --- |
|  | Value | df | Asymptotic Significance (2-sided) | Exact Sig. (2-sided) | Exact Sig. (1-sided) |
| Pearson Chi-Square | .080^a^ | 1 | .777 |  |  |
| Continuity Correction^b^ | .002 | 1 | .964 |  |  |
| Likelihood Ratio | .080 | 1 | .778 |  |  |
| Fisher's Exact Test |  |  |  | .815 | .478 |
| Linear-by-Linear Association | .079 | 1 | .778 |  |  |
| N of Valid Cases | 101 |  |  |  |  |

| a. 0 cells (,0%) have expected count less than 5. The minimum expected count is 9,41. |  |  |  |  |  |
| --- | --- | --- | --- | --- | --- |
| b. Computed only for a 2x2 table |  |  |  |  |  |

| **Symmetric Measures** |  |  |  |  |  |
| --- | --- | --- | --- | --- | --- |
|  |  | Value | Asymptotic Standard Error^a^ | Approximate T^b^ | Approximate Significance |
| Ordinal by Ordinal | Kendall's tau-b | .028 | .100 | .280 | .779 |
|  | Kendall's tau-c | .024 | .084 | .280 | .779 |
| N of Valid Cases |  | 101 |  |  |  |

| a. Not assuming the null hypothesis. |  |  |  |  |  |
| --- | --- | --- | --- | --- | --- |
| b. Using the asymptotic standard error assuming the null hypothesis. |  |  |  |  |  |

| **Risk Estimate** |  |  |  |
| --- | --- | --- | --- |
|  | Value | 95% Confidence Interval |  |
|  |  | Lower | Upper |
| Odds Ratio for DVT responden (positif / negatif) | 1.143 | .453 | 2.885 |
| For cohort kriteria tot.col = tinggi >200 | 1.086 | .618 | 1.907 |
| For cohort kriteria tot.col = normal <=200 | .950 | .661 | 1.366 |
| N of Valid Cases | 101 |  |  |
